# Selection Signature Analysis Implicates the *PC1/PCSK1* Region for Chicken Abdominal Fat Content

**DOI:** 10.1371/journal.pone.0040736

**Published:** 2012-07-11

**Authors:** Hui Zhang, Xiaoxiang Hu, Zhipeng Wang, Yuandan Zhang, Shouzhi Wang, Ning Wang, Li Ma, Li Leng, Shengwen Wang, Qigui Wang, Yuxiang Wang, Zhiquan Tang, Ning Li, Yang Da, Hui Li

**Affiliations:** 1 Key Laboratory of Chicken Genetics and Breeding, Ministry of Agriculture, Harbin, People's Republic of China; 2 College of Animal Science and Technology, Northeast Agricultural University, Harbin, People's Republic of China; 3 College of Biological Science, China Agricultural University, Beijing, People's Republic of China; 4 Animal Genetics and Breeding Unit, University of New England, Armidale, New South Wales, Australia; 5 Department of Animal Science, University of Minnesota, Saint Paul, Minnesota, United States of America; Rutgers University, United States of America

## Abstract

We conducted a selection signature analysis using the chicken 60k SNP chip in two chicken lines that had been divergently selected for abdominal fat content (AFC) for 11 generations. The selection signature analysis used multiple signals of selection, including long-range allele frequency differences between the lean and fat lines, long-range heterozygosity changes, linkage disequilibrium, haplotype frequencies, and extended haplotype homozygosity. Multiple signals of selection identified ten signatures on chromosomes 1, 2, 4, 5, 11, 15, 20, 26 and Z. The 0.73 Mb *PC1/PCSK1* region of the Z chromosome at 55.43-56.16 Mb was the most heavily selected region. This region had 26 SNP markers and seven genes, *Mar-03*, *SLC12A2*, *FBN2*, *ERAP1*, *CAST*, *PC1/PCSK1* and *ELL2*, where *PC1/PCSK1* are the chicken/human names for the same gene. The lean and fat lines had two main haplotypes with completely opposite SNP alleles for the 26 SNP markers and were virtually line-specific, and had a recombinant haplotype with nearly equal frequency (0.193 and 0.196) in both lines. Other haplotypes in this region had negligible frequencies. Nine other regions with selection signatures were *PAH-IGF1*, *TRPC4*, *GJD4-CCNY*, *NDST4*, *NOVA1*, *GALNT9*, the *ESRP2-GALR1* region with five genes, the *SYCP2-CADH4* with six genes, and the *TULP1-KIF21B* with 14 genes. Genome-wide association analysis showed that nearly all regions with evidence of selection signature had SNP effects with genome-wide significance (P<10^–6^) on abdominal fat weight and percentage. The results of this study provide specific gene targets for the control of chicken AFC and a potential model of AFC in human obesity.

## Introduction

The chicken (*Gallus gallus*) is an important model organism that bridges the evolutionary gap between mammals and other vertebrates [1]. Research on human obesity typically uses body mass index (BMI) as the phenotypic measure of obesity [2]–[12]. However, BMI is affected by variations in the entire body, including bones, muscles and body fat, and is not specific for abdominal fat, a major problem in obese people. Although indirect measures of human abdominal fat are available [13], [14], direct measures are unavailable. In chickens, abdominal fat weight (AFW) can be measured directly, and experiments could be designed to identify genetic variants associated with abdominal fat content (AFC). The results from this type of experiment may provide useful comparative information for human obesity research and lead to genetic improvement for reduced abdominal fat in chickens.

Selection for rapid growth in chickens has always been accompanied by increased fat deposition [15], [16]. Excessive fat deposition can decrease feed efficiency and cause consumer rejection of the meat [17], and cause difficulties in meat processing [18]. The measurements of fatness are often laborious and expensive by slaughtering birds, which prevents genetic selection on the basis of an individual's measures of fatness. Knowledge of the genetic factors associated with fatness will facilitate genetic selection using genetic markers without the necessity to assess the phenotype of the selected individuals. Genome-wide association studies (GWAS) provide a powerful approach to the identification of the genetic factors associated with phenotypes. However, GWAS is affected by variations in phenotypic measures and by genetic drift and hitchhiking of the genome. In contrast, selection signature analysis [19]–[21] does not rely on phenotypic measurements and could be a promising approach to address the statistical noise from drift and hitchhiking. An integrated analysis of selection signature and GWAS provides a new approach that has the strengths of both methods. A joint analysis of selection signature and GWAS using two divergent chicken lines has been reported [22].

The goal of this study was to identify genome changes and genes associated with selection for high and low AFC using multiple signals of the selection signature in an experimental chicken population that had undergone 11 generations of divergent selection for high and low AFC. The GWAS analysis was used to assess the phenotypic effects of the selection signatures identified in this study and was also used to identify likely causal locations of the selection signatures with dense gene coverage.

## Materials and Methods

### Ethics statement

All animal work was conducted according to the guidelines for the care and use of experimental animals established by the Ministry of Science and Technology of the People's Republic of China (Approval number: 2006-398), and was approved by the Laboratory Animal Management Committee of Northeast Agricultural University.

### Animals

**Figure 1:**
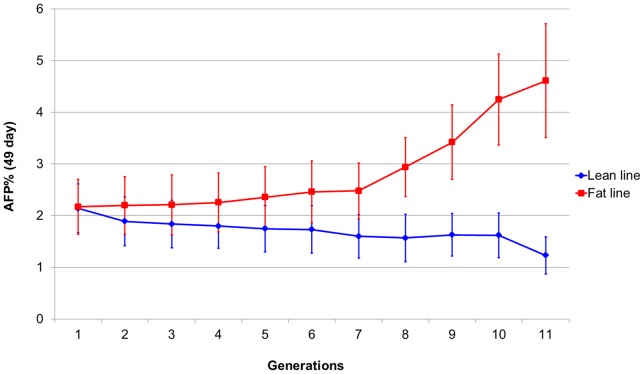
Phenotypic changes after 11 generations of divergent selection for high and low abdominal fat content.

The broilers used in this study were from two Northeast Agricultural University (NEAU) broiler lines divergently selected for AFC (NEAUHLF). The NEAUHLF lines have been selected since 1996 using abdominal fat percentage (AFP  =  AFW/body weight) and plasma very low-density lipoprotein (VLDL) concentration as selection criteria. The G_0_ generation of NEAUHLF came from the same grandsire line originating from the Arbor Acres broiler, which was then divided into two lines according to their plasma VLDL concentration at 7 weeks of age. The G_0_ birds were mated (one sire: four dams) to produce 25 half-sib families for each line, with an average of 70 G_1_ offspring per family in two hatches. From G_1_ to G_11_, the birds of each line were raised in two hatches and housed in pens with five birds per cage. Plasma VLDL concentrations were measured for all male birds, which had free access to feed and water at 7 weeks, and the AFP of the male birds in the first hatch was measured after slaughter at 7 weeks. Sib birds from the families with lower (lean line) or higher (fat line) AFP than the average value for the population were selected as candidates for breeding, considering the plasma VLDL concentration and the body weights of male birds in the second hatch and the egg production of female birds in both hatches. These birds were kept under the same environmental conditions and had free access to feed and water. Commercial corn-soybean-based diets that met all National Research Council (NRC) requirements were provided. From hatch to 3 weeks of age, the birds received a starter feed (3,000 kal ME = kg and 210 g = kg CP) and from 4 weeks of age to slaughter the birds were fed a grower diet (3,100 kal ME = kg and 190 g = kg CP). A total of 475 individuals (203 from the fat line and 272 from the lean line) from generation 11 of NEAUHLF were used in this study. The AFP of the fat line was 3.75 times that of the lean line at generation 11 (Table 1), and the phenotypic (AFP) changes over the 11 generations are shown in Figure 1.

**Table 1:** Mean values and standard deviations of abdominal fat weight (AFW), abdominal fat percentage (AFP) and body weight at 7 weeks of age in the lean and fat lines after 11 generations of divergent selection.

Traits	Lean line (203)	Fat line (272)
AFW (g)	30.09^B^±10.07	110.29^A^±27.87
AFP (%)	1.23^B^±0.37	4.62^A^±1.10
BW7 (g)	2417.52±244.64	2384.33±201.42

AB significant difference between the two lines at *P*<0.05.

### SNP selection and genotyping

The 60k chip had a total of 57,636 SNPs, and 45,578 SNPs with a minor allele frequency (MAF) of 5% or greater and a call rate of 95% in the combined sample of the lean and fat lines were selected for use in this study. Individuals with pedigree error or 5% or more missing SNP genotypes were removed. Of the 45,578 SNPs, 45,005 had known chromosome locations and were distributed across 28 autosomes, the Z chromosome and two linkage groups (LGE22C19W28_E50C23 and LGE64). The number of SNPs per chromosome ranged from 2 to 7,135 with a mean distance of 22.51 kb between adjacent SNPs (Table 2). Genomic DNA was isolated from venous blood samples using a phenol-chloroform method from 20 μL of venous blood collected in EDTANa_2_-coated tubes and stored at −20°C. Genotyping of the chicken 60k SNP chips from Illumina Inc. was performed by DNA LandMarks Inc., Quebec, Canada, using 75 μL of approximately 50 ng/μL genomic DNA.

**Table 2:** Distribution of SNP markers and relevant statistics from selection signature analysis and genome-wide association analysis by chromosome.

Chr	No. SNPs	Average Distance (bp)	No. SNPs/Mb	Average AFD^1^	Fixed alleles in lean line	Fixed alleles in fat line	LD (r^2^) in lean line	LD (r^2^) in fat line	Significant SNPs^2^ for AFW	Significant SNPs^2^ for AFP
1	7135	28136	35.51	0.18	120 (1.68)^3^	165 (2.31)^3^	0.29	0.29	38	28
2	5290	29217	34.25	0.20	121 (2.29)	103 (1.95)	0.32	0.28	63	62
3	4081	27855	35.91	0.21	88 (2.16)	99 (2.43)	0.29	0.28	26	27
4	3313	28423	35.18	0.20	74 (2.23)	70 (2.11)	0.31	0.28	83	78
5	2170	28662	34.87	0.19	47 (2.17)	46 (2.12)	0.31	0.28	14	14
6	1714	20920	47.83	0.20	45 (2.63)	24 (1.40)	0.27	0.23	5	10
7	1769	21576	46.35	0.20	66 (3.73)	19 (1.07)	0.29	0.27	4	6
8	1394	21985	45.52	0.21	30 (2.15)	28 (2.01)	0.30	0.28	11	7
9	1168	20585	48.62	0.20	31 (2.65)	28 (2.40)	0.27	0.25	6	5
10	1297	17300	57.85	0.19	20 (1.54)	37 (2.85)	0.26	0.26	10	8
11	1196	18302	54.68	0.21	41 (3.43)	34 (2.84)	0.36	0.30	23	21
12	1324	15455	64.75	0.20	31 (2.34)	15 (1.13)	0.30	0.25	12	12
13	1128	16253	61.58	0.22	25 (2.22)	36 (3.19)	0.29	0.27	4	2
14	984	16036	62.42	0.22	28 (2.85)	30 (3.05)	0.26	0.25	7	5
15	1010	12810	78.14	0.22	24 (2.38)	27 (2.67)	0.27	0.25	24	17
16	12	34823	72.07	0.20	0 (0.00)	1 (8.33)	0.44	0.30	0	0
17	844	12590	79.52	0.20	23 (2.73)	19 (2.25)	0.23	0.21	2	2
18	845	12898	77.62	0.20	15 (1.78)	29 (3.43)	0.23	0.22	1	2
19	804	12321	81.27	0.17	7 (0.87)	11 (1.37)	0.19	0.22	3	5
20	1460	9541	104.89	0.24	18 (1.23)	54 (3.70)	0.26	0.25	57	33
21	726	9483	105.60	0.20	19 (2.62)	7 (0.96)	0.27	0.22	1	1
22	295	13234	75.82	0.20	8 (2.71)	7 (2.37)	0.27	0.24	3	4
23	577	10456	95.81	0.22	10 (1.73)	9 (1.56)	0.23	0.22	0	1
24	676	9229	108.51	0.22	23 (3.40)	15 (2.22)	0.24	0.23	2	5
25	170	11930	84.32	0.20	3 (1.76)	5 (2.94)	0.21	0.17	1	1
26	617	8169	122.62	0.23	23 (3.73)	17 (2.76)	0.21	0.24	23	16
27	472	10271	97.57	0.20	23 (4.87)	20 (4.24)	0.24	0.19	6	6
28	563	7932	126.30	0.17	7 (1.24)	12 (2.13)	0.20	0.19	0	1
LGE22	103	8651	116.72	0.22	5 (4.85)	1 (0.97)	0.28	0.19	0	0
LGE64	2	2289	873.74	0.33	0 (0.00)	0 (0.00)	0.33	0.84	0	0
Z	1842	40472	24.70	0.24	120 (6.51)	192 (10.42)	0.43	0.44	49	50
UN^4^	606	/	/	0.20	22 (3.63)	26 (4.29)	/	/	2	2

1 This is the average AFD of each single SNP marker between the lean and fat lines.

2 This is the number of SNPs with P values <10^−6.56^ for association effects on AFW and AFP.

3 The number in () is the percentage of fixed alleles on the chromosome, i.e., (No. of fixed alleles)/(No. of SNPs) ×100.

4 These SNPs are not assigned to any chromosomes.

### Statistical analysis

#### Selection signature analysis

The selection signature analysis used a combination of various signals of selection, including long-range heterozygosity changes [19], long-range allele frequency differences (AFD) and standardized AFD between the lean and fat lines, linkage disequilibrium (LD) and haplotype analyses [23], and extended haplotype homozygosity (EHH) analysis [24]. Heterozygosity and AFD measures were used for the first screening of selection signatures, and LD, haplotype frequencies and EHH were analyzed as additional evidence of selection signatures and as indications of whether selection had occurred in one line or both lines. The LD and haplotype analyses used Haploview [23], and the extended haplotype homozygosity (EHH) analysis was carried out using Sweep-1.1 [24]. Phased genotypic data as input files for Sweep 1.1 were produced using FASTPHASE [25].

For long-range AFD and heterozygosity measures, we used 0.5 Mb sliding windows of SNP markers as the genome length. Two long-range heterozygosity measures were calculated following the method in [19]: standardized heterozygosity in the lean line (Z_lean) and standardized heterozygosity in the fat line (Z_fat). For each sliding window, we also calculated the AFD and standardized AFD (Z_AFD) between the lean and fat lines. For each chromosome, the Z_AFD between the two lines used the chromosome mean and standard deviation of the AFD values, while Z_lean and Z_fat each used within-line mean and standard deviation of the heterozygosity values of the entire chromosome. This type of within-line standardization is more conservative than across-line standardization using the pooled mean and standard deviation of the chromosome over the two lines. The criterion for declaring selection was the use of extreme AFD and extreme standardized AFD and heterozygosity values, following the approach in [19]. Threshold values of the above measures for declaring significance were AFD ≥0.44, Z_AFD  = 4.0, and Z_lean  =  Z_fat  = ±5.0, and the percentages of markers above these threshold values were 0.27%, 0.09%, 0.11%, and 0.02%, respectively.

The two AFD measures (AFD and Z_AFD) were used to compensate for the weakness of the three measures of heterozygosity (Z_lean and Z_fat) in cases of ‘p-q sweep’, where heterozygosity measures are expected to fail to detect genome changes due to selection. Let p_0_ and q_0_ represent the allele frequencies of alleles 1 and 2 in the unselected population, and let p_t_ and q_t_ represent the allele frequencies for the same alleles after t generations of selection. Then, heterozygosity has no change at generation t if p_t_ = q_0_ and q_t_ = p_0_ (p-q sweep) assuming Hardy-Weinberg equilibrium. Therefore, the use of heterozygosity could miss significant allele frequency changes that result in a p-q sweep. For this reason, increases in heterozygosity (rather than decreases only) were also considered (in addition to AFD and Z_AFD) when identifying chromosome regions subjected to selection, because increases in heterozygosity could also be a result of substantial allele frequency changes when the initial population had extreme frequencies.

#### SNP association analysis

The significance testing of SNP effects on AFW and AFP used four methods, the GLS-LS version of EPISNP [26], [27] was the main method for reporting GWAS results, and PLINK [28] was used as a secondary method for reporting GWAS results. The GLS-LS version uses generalized least squares (GLS) to estimate fixed non-genetic effects and uses least squares (LS) for testing SNP effects after removing the GLS estimates of fixed non-genetic effects from the phenotypic values. The statistical model was Y =  SNP + f + e, where Y was the phenotype value, SNP was the SNP marker effect, f was a random family effect to account for sib correlation, and e was the residual effect. Each SNP was tested for two effects, additive and dominance effects. The genome-wide 5% type-I error with the Bonferroni correction was considered to indicate genome-wide significance. For two traits (AFW and AFP), two effects per test (additive and dominance effects) and 45,578 SNP markers, the threshold *P*-value for declaring genome-wide significance was (0.05)/[(2)(2)(45,578)]  = 2.74×10^−7^ = 10^−6.56^. The statistical model for PLINK was the same as that for the GLS-LS version of EPISNP, except that the family effect was treated as a fixed effect. Gene locations were based on Ensembl [29] and NCBI [12]. Full gene names from Ensembl and NCBI for the candidate genes identified in Table 3 and Figure S1 are given in Table S1.

**Table 3:** Selection signatures of 11 generations of divergent selection for abdominal fat content in chickens.

Chr	Peak positions (bp)	AFD	Z_AFD	Z_lean	Z_fat	Nearest gene	Most significant SNP	*P*-value	Trait
1	57053708–57160808	0.26	1.42	0.76	−*5.99*	*PAH* (150 kb upstream of *IGF1*)	Gga_rs14828014	2.29×10^−7^	AFP
1	176076327–176286631	0.42	*4.23*	−3.04	−1.22	*TRPC4*	GGaluGA055731	1.21×10^−8^ 2.28×10^−8^	AFW AFP
2	12476376–12801632	*0.47*	*4.15*	−4.79	0.17	*GJD4, CCNY*	Gga_rs14139748	7.46×10^−9^ 2.43×10^−8^	AFW AFP
4	57429243–57788219	*0.50*	*4.26*	−3.40	1.14	*NDST4*	Gga_rs16416191	2.89×10^−8^ 9.18×10^−8^	AFW AFP
5	35024640–35653631	0.42	*4.40*	−4.05	0.35	*NOVA1*	GGaluGA282591	1.25×10^−7^	AFW
11	3196613–3402854	0.32	1.70	−*5.80*	1.12	*ESRP2-GALR1* (5 genes), 0.2 Mb upstream of *MMP2* and 1.45 Mb upstream of *FTO*	Gga_rs14959270	4.57×10^−8^ 2.85×10^−8^	AFW AFP
15	2155345–2495806	*0.44*	3.46	−1.25	−3.06	*GALNT9*	Gga_rs14087994	1.66×10^−10^ 2.21×10^−10^	AFW AFP
20	6829844–7289095	*0.46*	2.94	1.67	−1.02	*SYCP2-CADH4* (6genes)	Gga_rs14274917	1.03×10^−9^ 1.00×10^−8^	AFW AFP
26	55909–288827	*0.50*	*4.50*	−3.58	*5.02*	*TULP1-KIF21B* (14 genes)	Gga_rs14416336	1.71×10^−7^ 2.26×10^−7^	AFW AFP
Z	55428021–56164905	*0.75*	*4.98*	−3.7	−1.13	*Mar-03*, *SLC12A2*, *FBN2*, *ERAP1*, *CAST*, * PC1/PCSK1*, * ELL2*	Gga_rs14751538	3.09×10^−8^ 4.44×10^−8^	AFW AFP

AFD  =  The average of |(frequency of “allele 1” in the lean line) – (frequency of “allele 1” in the fat line)| for all SNP markers in 0.5 Mb sliding windows, where “allele 1”  =  “*A*” for *A/C*, *A/G*, *A/T*,  =  “*C*” for *C/G* and *C/T*, and  =  “*G*” for *G/T*; Z_AFD  =  standardized AFD in 0.5 Mb sliding windows; Z_lean  =  standardized average SNP heterozygosity in 0.5 Mb sliding windows in the lean line; Z_fat  =  standardized average SNP heterozygosity in 0.5 Mb sliding windows in the fat line. Italic indicates evidence of selection signature.

## Results and Discussion

**Figure 2:**
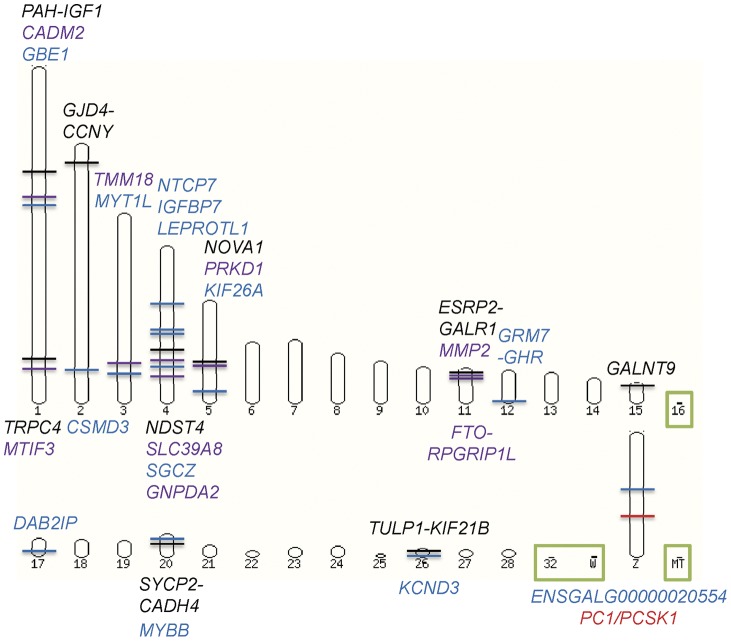
Genome view of selection signatures and SNP effects associated with chicken abdominal fat content. Black: selection signature and gene in the selection signature (nearly all these regions had SNP effects with genome-wide significance); Blue: chromosome region with highly significant SNP effects but not declared as a selection signature. Purple: human obesity gene nearest to a selection signature or a region with highly significant SNP effects for abdominal fat weight (AFW) and abdominal fat percentage (AFP); Red: human obesity gene inside selection signature; Green box: not studied.

Multiple signals of selection identified ten selection signatures on chromosomes 1, 2, 4, 5, 11, 15, 20, 26 and Z as signatures of selection for high and low AFC in the 11 generations. The candidate genes and regions with multiple genes in the selection signatures were *PC1*/*PCSK1*, *PAH-IGF1*, *TRPC4*, *GJD4-CCNY*, *NDST4*, *NOVA1*, *GALNT9*, the *ESRP2-GALR1* region with five genes, the *SYCP2-CADH4* region with six genes, and the *TULP1-KIF21B* region with 14 genes. Table 3 describes general characteristics of these selection signatures. A summary of the selection signatures and chromosome regions with highly significant SNP effects is given in Figure 2, which also displays the locations of human obesity genes [2–12; Table S2] on the chicken genome. Six selection signatures on chromosomes 1, 2, 4, 5 and 15 each involved one or two genes while the remaining four signatures on chromosomes 11, 20, 26 and Z each involved 6–14 genes.

Long-range AFD between the lean and fat lines, Z_AFD, Z_lean, and Z_fat in 0.5 Mb sliding windows of SNP markers were used for initial genome-wide screening of selection signatures (Figure S2). Linkage disequilibrium (LD) patterns [23] (Figure S3), extended haplotype heterozygosity (EHH) analysis [24] (Figure S4) and haplotype analyses [23] (Table S3) further confirmed the initial evidence of selection signatures, provided evidence of whether selection had occurred in one line or both lines, and modified the sizes of some selection signatures. In addition, the EHH analysis added information about a specific gene or genes that may have been subject to selection.

### The *PC1*/*PCSK1* region of chromosome Z: the most heavily selected chromosome region

**Figure 3:**
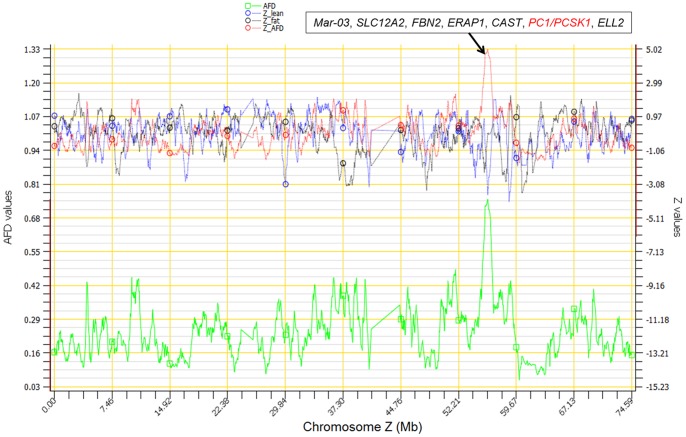
Long-range AFD and heterozygosity of chromosome Z.

The most significant region in the 11 generations of divergent selection for high and low AFC was the 0.73 Mb region of 55.43–56.16 Mb of chromosome Z, with 26 intronic SNP markers. This region had the largest long-range AFD and the largest Z_AFD between the lean and fat lines for all chromosomes, with an average AFD greater than 0.70 and peak Z_AFD of 4.98 in 0.5 Mb sliding windows of SNP markers (Figure 3, Table 3). No other selection signature had such large average AFD, particularly for a 0.73 Mb distance with 26 SNP markers. Single locus AFD between the lean and fat lines for the 26 SNP markers in this region were in the range of 0.68–0.78, and dropped to 0.11–0.46 outside this 0.73 Mb region, although large AFD values existed further away from this region. This 0.73 Mb region had seven genes, *Mar-03*, *SLC12A2*, *FBN2*, *ERAP1*, *CAST*, *PC1*, and *ELL2*, noting that only a downstream portion of *Mar-03* and an upstream portion of *ELL2* were in this region. Of these seven genes, only *PC1* is known to have biological functions highly relevant to AFC. In humans, *PC1* is also known as PCSK1 [12]. This gene plays a key role in regulating insulin biosynthesis [12], and is on the leptin/melanocortin pathway that is related to obesity [30]. Variants in *PCSK1* have been found to be associated with human obesity [8]–[12]. The complete chicken PC1 DNA sequence is 56,014,540–56,043,792 bp in length. This sequence matches 16 segments of the human PCSK1 DNA sequence [29] and has 79.1% identity with the human PCSK1 DNA sequence [31]. No match or identity between the chicken PC1 and any other human DNA sequence was found. For this reason and considering the known relevance of *PC1*/*PCSK1* to obesity, we named this 0.73 Mb seven-gene region as the *PC1*/*PCSK1* region.

**Figure 4:**
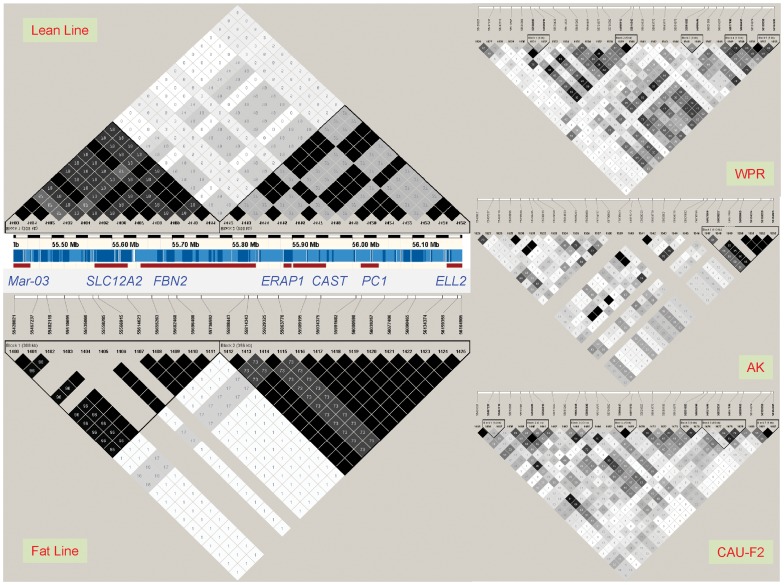
Linkage disequilibrium patterns of the chromosome Z selection signature at 55.43–56.16 Mb. WPR: a recessive white line of White Plymouth Rock chicken from France (n = 94). AK: Anak, a commercial broiler chicken introduced from Israel (n = 51). CAU-F2: an F2 resource population produced from reciprocal crosses of Silky Fowl and White Plymouth Rock at China Agricultural University (n = 112).

The haplotype and LD analyses showed that the two lines had two main haplotypes and shared a common recombinant haplotype in two haplotype blocks separated by the recombination point at 55,736,592–55,808,443 bp, shown between markers 1,141 and 1,142 in Figure 4. The main haplotype in the lean line (Haplotype 1 in Table 4) had a frequency of 0.683 and did not exist in the fat line. The main haplotype in the fat line (Haplotype 2 in Table 4) had a high frequency of 0.75 and a low frequency of 0.017 in the lean line, and these two main haplotypes had opposite alleles at each of the 26 SNP markers (Table 4). The recombinant haplotype (Haplotype 3 in Table 4) in each line was due to recombination between Haplotypes 1 and 2 (between markers 1,141 and 1,142 in Figure 4) and had similar frequencies in the two lines (0.193 in the lean line and 0.196 in the fat line). This indicates that the recombinant type is not of great importance to either line and that both sides of the recombination point had causal effects. The lean line had three other haplotypes with a combined frequency of 0.12, and the fat line also had three other haplotypes with a combined frequency of 0.0524 (Table 4).

**Table 4:** Haplotypes and frequencies of the 0.73 Mb region at 55.43–56.16 Mb of chromosome Z with 26 SNP markers in the lean and fat lines.

Haploptype	Block 1	Block 2	Haplotype frequency in lean line	Haplotype frequency in fat line
1	AAGGAAGGACGA	AGAGGGAAGAAAAG	*0.683*	0
2	GGAAGCAACAAG	GAGAAAGGAGGGGA	0.017	*0.750*
3	GGAAGCAACAAG	AGAGGGAAGAAAAG	0.193	0.196
4	AAGAAAGGACGA	AGAGGGAAGAAAAG	0.063	0
5	AAGAGAAACCAG	GAAGAAAGAGGAAG	0.043	0
6	AAGAGAAGACGA	GAAGAAGGAGGGGA	0	0.052
7	GGAAGCAACAAG	GAAGAAGGAGGGGA	0	0.0002
8	GGGAGAAGACGA	GAAGAAGGAGGGGA	0	0.0002

**Figure 5:**
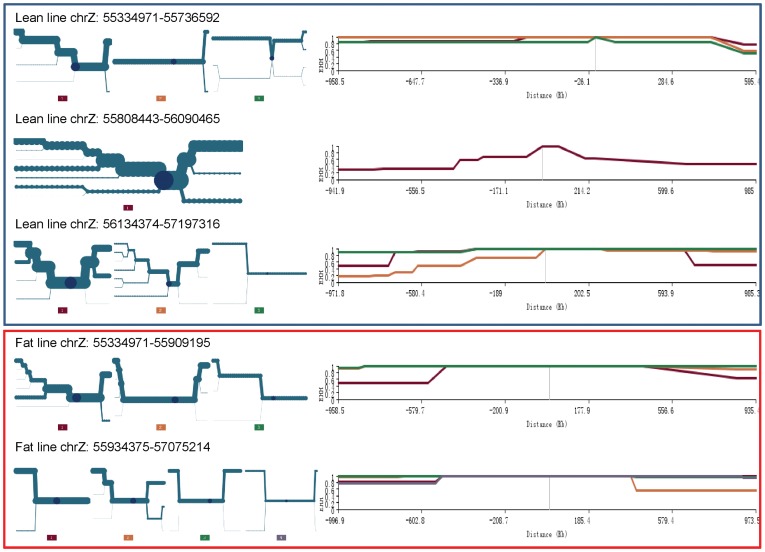
Extended haplotype heterozygosity in the chromosome Z selection signature at 55.43–56.16 Mb.

The LD and EHH results also showed that this 0.73 Mb region was potentially subject to selection, and that the fat line had stronger selection than the lean line. The fat line had strong LD values, r^2^ = 1 in both blocks for adjacent SNPs, except for one SNP pair in the first block and two SNP pairs in the second block, and had three fixed SNP markers in the first block (Figure 4). The fat line had higher r^2^ values and more fixed SNP markers than the lean line, which did not have fixed SNP markers in both haplotype blocks, indicating that the 0.73 Mb region had greater selection pressure in the fat line than in the lean line. Note that the default algorithm for defining haplotype blocks [32] implemented by Haploview [23] defined different haplotype blocks (Figure S3A) from those shown in Figure 4. The results of EHH analysis (Figure 5) were in agreement with the LD analysis. For the lean line, EHH analysis defined essentially two haplotype blocks separated by the recombination point between the lean and fat haplotypes (Haplotype 1 and 2 in Table 4). The third haplotype block covered only about 30 kb of the selection signature. For the fat line, two haplotype blocks were defined for a total distance that exceeded the selection signature distance by about 910.309 kb. Therefore the two haplotype blocks in the fat line covered nearly 1 Mb more than the first two haplotype blocks in the lean line. The EHH values of both haplotype blocks in the fat line were also higher and were at 100% for longer distances than those of the first two haplotype blocks in the lean line. These EHH results indicate that the fat line was subjected to stronger selection pressure in this 0.73 Mb region. The LD values of the lean and fat lines were obviously stronger than those of three chicken lines without selection for abdominal fat (White Plymouth Rock (WPR), Anak (AK) and an F_2_ population constructed by China Agricultural University (CAU-F2), shown on the right of Figure 4), which indicates that this chromosome region had been subjected to strong selection in the lean and fat lines.

### The chromosome 1 selection signature at the *PAH-IGF1* region

This selection signature was the only selection signature identified by decreased heterozygosity in the fat line (Z_fat  = −5.99, Table 3) in the 57.05–57.16 Mb region that included *PAH* and was about 150 kb upstream of *IGF1*, a gene known to be associated with fatness in chicken [33], [34]. The 60k chicken SNP chip had two SNP markers in *IGF1* but neither met the minor allele frequency (MAF) requirement (MAF >5%). Consequently, no SNP marker inside *IGF1* was present in this study and it is unknown whether *IGF1* was part of the *PAH* selection signature. However, four SNP markers that were immediately upstream of *IGF1* were fixed, and another three SNPs were either fixed or nearly fixed with allele frequencies of 0.015, 1.0 and 0.983 in the fat line, while the AFD between the two lines for these seven markers were 0.14–0.53, indicating that *IGF1* could have been selected for high AFC. The LD signals and EHH values in the region spanning *IGF1* were considerably stronger in the fat line than in the lean line (Figure S3B, Figure S4A), further indicating potential involvement of *IGF1* in chicken AFC. The Haploview [23] defined two haplotype blocks in the region of 56,922,109–57,998,003 bp with 17 genes, and Sweep 1.1 defined one haplotype block in the same region, which showed strong LD signals and EHH value downstream of *IGF1*. This could be due to causal effects downstream of *IGF1* or hitchhiking effects due to selection in the *PAH-IGF1* region.

### The chromosome 1 selection signature at the *TRPC4* region

This selection signature was at 176.08–176.29 Mb and was identified by a large standardized AFD value (Z_AFD  = 4.23, Table 3). The EHH analysis identified one haplotype block approximately in the same region in the lean and fat lines, with considerably stronger EHH values in the fat line than in the lean line (Figure S4B). *TRPC4* was at 176,097,670–176,235,464 bp, and therefore this selection signature essentially was due to selection on *TRPC4* in the fat line. This indicates that *TRPC4* is associated with increased AFC. The LD signals were stronger in the fat line than in the lean line (Figure S3C).

### The chromosome 2 selection signature at the *GJD4-CCNY* region

This selection signature was identified by a large AFD value (0.47) and a large Z_AFD value (4.15) in the 12.48–12.80 Mb region with two genes, *GJD4* at 12,756,816–12,760,762 and *CCNY* at 12,774,998–12,818,966 bp (Table 3). The EHH analysis defined a haplotype block at 12,750,042–12,858,095 in the lean line, covering both *GJD4* and *CCNY* (Figure S4C). This region had elevated LD values in the lean line (Figure S3D). These results suggest that both *GJD4* and *CCNY* are associated with selection for low AFC in the lean line.

### The chromosome 4 selection signature at the *NDST4* region

**Figure 6:**
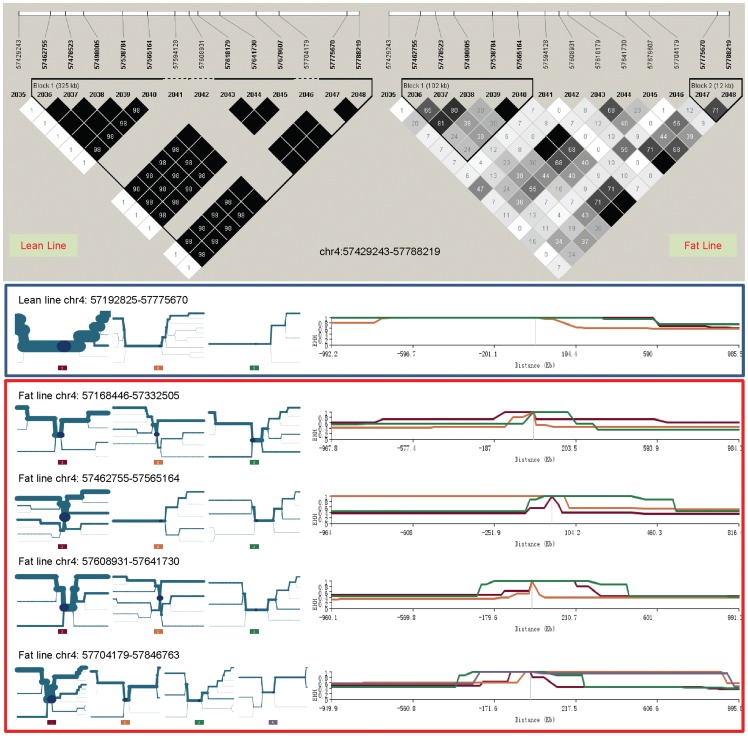
Linkage disequilibrium and extended haplotype heterozygosity in the *NDST4* region of chromosome 4.

The chromosome 4 selection signature at 57.43–57.79 Mb that includes *NDST4* was identified by two measures, the second largest AFD value of 0.50 (along with the chromosome 26 signature) and a large Z_AFD value (4.26). The LD and EEH values strongly suggested that selection in this region was in the lean line only (Figure 6). The *NDST4* gene was at 57,673,334–57,758,645 bp. This gene region had strong LD signals and the highest EHH values in the lean line for over 1 Mb distance (Figure 6), which indicates a strong association of *NDST4* with low AFC. The EHH analysis defined a 0.58 Mb core region at 57,192,825–57,775,670 bp, and EHH values for the main haplotype.

### The chromosome 5 selection signature at the *NOVA1* region

**Figure 7:**
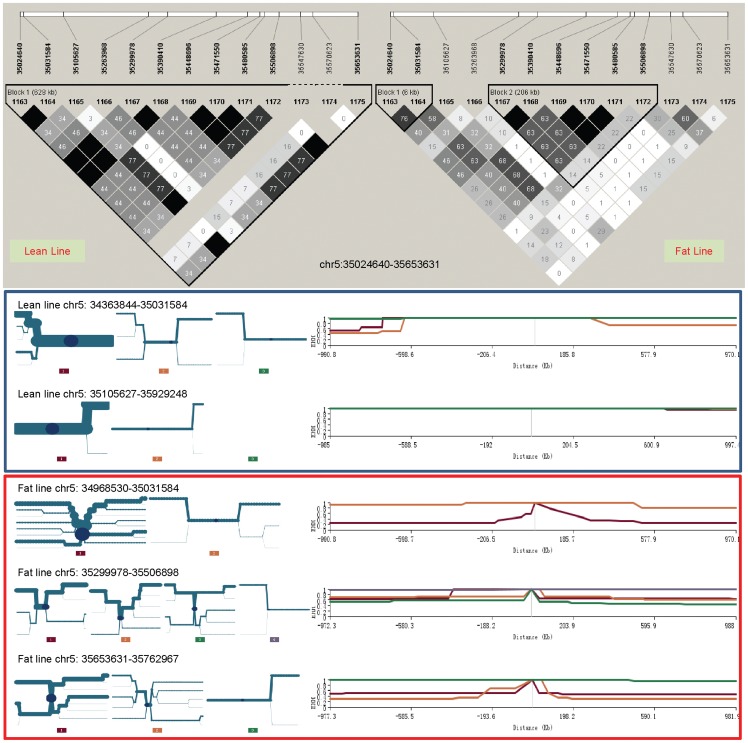
Linkage disequilibrium and extended haplotype heterozygosity in the *NOVA1* region of chromosome 5.

The chromosome 5 selection signature at 35.02–35.65 Mb region that contains *NOVA1* was identified by a Z_AFD value of 4.4. The Z_lean value of −4.07 indicates that selection in this region was mainly in the lean line. The EHH values provided the strongest evidence that selection in this region occurred in the lean line (Figure 7). The two haplotype blocks defined by EHH analysis in the lean line spanned the region of 34,363,844–35,929,248 bp, with the main haploype in each block having a high frequency of 0.68. *NOVA1* at 34,863,690–35,327,411 nearly covered the total length of the two haplotype blocks. This strongly suggests that *NOVA1* was involved in low AFC. The lean line also had more fixed loci and stronger LD than the fat line (Figure 7).

### The chromosome 11 selection signature at the *ESRP2-GALR1* region

The 3.20–3.40 Mb region of chromosome 11 was the only selection signature identified by decreased heterozygosity in the lean line (Z_lean  = −5.80, Table 3). The Z_lean value of −5.80 indicated that selection in this region was in the lean line, and this was confirmed further by the LD and EHH results (Figure S3E, Figure S4D). Five genes were located in this selection signature but none of these genes was known to have a role in fat content. Two genes reported to be associated with human obesity are in the vicinity of this region, *MMP2* [6], [7] which is 0.28 Mb downstream and *FTO* [2]–[5] which is 1.45 Mb downstream of this region.

### The chromosome 15 selection signature at the *GALNT9* region

This selection signature was identified by a high AFD value of 0.44 (Table 3) at the 2.15–2.50 Mb region containing *GALNT9*, which is at 2,393,778–2,485,829 bp. The EHH analysis defined one halplotype block in both lines at 2,263,761–2,470,336 bp, covering 87 kb of the 92 kb *GALNT9* gene (Figure S4E). The main haplotype in the lean line had higher EHH values than the main haplotype in the fat line, and the third most frequent haplotype in the fat line had high EHH values and was likely to have been subjected to selection for high fat content. The lean line had five more fixed loci and stronger LD than the fat line (Figure S3F).

### The chromosome 20 selection signature at the *SYCP2-CADH4* region

The chromosome 20 selection signature at 6.83–7.29 Mb had a peak AFD value of 0.46. This 0.46 Mb region contained six genes. This is a complex region but the long-range AFD and heterozygosity analysis had good agreement with the EHH analysis. Five haplotype blocks in the lean line and four haplotype blocks in the fat line were defined in the EHH analysis. In the lean line, haplotype blocks 1 and 2 covered five genes, *SYCP2*, *PPP1R3D*, *ENSGALG00000024473*, *C20orf177*, and *ENSGALG00000023777*, block 3 covered a blank space, and blocks 4 and 5 covered 109.85 kb of the 414.10 kb *CADH4* at 7,213,069–7,627,166. In the fat line, the first two blocks covered approximately the same region of six genes that was covered by the first two blocks in the lean line, block 3 covered the same blank area covered by block 3 in the lean line, and block 4 covered the same area of *CADH4* covered by the last two blocks in the lean line (Figure S4F). The EHH values in the fat line generally were higher than those in the lean line. The EHH patterns suggest that this region was subjected to selection in both lines. The LD signals in this region were the weakest among the selection signatures identified in this study (Figure S3G). These relatively weak LD signals could be due to the fact that chromosome 20 had considerably more markers per Mb than other chromosomes, e.g., 104.89 SNPs/Mb for this chromosome and 35.51 SNPs/Mb for chromosome 1 (Table 2). Chromosome 20, along with the Z chromosome, had the largest average AFD (0.24) among all chromosomes, excluding the linkage group LGE64 that only had two markers (Table 2).

### The chromosome 26 selection signature at the upstream telomere region

The 0.06–0.29 Mb region of chromosome 26 with 14 genes was the only selection signature identified by increased heterozygosity in the fat line (Z_fat  = 5.02, Table 3), and was the only selection signature identified by another two measures of selection signature, AFD and Z_AFD (Table 3). The peak AFD value (0.50), along with the peak AFD value of the chromosome 4 selection signature, was the second largest of the entire genome, next to the AFD in the *PC1*/*PCSK1* region. This region had strong LD in both lines. The fat line had stronger LD values but the lean line had a larger number of fixed SNP markers (Figure S3H). One haplotype block was defined in each line by EHH analysis. The block size was 55,909–302,113 bp for the lean line and 55,909–334,821 for the fat line (Figure S4G). The EHH values indicated selection in both lines but the fat line had stronger selection than the lean line in this region.

### Haplotype frequencies in selection signatures of the lean and fat lines

Most of the selection signatures had a line-specific main haplotype that either did not exist or had a very low frequency in the other line. In addition to the *PC1*/*PCSK1* region discussed above, selection signatures at *TRPC4*, *GJD4-CCNY*, *NOVA1*, *GALNT9*, *SYCP2-CADH4* and *TULP1-KIF21B* had such line-specific haplotypes (Table S3). For the *TRPC4* region, the main lean line haplotype with a frequency of 0.481 did not exist in the fat line, while the main fat line haplotype with a frequency of 0.462 had a very low frequency of 0.032 in the lean line. This trend was the same for the *GJD4-CCNY*, *NOVA1*, *GALNT9*, and *SYCP2-CADH4* regions. The selection signature at *TULP1-KIF21B* of chromosome 26 had a line-specific main haplotype with a frequency of 0.539 in the fat line, while the main lean line haplotype with a high frequency of 0.808 had a low frequency of 0.253 in the fat line.

For the *PAH-IGF1* region, the main haplotype of the fat line had a high frequency of 0.984 and the same haplotype had a relatively low frequency of 0.392 in the lean line, while the second most frequent (0.329) haplotype in the lean line did not exist in the fat line (Table S3). For the *NDST4* region, the main haplotype in the lean line had a high frequency of 0.742 and a low frequency of 0.16 in the fat line, which did not have a dominant main haplotype (Table S3). For the chromosome 11 signature in *ESRP2-GALR1*, the second most frequent fat line haplotype with a frequency of 0.359 did not exist in the lean line, and the main haplotypes in both lines had a substantial frequency difference, 0.795 in the lean line and 0.529 in the fat line (Table S3). Overall, the data on haplotype frequencies provided strong additional evidence that the regions with selection signatures identified by AFD, heterozygosity change, LD and EHH indeed were subjected to selection.

### Phenotypic effects of selection signatures

Genome-wide association analysis detected a total of 569 SNP markers with phenotypic effects on AFW and AFP and with genome-wide significance (P<10^−6.56^, Figure S1). Of the 569 SNPs, 342 (60%) were significantly associated with both AFW and AFP, and the other 40% were associated with either AFW or AFP. In the literature, 216 quantitative trait loci (QTL) for traits related to abdominal fat in chickens have been reported [35]. Approximately 46% of the 569 SNPs were located in 39% of the 216 reported QTL regions. Nearly all the selection signatures detected in this study had significant SNP effects on AFW and AFP with genome-wide significance, or were in the close proximity to significant SNP effects (Table 3), although most SNP effects in or near the selection signatures were not ranked among the most significant.

**Figure 8:**
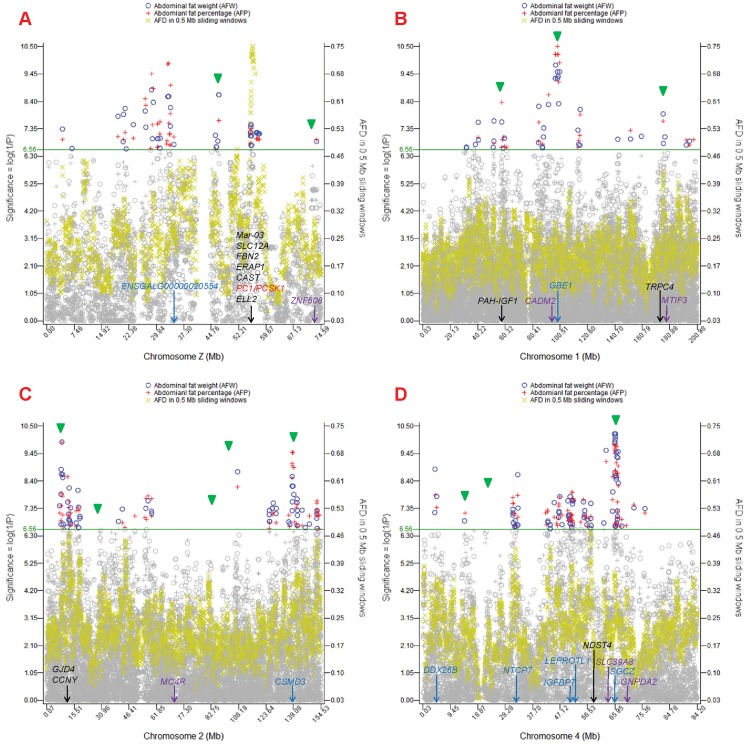
SNP effects on abdominal fat weight (AFW) and abdominal fat percentage (AFP) for four chromosomes. Gene name in black: gene in the selection signature; Gene name in blue: gene in the chromosome region with highly significant SNP effects but not declared as a selection signature; Gene name in purple: gene associated with human obesity [2]–[12]. Blue circles and red plus signs: SNP effects from EPISNP [26], [27]; Green triangles: SNP effects from PLINK [28].

The 55.43–56.16 Mb *PC1*/*PCSK1* region of chromosome Z, which was identified as the most highly selected region of the entire chicken genome had SNP effects on AFW and AFP with genome-wide significance. The rankings of the SNP effects were not among the highest. However, with the knowledge that both haplotype blocks shown in Figure 4 were required to have the highest or lowest AFW and AFP values, the relatively low ranking of the SNP effects could be due to the single-locus analysis, which could detect only half of the phenotypic effects in one haplotype block of the selection signature, thus yielding lower statistical significance. The AFD values overlapped with the SNP effects almost exactly (Figure 8A).

Each selection signature on chromosome 1 (*PAH-IGF1* and *TRPC4* regions) was close to a group of SNP effects, and a human obesity gene, *MTIF3* [2]–[3], was close to the *TRPC4* region (Figure 8B). Selection signatures at the *GJD4-CCNY* region of chromosome 2 (Figure 8C) and at the *GALNT9* region of chromosome 15 (Figure S1) had SNP effects that were ranked high among all chromosomes and were the highest among all selection signatures. The chromosome 5 signature at *NOVA1* had a significant SNP for AFW and AFP, the chromosome 11 selection signature nearly overlapped with SNP effects that were close to *MMP2*, a human obesity gene [6], [7], and the chromosome 26 signature had a cluster of SNP effects with similarly low significance (Figure S1). The chromosome 20 signature had a large number of significant SNP effects for AFW and AFP, with *CADH4* having one of the most significant SNP for AFW and AFP and five other significant SNPs for AFW. The SNP effects upstream of *CADH4* were in an intergenic region. The peak AFD values and significant SNP effects overlapped well, similar to the overlap between AFD and SNP effects in the *PC1*/*PCSK1* region (Figure S1).

The chromosome 4 signature at *NDST4* had one significant SNP for AFW and AFP that was ranked low, and was between two large groups of SNP effects (Figure 8D). On the left side of the *NDST4* region was a large group of SNP effects in an 8.68 Mb region at 42.95–51.63 Mb. Near the downstream end of this large 8.68 Mb region was *LEPROTL1* (a leptin receptor) at 50,714,780–50,717,850 bp. This molecule is on the leptin/melanocortin pathway, which is related to obesity [25]. This large (8.68 Mb) region had three locations with large AFD values, the left end, the right end and the *IGFBP7-LEPROTL1* region. The *SGCZ* region downstream of the *NDST4* selection signature had the most significant SNP effect and substantial AFD (0.35).

### Candidate genes for chicken AFC

The most apparent candidate gene was *PC1*/*PCSK1* in the most significant 0.73 Mb region at 55.43–56.16 Mb of chromosome Z. This region clearly stood out as the region with the strongest evidence of selection in the entire chicken genome, in both the lean and fat lines in this study. In addition, the known function of *PCSK1* in regulating insulin biosynthesis and its known association with human obesity [8]–[12] make *PC1*/*PCSK1* the most likely candidate gene to have a causal effect on AFC. However, haplotype analysis showed that *PC1*/*PCSK1* should not be the only gene with causal effect in this region, and that the evidence in this region was consistent with a two-locus model with two causal loci flanking the recombination point at 55,736,592–55,808,443 bp inside *FBN2*.

Upstream of the recombination point were part of *Mar-03*, *SLC12A2*, and the upstream two-thirds of *FBN2.* None of these three genes has known biological functions specifically related to fat metabolism. However, based on chromosome positions, *Mar-03* was on the left end of this region and should be least likely to have causal effects, because a substantial hitchhiking effect upstream of *Mar-03* should have been observed if *Mar-03* had been one of the most significant genes. This analysis leaves *SLC12A2* and the upstream two-thirds of *FBN2* to be the candidate genes for causal effects. Downstream of the recombination point were the downstream one-third of *FBN*, *ERAP1*, *CAST*, *PC1*/*PCSK1* and part of *ELL2*. In this block, *PC1*/*PCSK1* is the most apparent candidate gene but the downstream one-third of *FBN*, *ERAP1*, *CAST* and *ELL2* could not be excluded from having causal effects. However, *ELL2* is unlikely to be the target of selection given that AFD values downstream of *ELL2* dropped rather sharply (Figure 3).

Selection signatures that occur in single genes should make those genes apparent targets for candidate genes, such as *PAH-IGF1*, *TRPC4*, *GJD4*, *CCNY, NDST, NOVA1* and *GALNT9*. The chromosome 20 signature at 6.83–7.29 Mb involved five genes with *CADH4* being the largest gene of that region. The chromosome 11 signature of the *ESRP2-GALR1* region at 3.20–3.40 Mb had five genes without known biological functions relevant to AFC. However, a human obesity gene (*MMP2*) was only 0.28 Mb downstream at 3.68–3.72 Mb, and a second human obesity gene (*FTO*) was less than 1.45 Mb downstream at 4.85–4.89 Mb, making this region an interesting region for candidate genes. Within this region, the most significant SNP was at 4.40 Mb for AFW and AFP (Figure S1), 0.68 Mb downstream of *MMP2* and 0.45 Mb upstream of *FTO*, indicating that the region of *MMP2-FTO* could contain a causal effect. The chromosome 26 selection signature at 0.06–0.29 Mb involved about 14 genes. In this region, the 0.14–0.27 region had five significant SNPs for AFW and AFP, with one SNP in *TBC1D22B* and two SNPs in *CAC1S*. The upstream telomere region of chromosome 26 is a gene-dense region, and current evidence would not pinpoint with good accuracy to a causal location in this region. The general conclusion for this region is that a causal effect should exist in the 0–0.5 Mb region of chromosome 26.

In summary, ten selection signatures on chromosomes 1, 2, 4, 5, 11, 15, 20, 26 and Z were identified in the current study. The 0.73 Mb *PC1/PCSK1* region of the Z chromosome at 55.43–56.16 Mb was the most significant selected region. The *PC1/PCSK1* gene in this region might be important for chicken abdominal fat content.

## Supporting Information

Figure S1 **SNP effects on abdominal fat weight (AFW) and abdominal fat percentage (AFP).** Blue circles and red plus signs: SNP effects from EPISNP [26], [27]; Green triangles: SNP effects from PLINK [28].
(PDF)

Figure S2 **AFD and Z values in 0.5 Mb sliding windows of SNP markers.**
(PDF)

Figure S3 **Linkage disequilibrium patterns of the selection signatures.**
(PDF)

Figure S4 **Extended haplotype homozygosity in selection signatures.**
(PDF)

Table S1 **Full gene names of candidate genes for chicken abdominal fat content.** The genes in Table 3 and genes in black and blue colors in Figure S1 are included. Human obesity genes are not included, except PC1/PCSK1.
(DOC)

Table S2 **Human obesity genes on the chicken genome.**
(DOC)

Table S3 **Haplotype frequencies in selection signatures.**
(DOC)
