# Supplementary material for: Selection Signature Analysis Implicates the PC1/PCSK1 Region for Chicken Abdominal Fat Content
Source: PLoS One. 2012 Jul 11;7(7):e40736. doi: 10.1371/journal.pone.0040736 (PMC3394724; doi:10.1371/journal.pone.0040736)
Supplement: Figure S1 — SNP effects on abdominal fat weight (AFW) and abdominal fat percentage (AFP). Blue circles and red plus signs: SNP effects from EPISNP [26], [27]; Green triangles: SNP effects from PLINK [28]. (PDF) [file pone.0040736.s001.pdf]

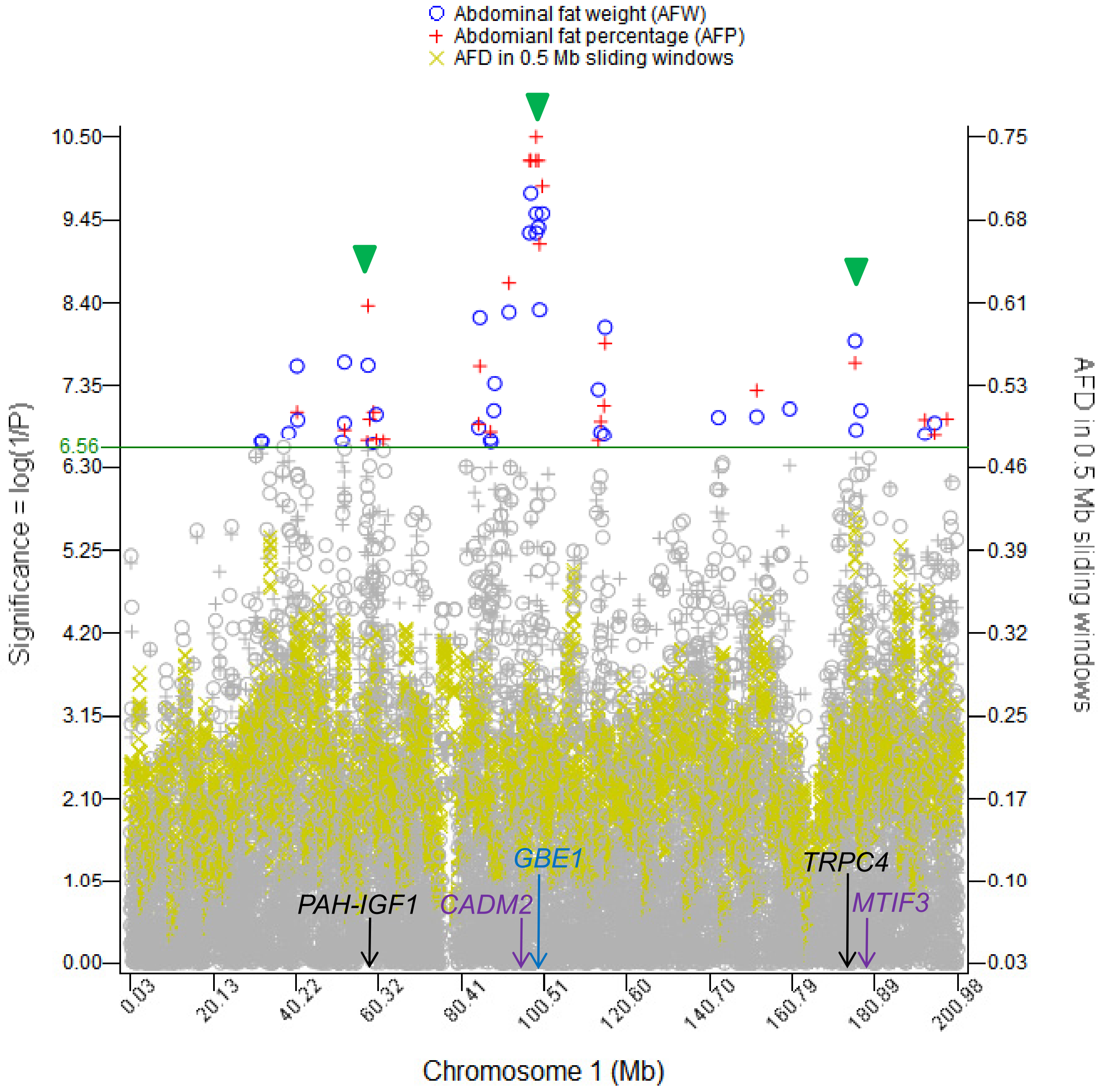

Abdominal fat weight (AFW)
Abdomianl fat percentage (AFP)
AFD in 0.5 Mb sliding windows
Significance = log(1/P)
AFD in 0.5 Mb sliding windows
10.50
9.45
8.40
7.35
6.56
6.30
5.25
4.20
3.15
2.10
1.05
0.00
0.75
0.68
0.61
0.53
0.46
0.39
0.32
0.25
0.17
0.10
0.03
PAH-IGF1
CADM2
GBE1
TRPC4
MTIF3
0.03
20.13
40.22
60.32
80.41
100.51
120.60
140.70
160.79
180.89
200.98
Chromosome 1 (Mb)

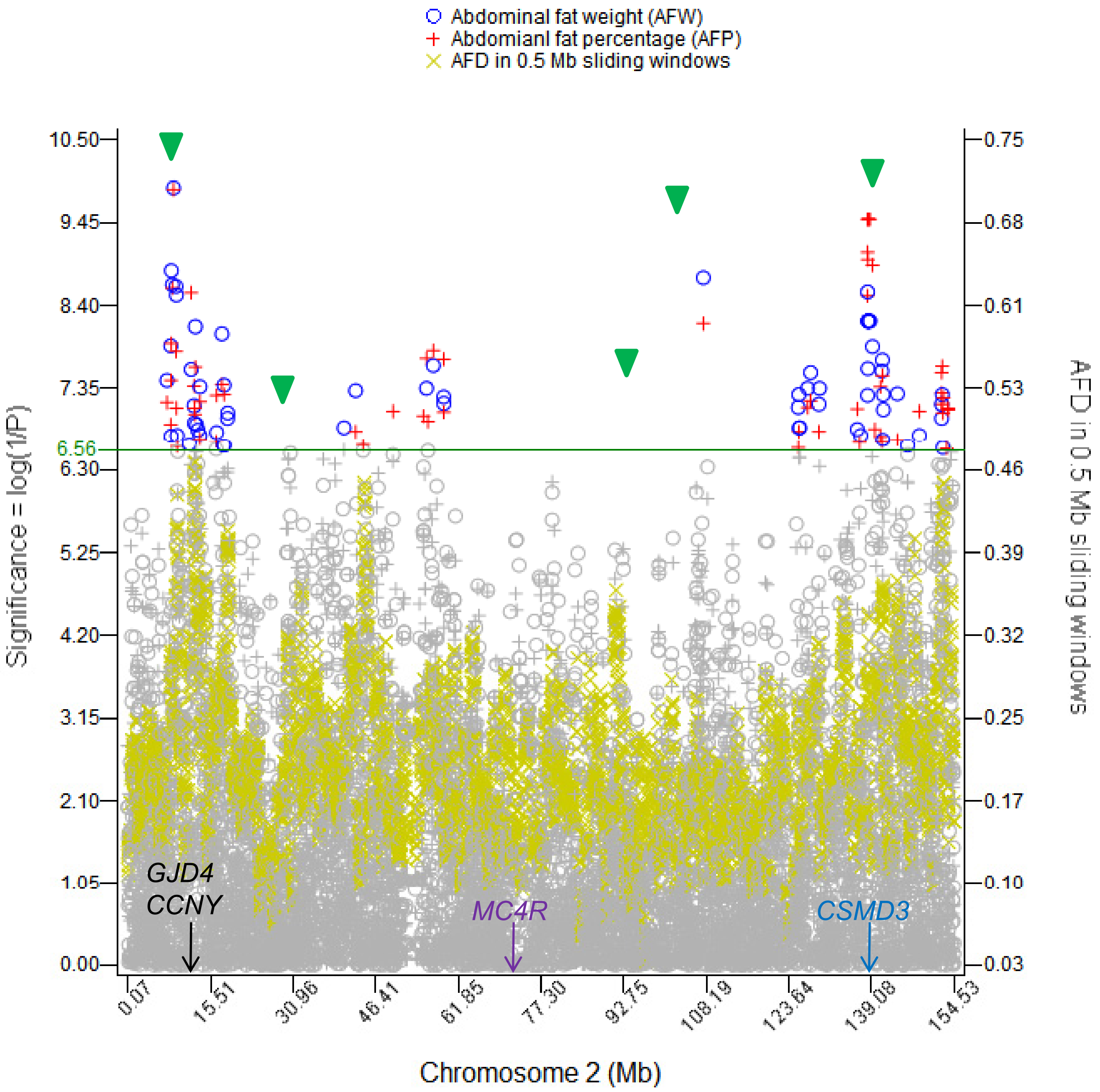

Abdominal fat weight (AFW)
Abdomianl fat percentage (AFP)
AFD in 0.5 Mb sliding windows
Significance = log(1/P)
AFD in 0.5 Mb sliding windows
10.50
9.45
8.40
7.35
6.56
6.30
5.25
4.20
3.15
2.10
1.05
0.00
0.75
0.68
0.61
0.53
0.46
0.39
0.32
0.25
0.17
0.10
0.03
GJD4
CCNY
MC4R
CSMD3
0.07
15.51
30.96
46.41
61.85
77.30
92.75
108.19
123.64
139.08
154.53
Chromosome 2 (Mb)

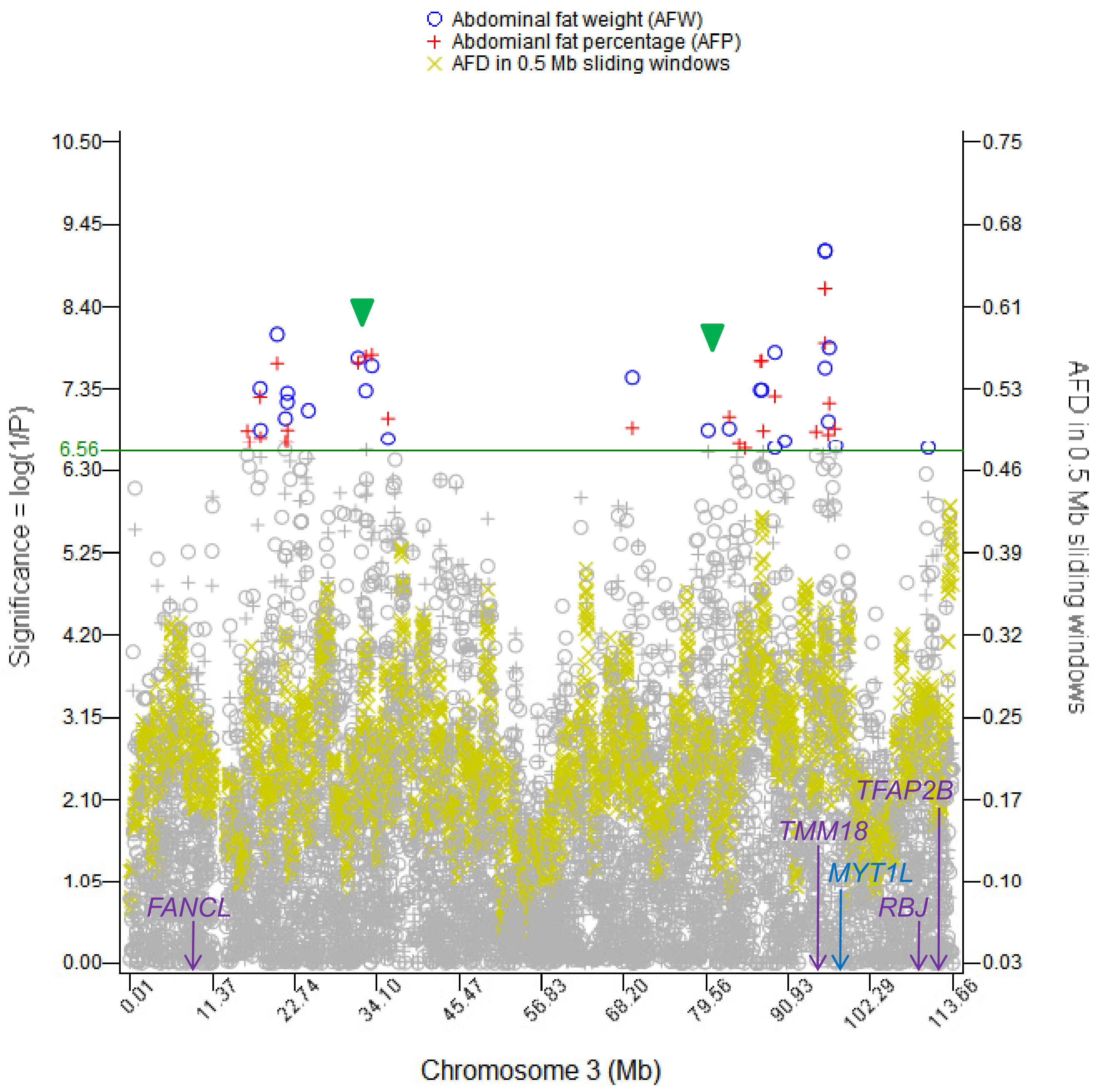

Abdominal fat weight (AFW)
Abdomianl fat percentage (AFP)
AFD in 0.5 Mb sliding windows
Significance = log(1/P)
AFD in 0.5 Mb sliding windows
Chromosome 3 (Mb)
10.50
9.45
8.40
7.35
6.56
6.30
5.25
4.20
3.15
2.10
1.05
0.00
0.75
0.68
0.61
0.53
0.46
0.39
0.32
0.25
0.17
0.10
0.03
0.01
11.37
22.74
34.10
45.47
56.83
68.20
79.56
90.93
102.29
113.66
FANCL
TMM18
MYT1L
RBJ
TFAP2B

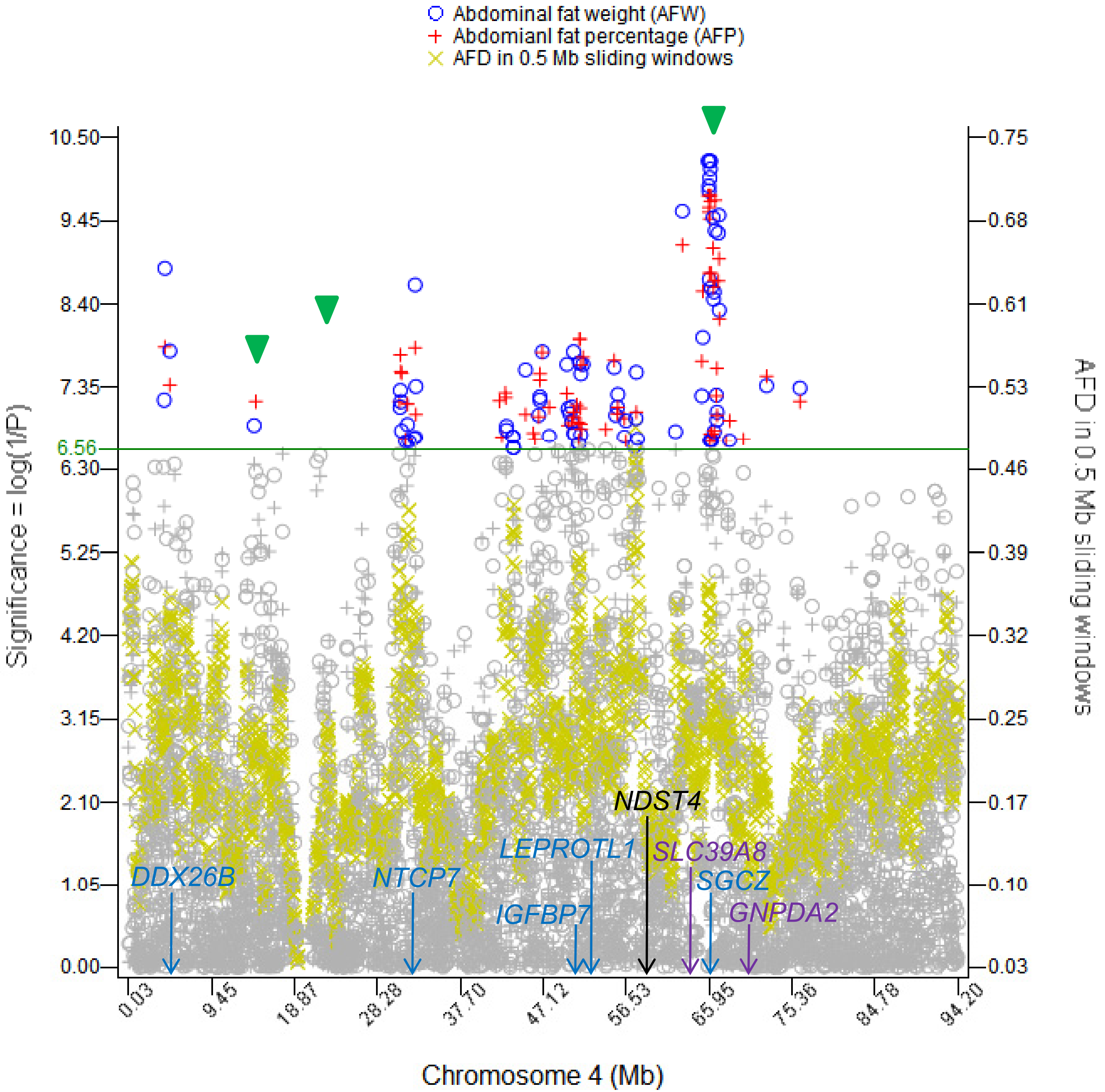

Abdominal fat weight (AFW)
Abdomianl fat percentage (AFP)
AFD in 0.5 Mb sliding windows
Significance = log(1/P)
10.50
9.45
8.40
7.35
6.56
6.30
5.25
4.20
3.15
2.10
1.05
0.00
AFD in 0.5 Mb sliding windows
0.75
0.68
0.61
0.53
0.46
0.39
0.32
0.25
0.17
0.10
0.03
DDX26B
NTCP7
LEPROTL1
IGFBP7
NDST4
SLC39A8
SGCZ
GNPDA2
0.03
9.45
18.87
28.28
37.70
47.12
56.53
65.95
75.36
84.78
94.20
Chromosome 4 (Mb)

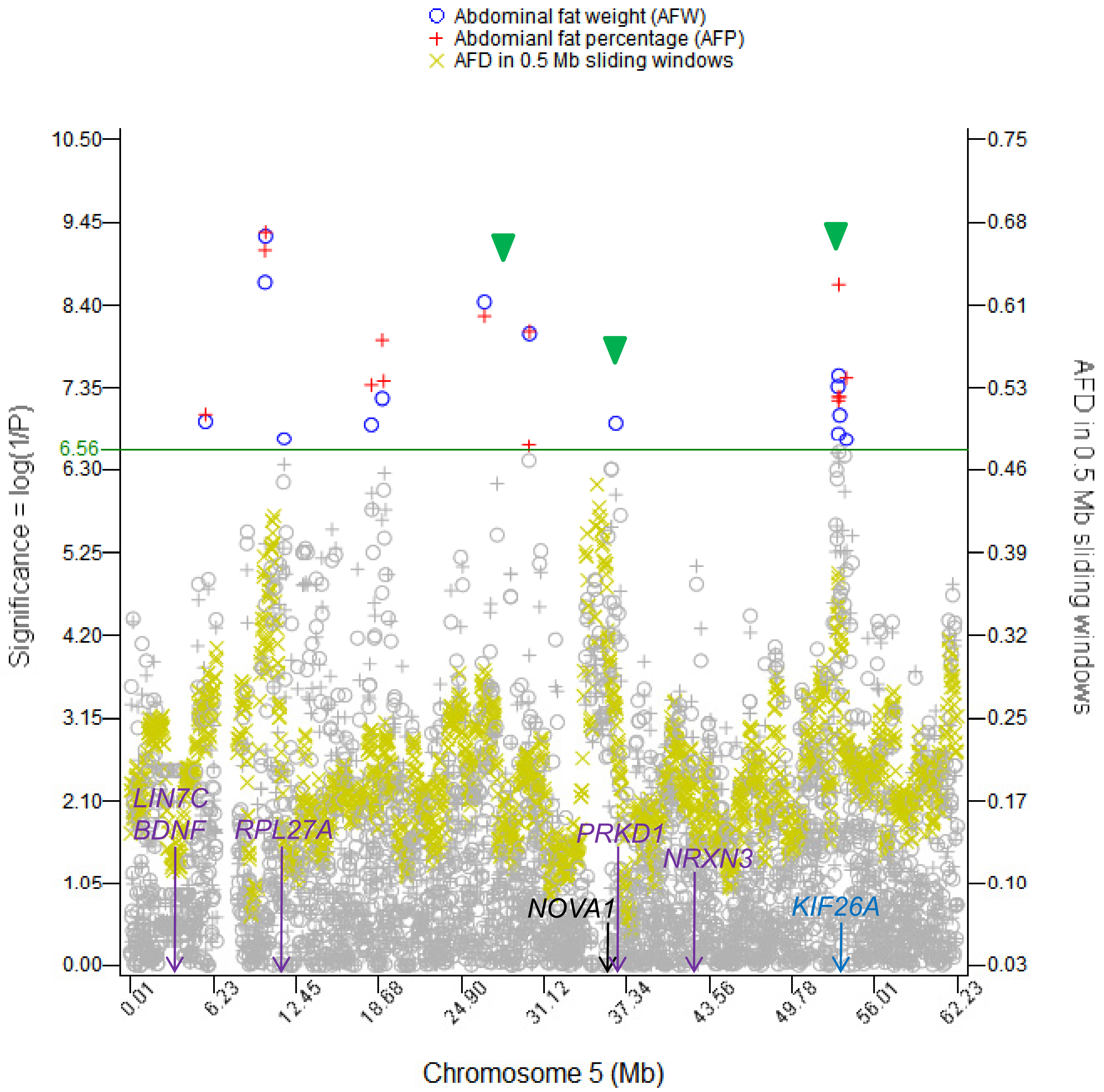

Abdominal fat weight (AFW)
Abdomianl fat percentage (AFP)
AFD in 0.5 Mb sliding windows
Significance = log(1/P)
10.50
9.45
8.40
7.35
6.56
6.30
5.25
4.20
3.15
2.10
1.05
0.00
0.75
0.68
0.61
0.53
0.46
0.39
0.32
0.25
0.17
0.10
0.03
AFD in 0.5 Mb sliding windows
LIN7C
BDNF
RPL27A
PRKD1
NRXN3
NOVA1
KIF26A
0.01
6.23
12.45
18.68
24.90
31.12
37.34
43.56
49.78
56.01
62.23
Chromosome 5 (Mb)

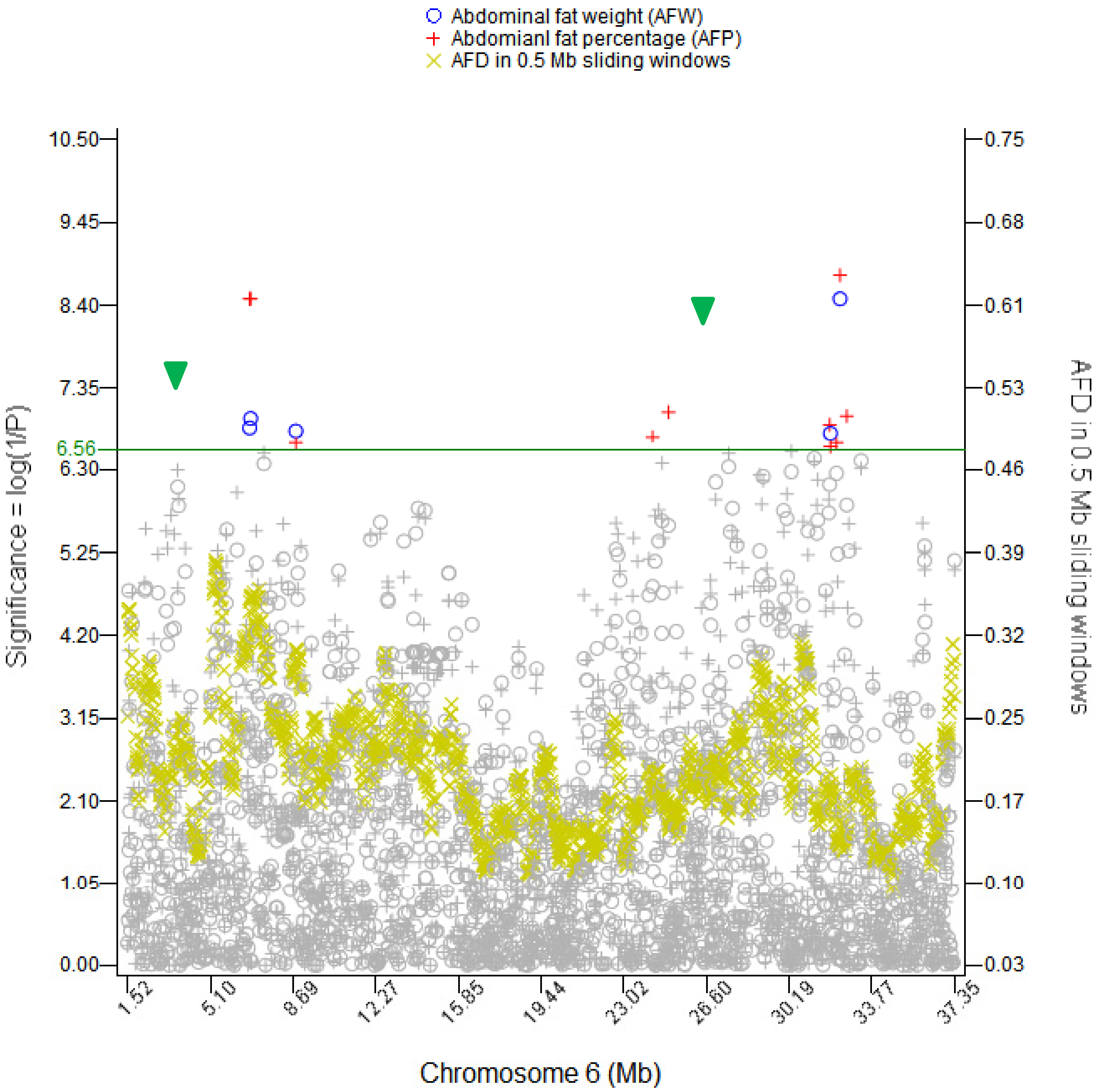

Abdominal fat weight (AFW)
Abdomianl fat percentage (AFP)
AFD in 0.5 Mb sliding windows
Significance = log(1/P)
10.50
9.45
8.40
7.35
6.56
6.30
5.25
4.20
3.15
2.10
1.05
0.00
AFD in 0.5 Mb sliding windows
0.75
0.68
0.61
0.53
0.46
0.39
0.32
0.25
0.17
0.10
0.03
1.52
5.10
8.69
12.27
15.85
19.44
23.02
26.60
30.19
33.77
37.35
Chromosome 6 (Mb)

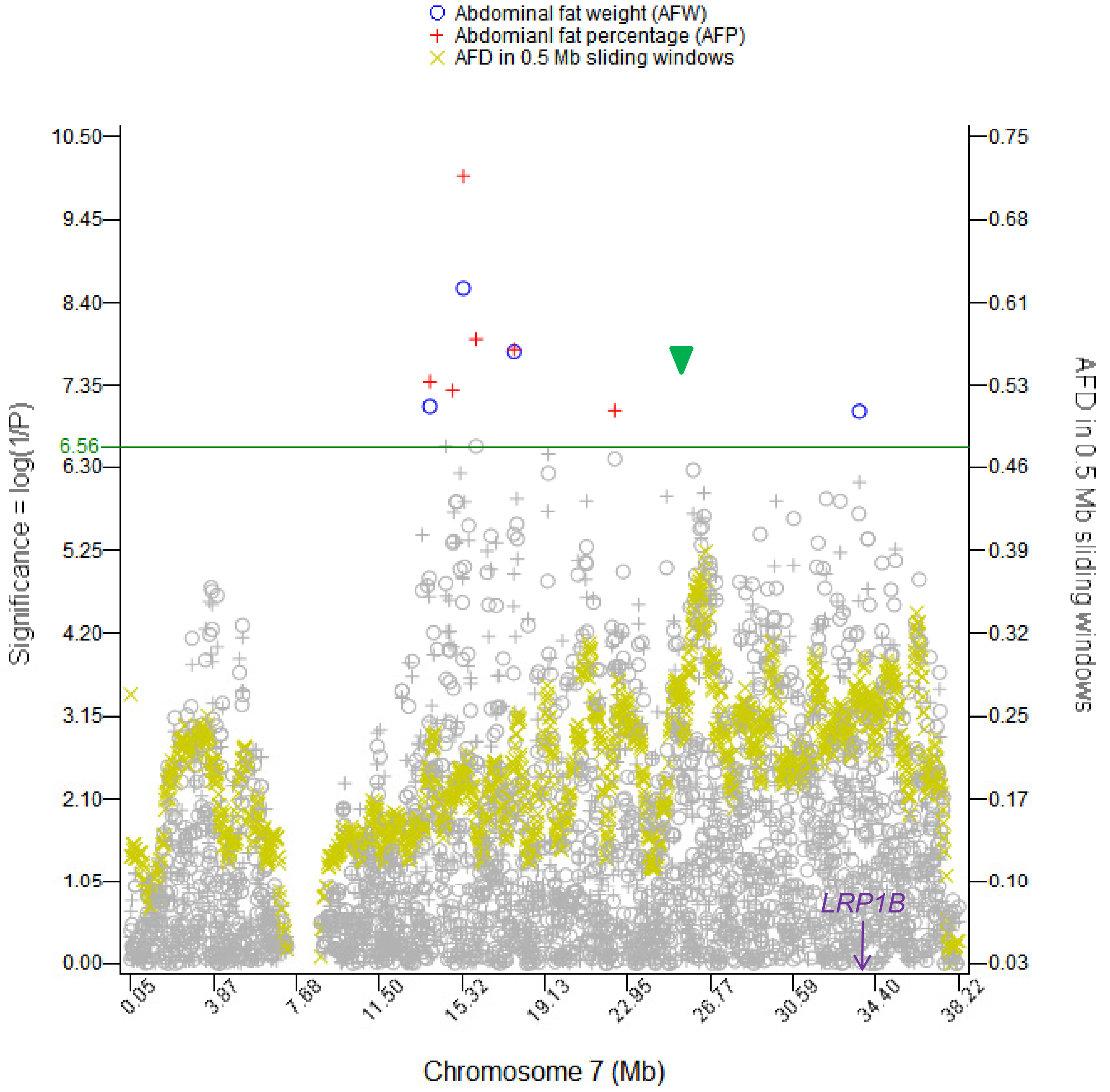
Abdominal fat weight (AFW)
Abdomianl fat percentage (AFP)
AFD in 0.5 Mb sliding windows
Significance = log(1/P)
AFD in 0.5 Mb sliding windows
10.50
9.45
8.40
7.35
6.56
6.30
5.25
4.20
3.15
2.10
1.05
0.00
0.75
0.68
0.61
0.53
0.46
0.39
0.32
0.25
0.17
0.10
0.03
0.05
3.87
7.68
11.50
15.32
19.13
22.95
26.77
30.59
34.40
38.22
LRP1B
Chromosome 7 (Mb)

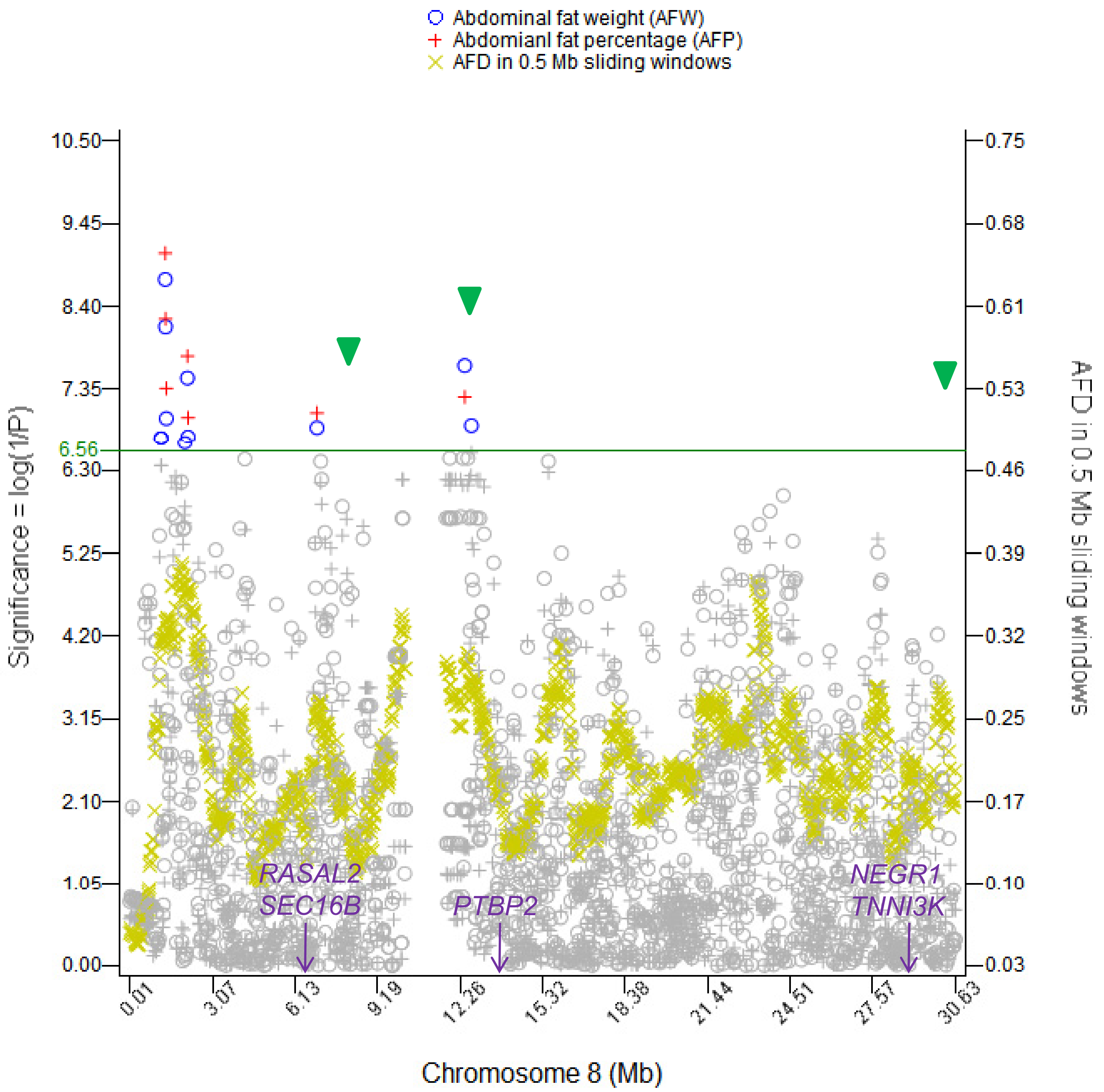

Abdominal fat weight (AFW)
Abdomianl fat percentage (AFP)
AFD in 0.5 Mb sliding windows
Significance = log(1/P)
AFD in 0.5 Mb sliding windows
10.50
9.45
8.40
7.35
6.56
6.30
5.25
4.20
3.15
2.10
1.05
0.00
0.75
0.68
0.61
0.53
0.46
0.39
0.32
0.25
0.17
0.10
0.03
RASAL2
SEC16B
PTBP2
NEGR1
TNNI3K
0.01
3.07
6.13
9.19
12.26
15.32
18.38
21.44
24.51
27.57
30.63
Chromosome 8 (Mb)

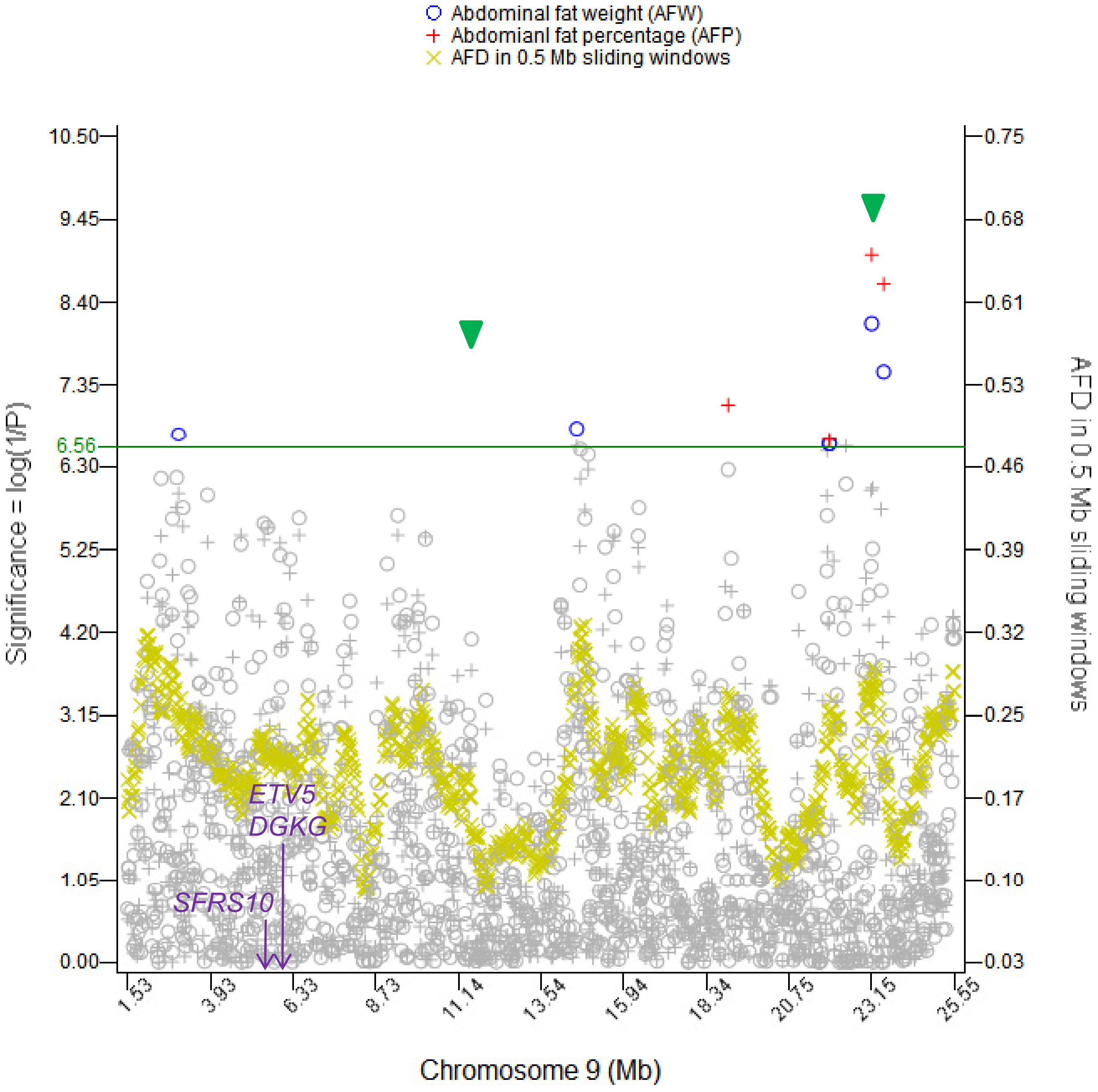

Abdominal fat weight (AFW)
Abdomianl fat percentage (AFP)
AFD in 0.5 Mb sliding windows
Significance = log(1/P)
10.50
9.45
8.40
7.35
6.56
6.30
5.25
4.20
3.15
2.10
1.05
0.00
AFD in 0.5 Mb sliding windows
0.75
0.68
0.61
0.53
0.46
0.39
0.32
0.25
0.17
0.10
0.03
ETV5
DGKG
SFRS10
1.53
3.93
6.33
8.73
11.14
13.54
15.94
18.34
20.75
23.15
25.55
Chromosome 9 (Mb)

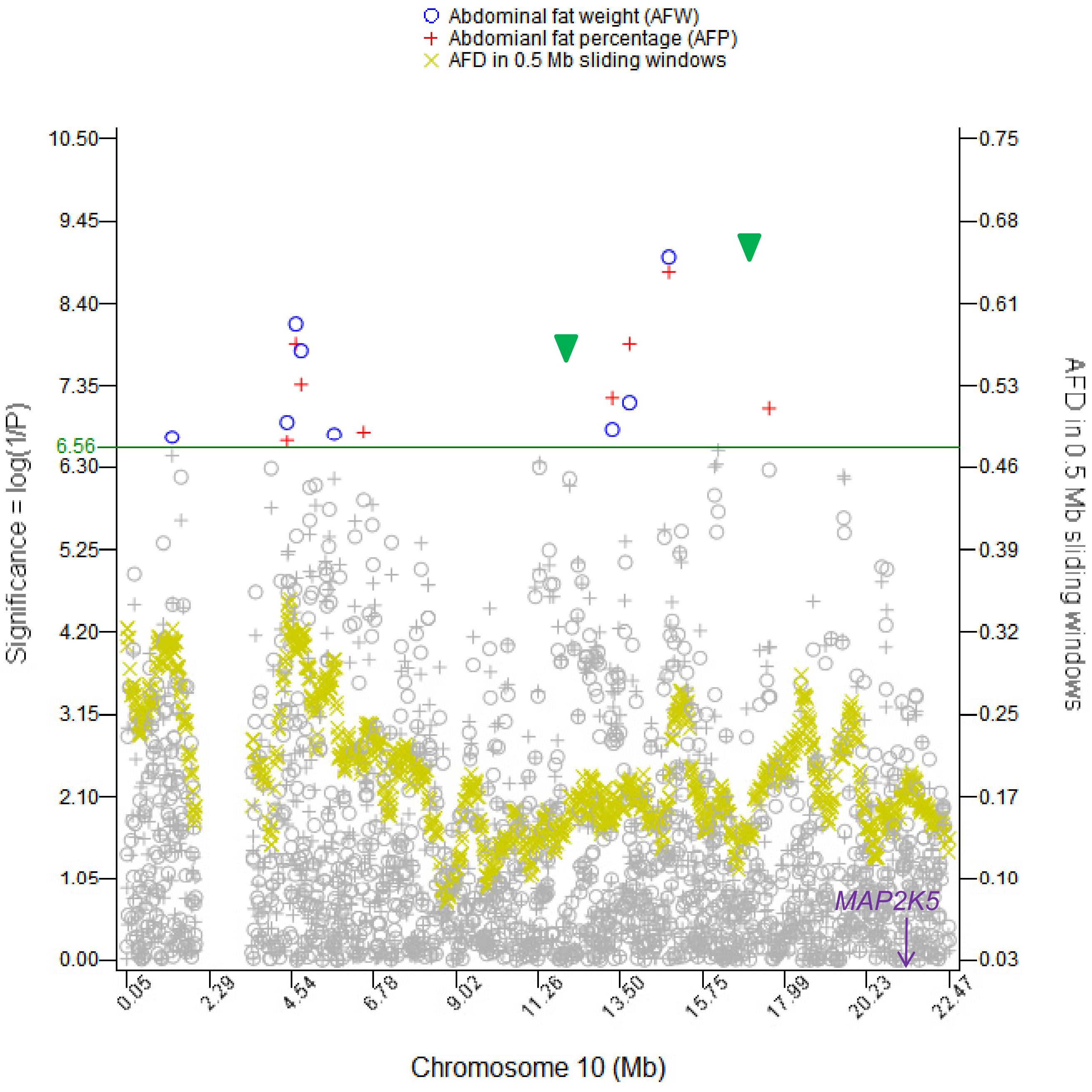

Abdominal fat weight (AFW)
Abdomianl fat percentage (AFP)
AFD in 0.5 Mb sliding windows
Significance = log(1/P)
10.50
9.45
8.40
7.35
6.56
6.30
5.25
4.20
3.15
2.10
1.05
0.00
AFD in 0.5 Mb sliding windows
0.75
0.68
0.61
0.53
0.46
0.39
0.32
0.25
0.17
0.10
0.03
MAP2K5
0.05
2.29
4.54
6.78
9.02
11.26
13.50
15.75
17.99
20.23
22.47
Chromosome 10 (Mb)

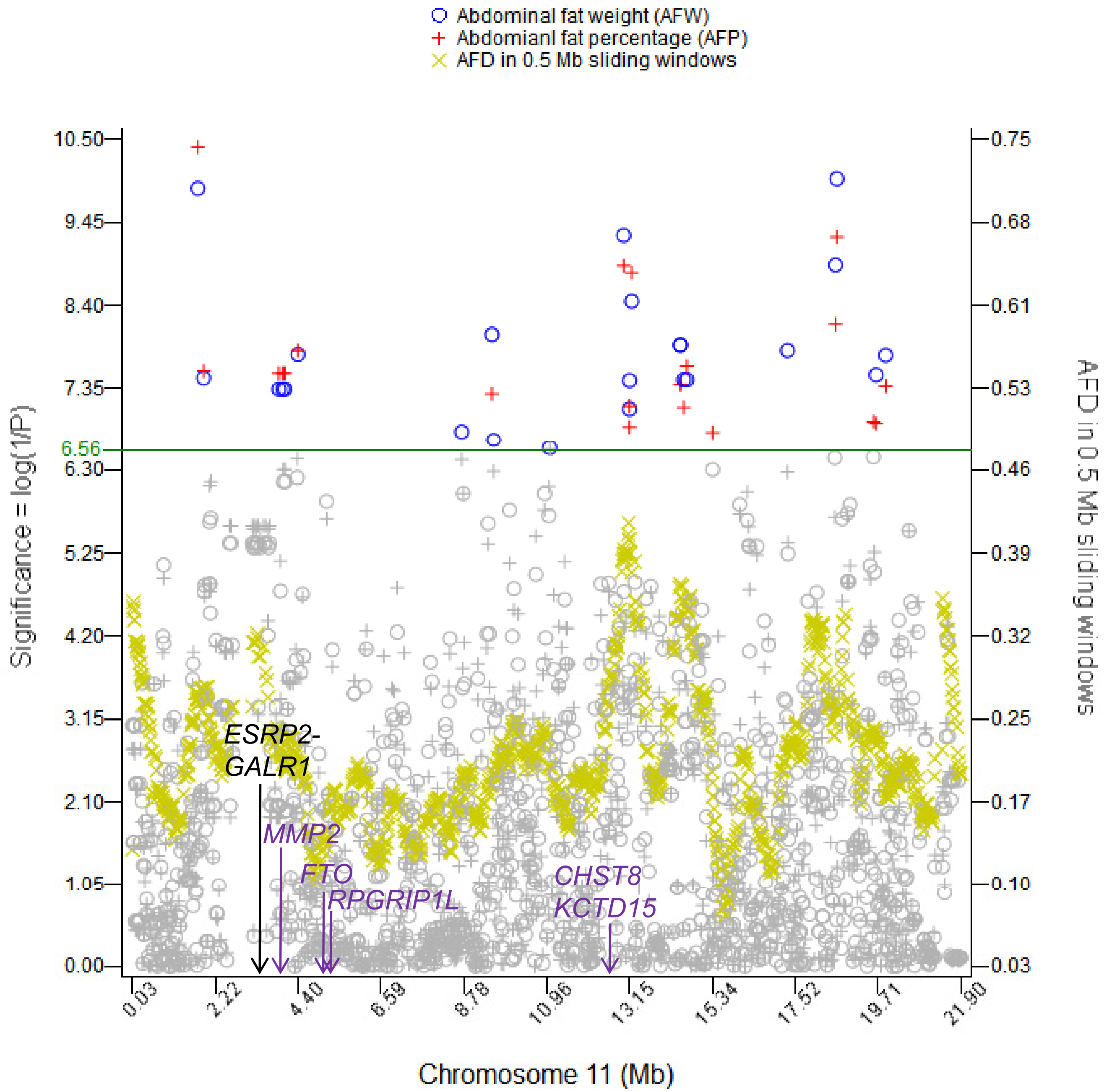

Abdominal fat weight (AFW)
Abdomianl fat percentage (AFP)
AFD in 0.5 Mb sliding windows
Significance = log(1/P)
AFD in 0.5 Mb sliding windows
10.50
9.45
8.40
7.35
6.56
6.30
5.25
4.20
3.15
2.10
1.05
0.00
0.75
0.68
0.61
0.53
0.46
0.39
0.32
0.25
0.17
0.10
0.03
ESRP2-
GALR1
MMP2
FTO
RPGRIP1L
CHST8
KCTD15
0.03
2.22
4.40
6.59
8.78
10.96
13.15
15.34
17.52
19.71
21.90
Chromosome 11 (Mb)

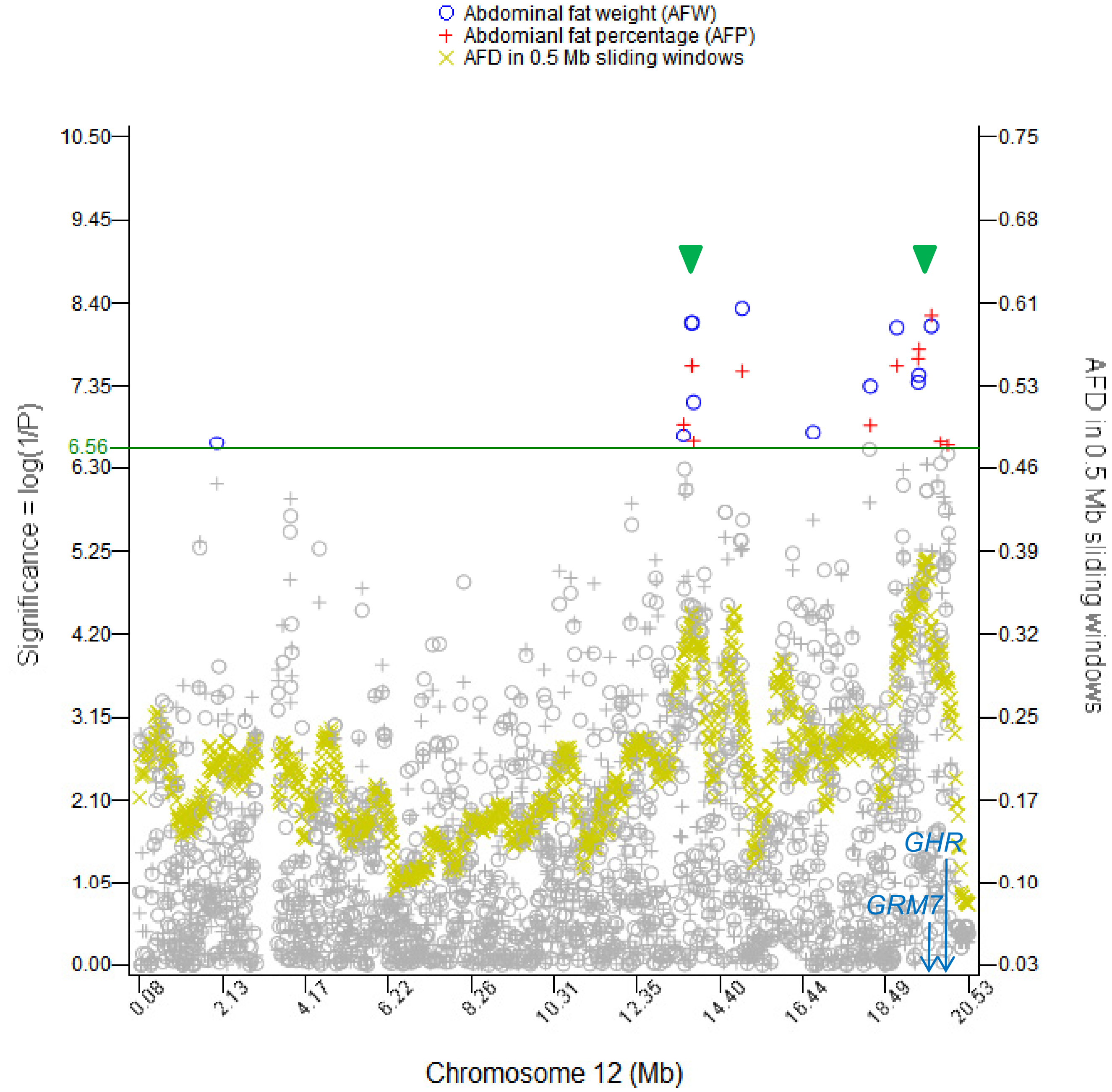

Abdominal fat weight (AFW)
Abdomianl fat percentage (AFP)
AFD in 0.5 Mb sliding windows
Significance = log(1/P)
AFD in 0.5 Mb sliding windows
Chromosome 12 (Mb)
10.50
9.45
8.40
7.35
6.56
6.30
5.25
4.20
3.15
2.10
1.05
0.00
0.75
0.68
0.61
0.53
0.46
0.39
0.32
0.25
0.17
0.10
0.03
0.08
2.13
4.17
6.22
8.26
10.31
12.35
14.40
16.44
18.49
20.53
GHR
GRM7

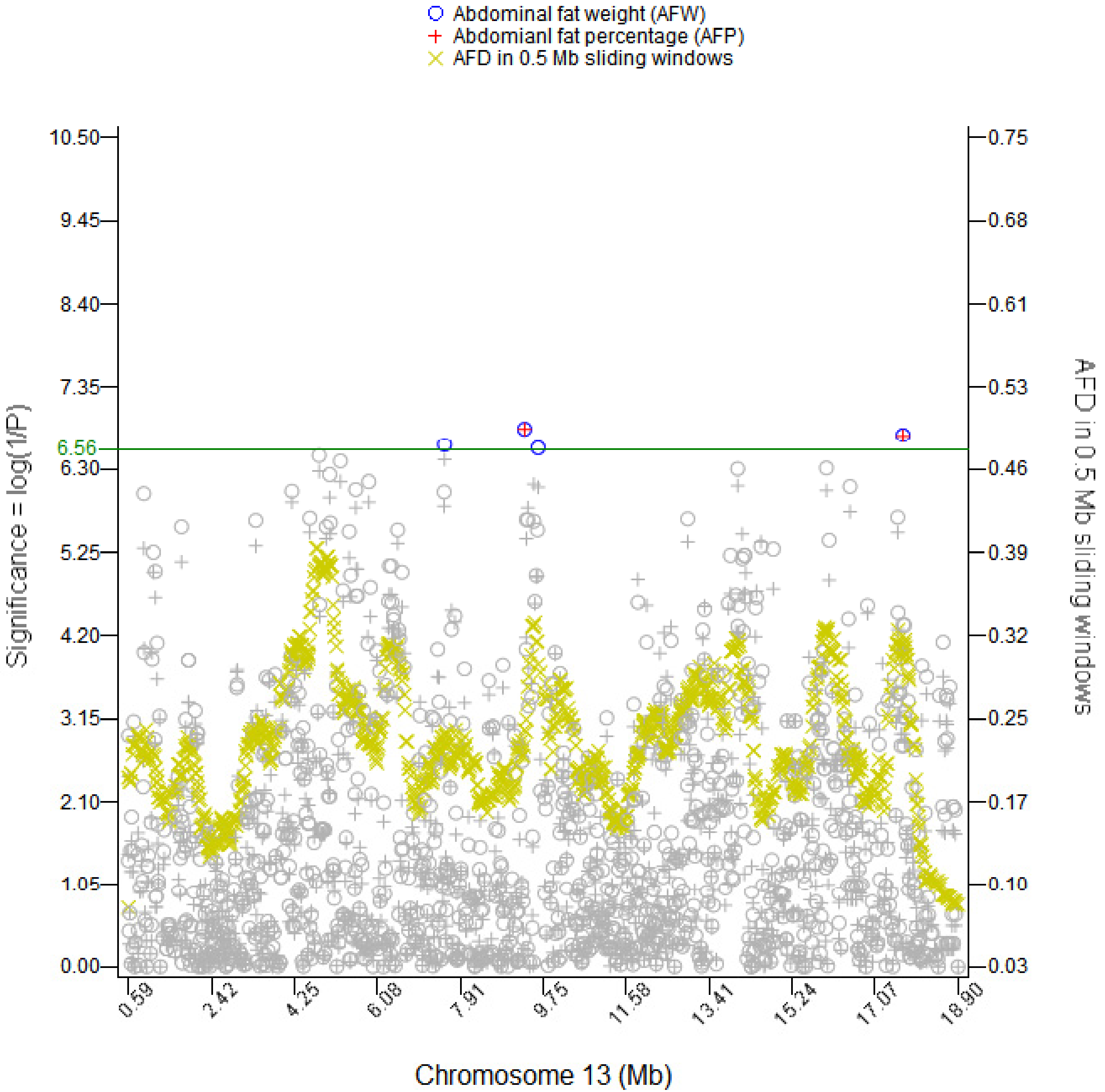

Abdominal fat weight (AFW)
Abdomianl fat percentage (AFP)
AFD in 0.5 Mb sliding windows
10.50
9.45
8.40
7.35
6.56
6.30
5.25
4.20
3.15
2.10
1.05
0.00
0.75
0.68
0.61
0.53
0.46
0.39
0.32
0.25
0.17
0.10
0.03
Significance = log(1/P)
AFD in 0.5 Mb sliding windows
0.59
2.42
4.25
6.08
7.91
9.75
11.58
13.41
15.24
17.07
18.90
Chromosome 13 (Mb)

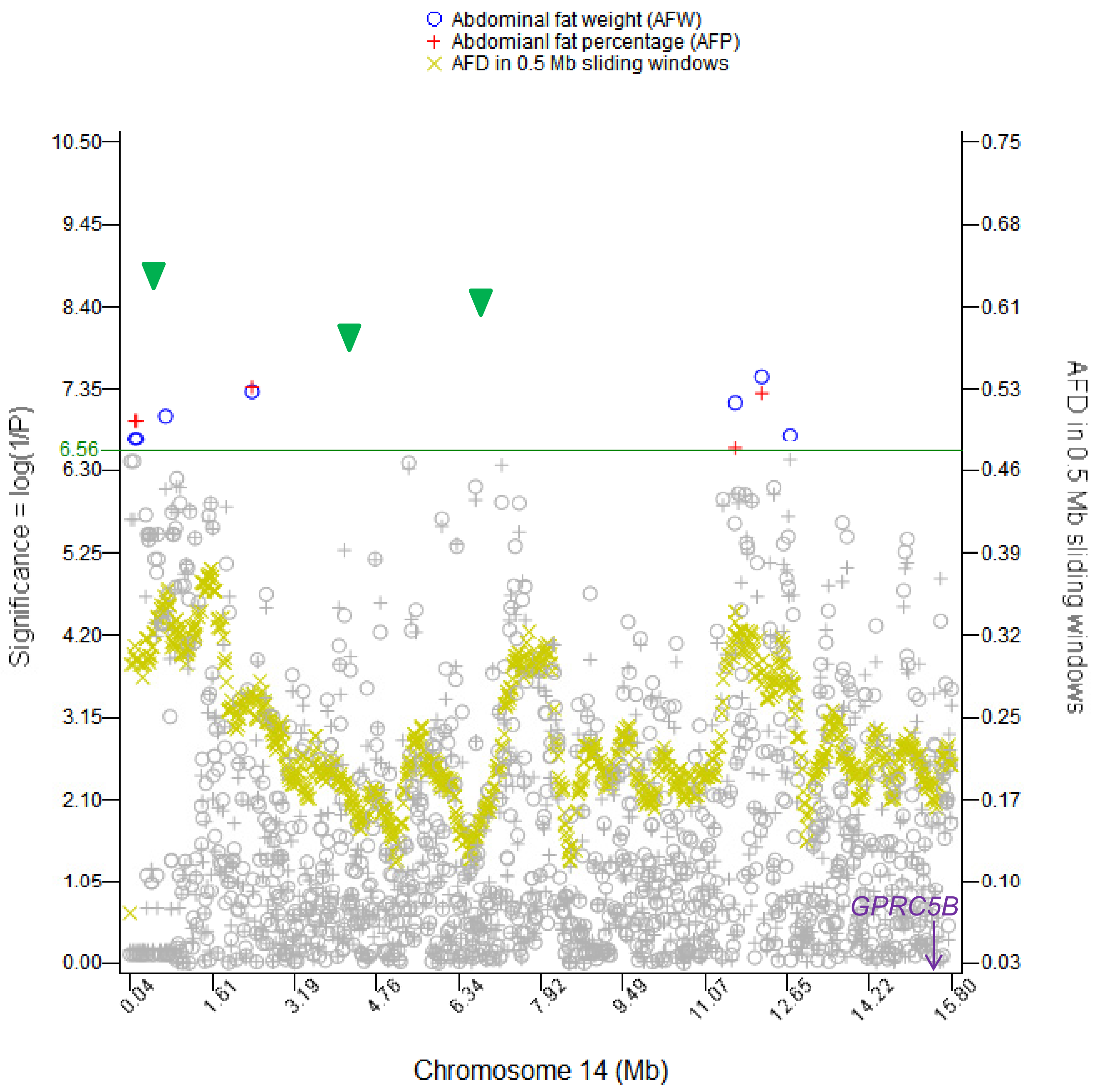

Abdominal fat weight (AFW)
Abdomianl fat percentage (AFP)
AFD in 0.5 Mb sliding windows
Significance = log(1/P)
AFD in 0.5 Mb sliding windows
6.56
GPRC5B
Chromosome 14 (Mb)

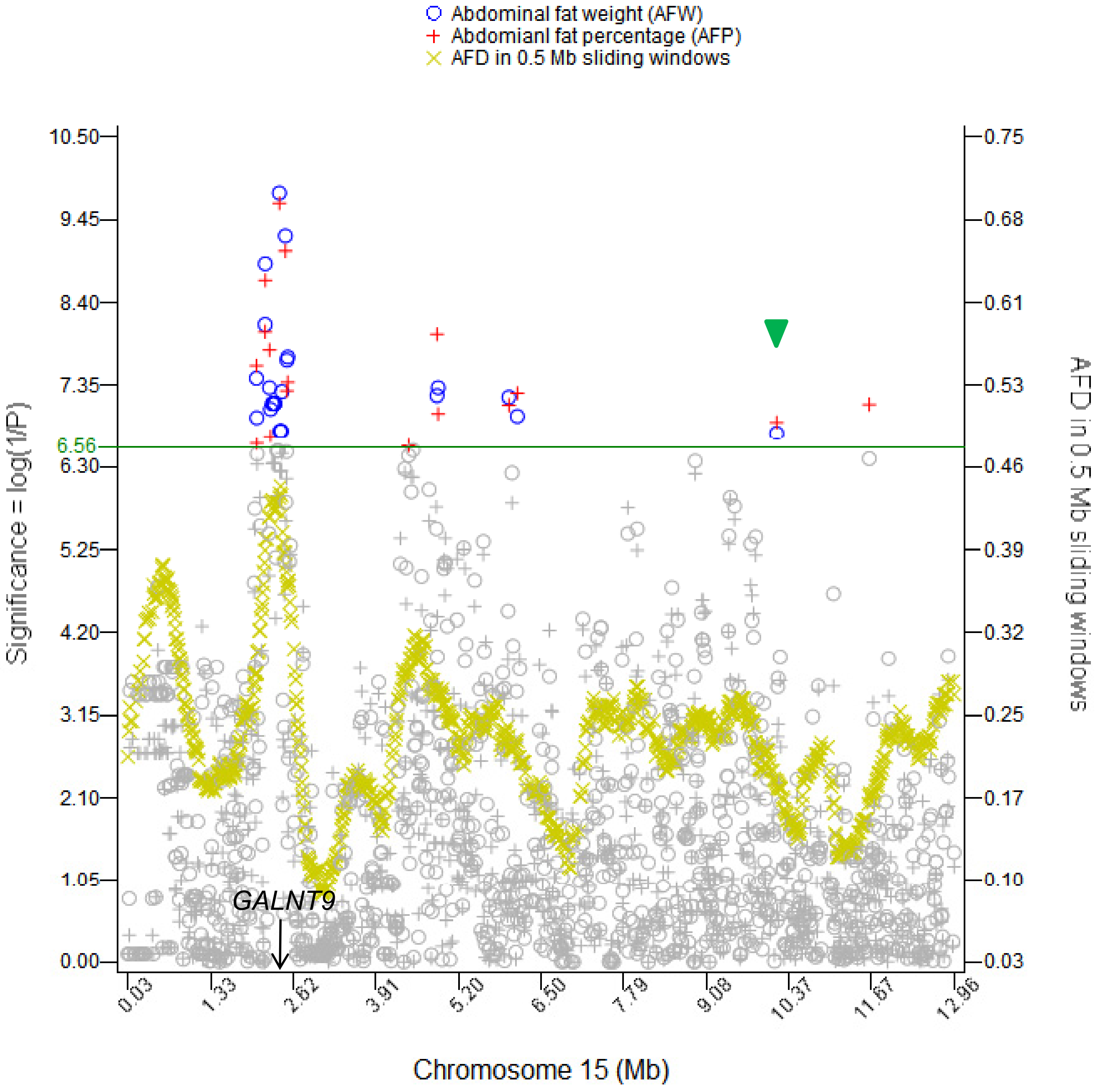
Abdominal fat weight (AFW)
Abdomianl fat percentage (AFP)
AFD in 0.5 Mb sliding windows
Significance = log(1/P)
10.50
9.45
8.40
7.35
6.56
6.30
5.25
4.20
3.15
2.10
1.05
0.00
AFD in 0.5 Mb sliding windows
0.75
0.68
0.61
0.53
0.46
0.39
0.32
0.25
0.17
0.10
0.03
GALNT9
0.03
1.33
2.62
3.91
5.20
6.50
7.79
9.08
10.37
11.67
12.96
Chromosome 15 (Mb)

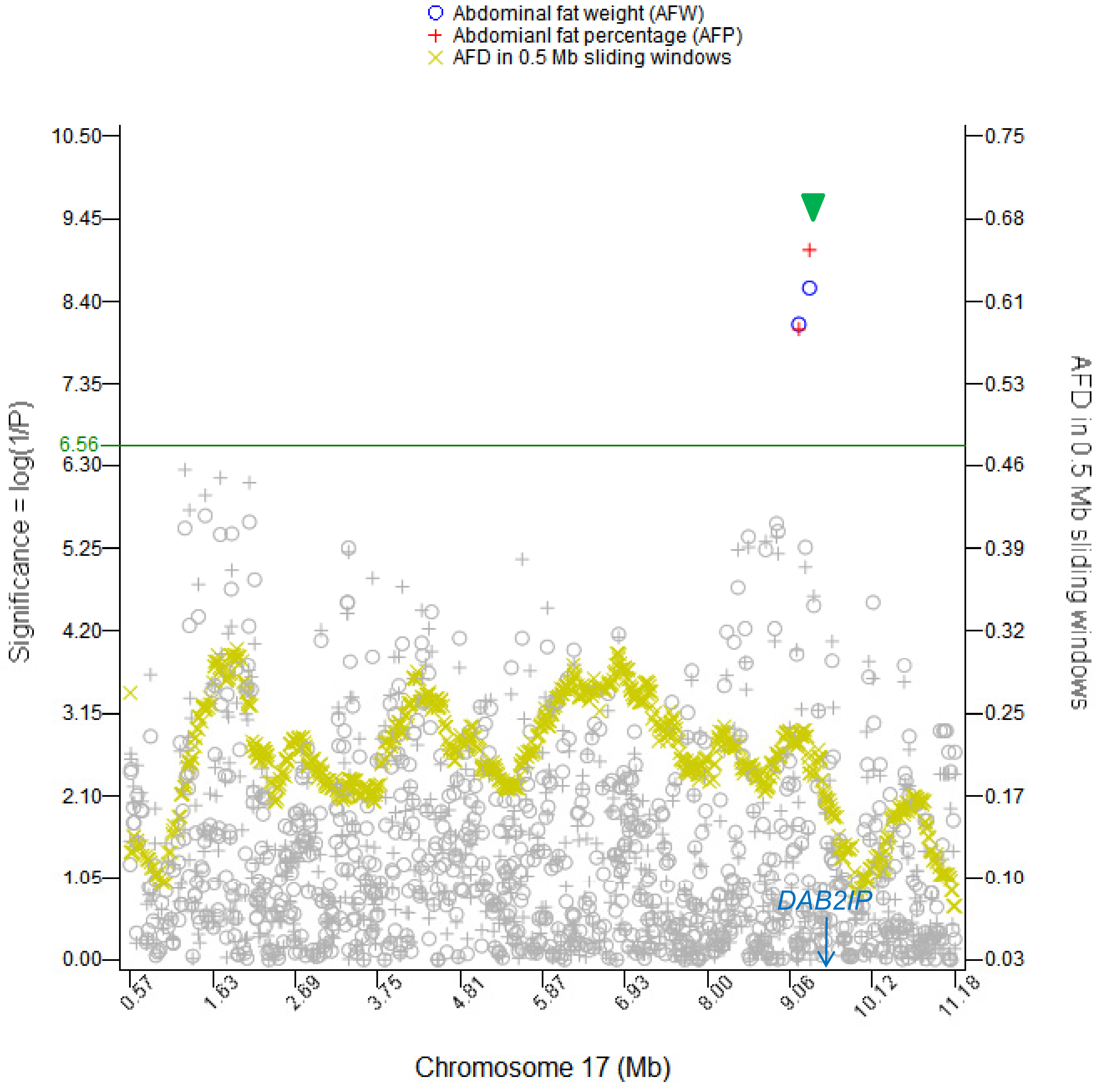

Abdominal fat weight (AFW)
Abdomianl fat percentage (AFP)
AFD in 0.5 Mb sliding windows
Significance = log(1/P)
10.50
9.45
8.40
7.35
6.56
6.30
5.25
4.20
3.15
2.10
1.05
0.00
0.75
0.68
0.61
0.53
0.46
0.39
0.32
0.25
0.17
0.10
0.03
AFD in 0.5 Mb sliding windows
DAB2IP
0.57
1.63
2.69
3.75
4.81
5.87
6.93
8.00
9.06
10.12
11.18
Chromosome 17 (Mb)

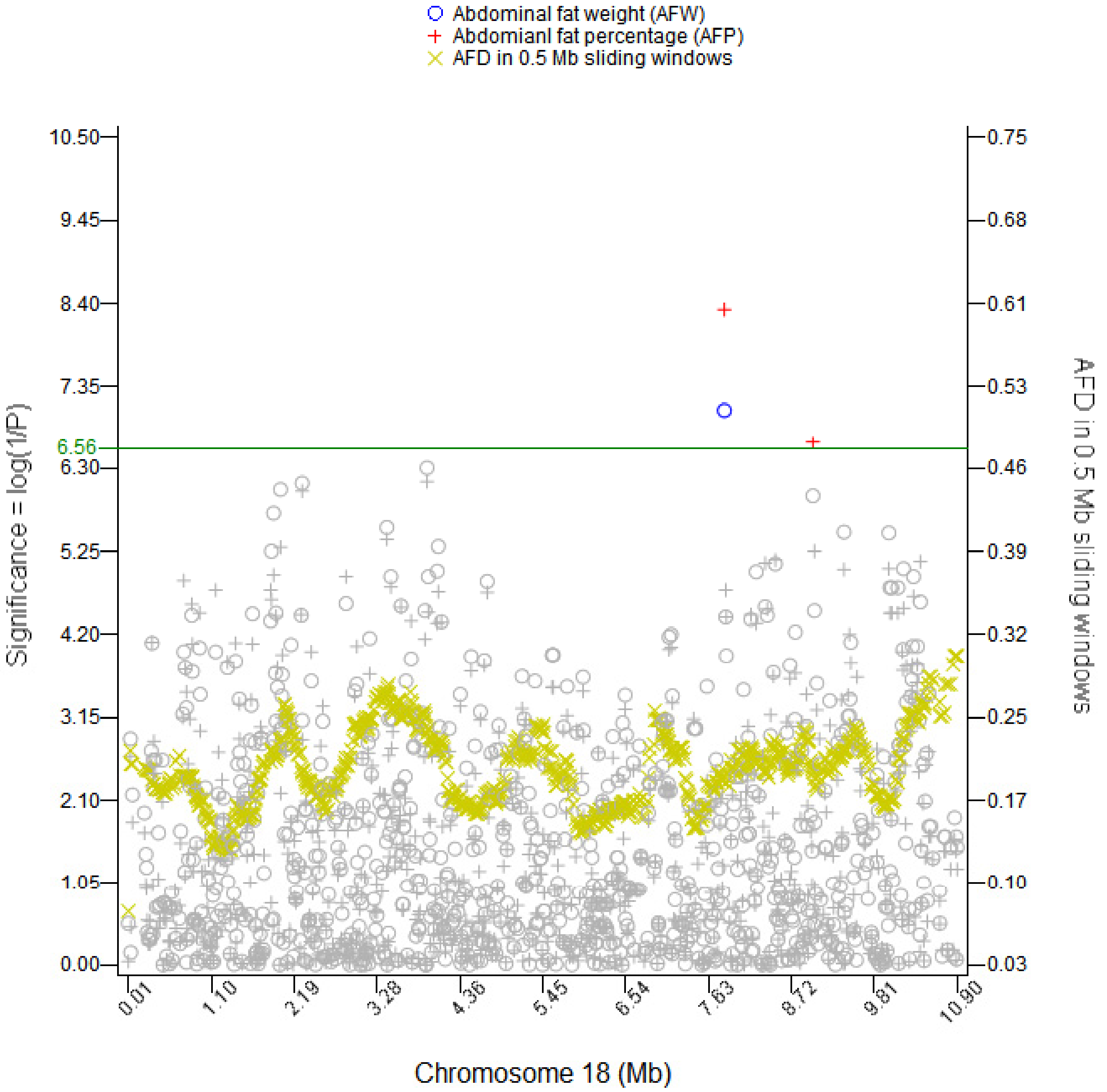

Abdominal fat weight (AFW)
Abdomianl fat percentage (AFP)
AFD in 0.5 Mb sliding windows
Significance = log(1/P)
10.50
9.45
8.40
7.35
6.56
6.30
5.25
4.20
3.15
2.10
1.05
0.00
AFD in 0.5 Mb sliding windows
0.75
0.68
0.61
0.53
0.46
0.39
0.32
0.25
0.17
0.10
0.03
0.01
1.10
2.19
3.28
4.36
5.45
6.54
7.63
8.72
9.81
10.90
Chromosome 18 (Mb)

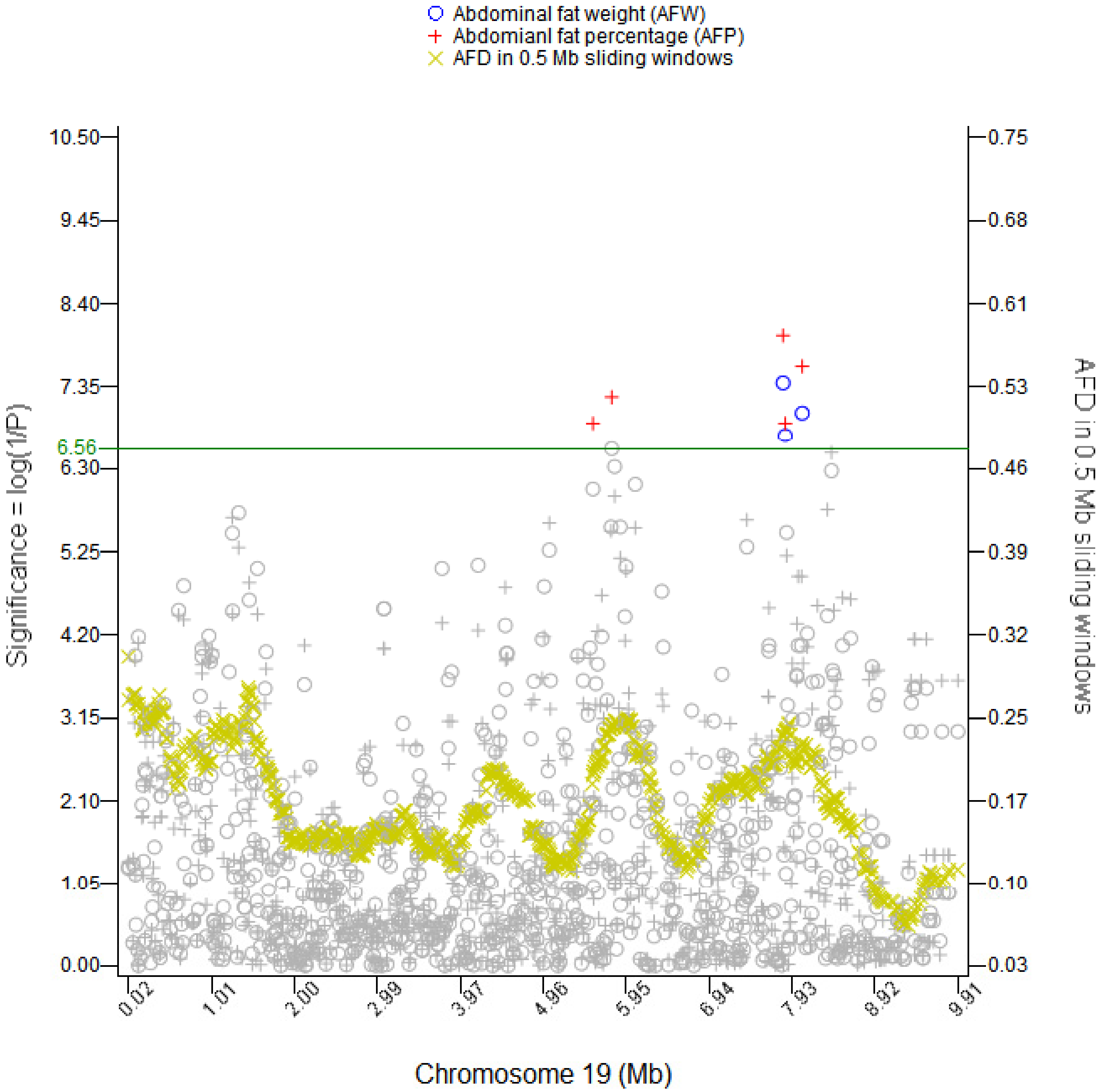

Abdominal fat weight (AFW)
Abdomianl fat percentage (AFP)
AFD in 0.5 Mb sliding windows
Significance = log(1/P)
AFD in 0.5 Mb sliding windows
Chromosome 19 (Mb)
10.50
9.45
8.40
7.35
6.56
6.30
5.25
4.20
3.15
2.10
1.05
0.00
0.75
0.68
0.61
0.53
0.46
0.39
0.32
0.25
0.17
0.10
0.03
0.02
1.01
2.00
2.99
3.97
4.96
5.95
6.94
7.93
8.92
9.91

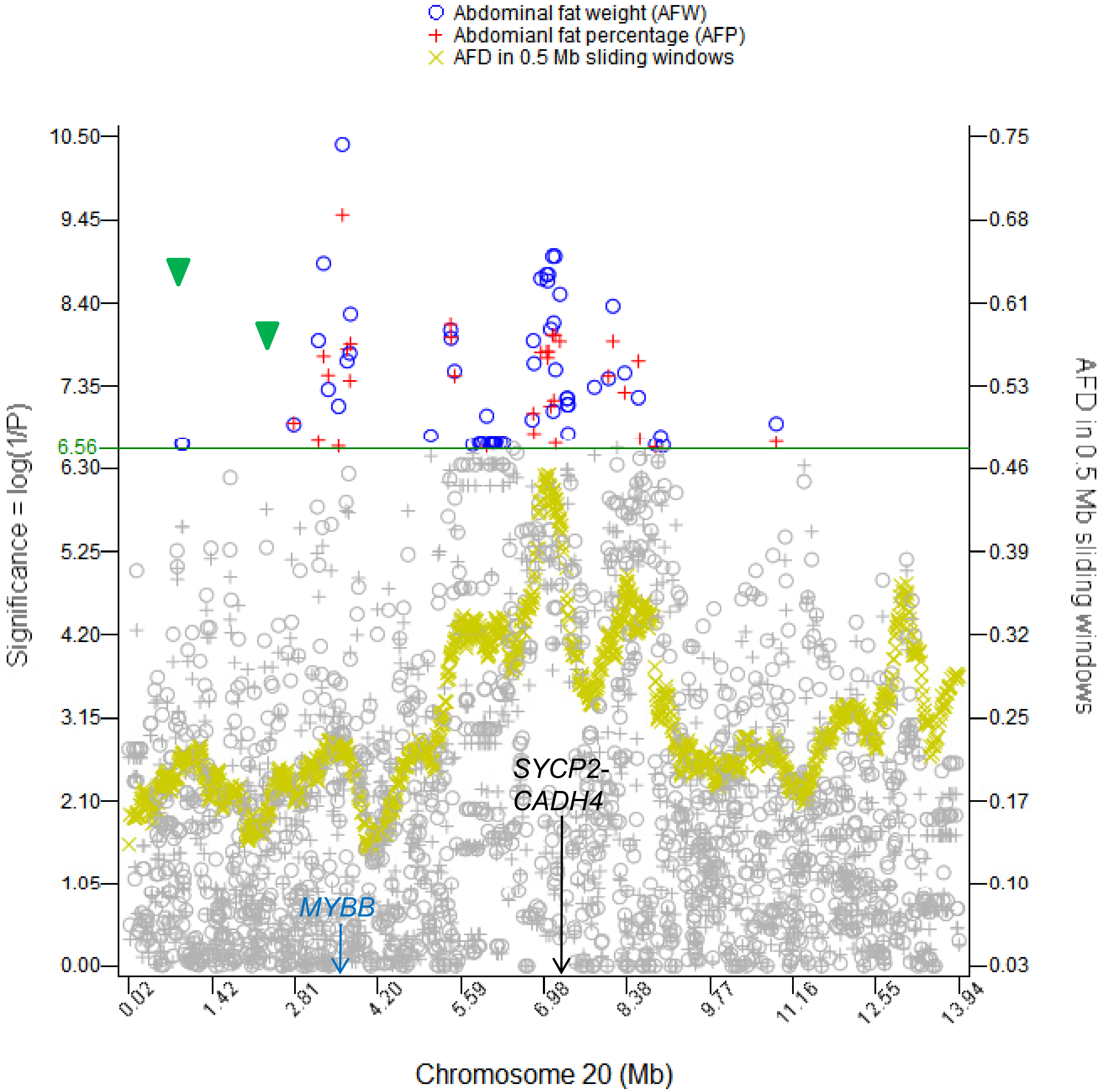

Abdominal fat weight (AFW)
Abdomianl fat percentage (AFP)
AFD in 0.5 Mb sliding windows
Significance = log(1/P)
10.50
9.45
8.40
7.35
6.56
6.30
5.25
4.20
3.15
2.10
1.05
0.00
AFD in 0.5 Mb sliding windows
0.75
0.68
0.61
0.53
0.46
0.39
0.32
0.25
0.17
0.10
0.03
SYCP2-
CADH4
MYBB
0.02
1.42
2.81
4.20
5.59
6.98
8.38
9.77
11.16
12.55
13.94
Chromosome 20 (Mb)

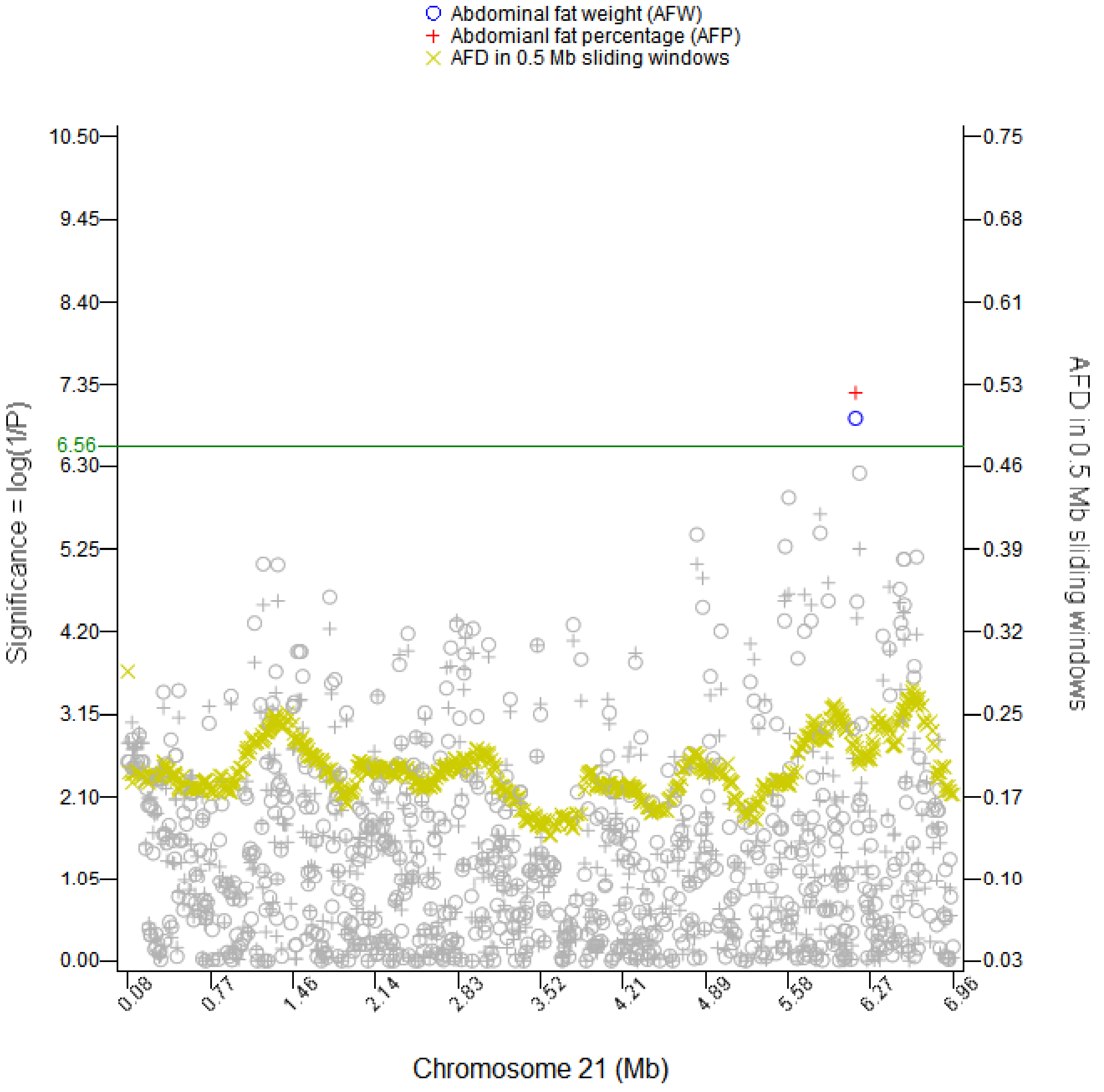

Abdominal fat weight (AFW)
Abdomianl fat percentage (AFP)
AFD in 0.5 Mb sliding windows
Significance = log(1/P)
AFD in 0.5 Mb sliding windows
6.56
10.50
9.45
8.40
7.35
6.30
5.25
4.20
3.15
2.10
1.05
0.00
0.75
0.68
0.61
0.53
0.46
0.39
0.32
0.25
0.17
0.10
0.03
0.08
0.77
1.46
2.14
2.83
3.52
4.21
4.89
5.58
6.27
6.96
Chromosome 21 (Mb)

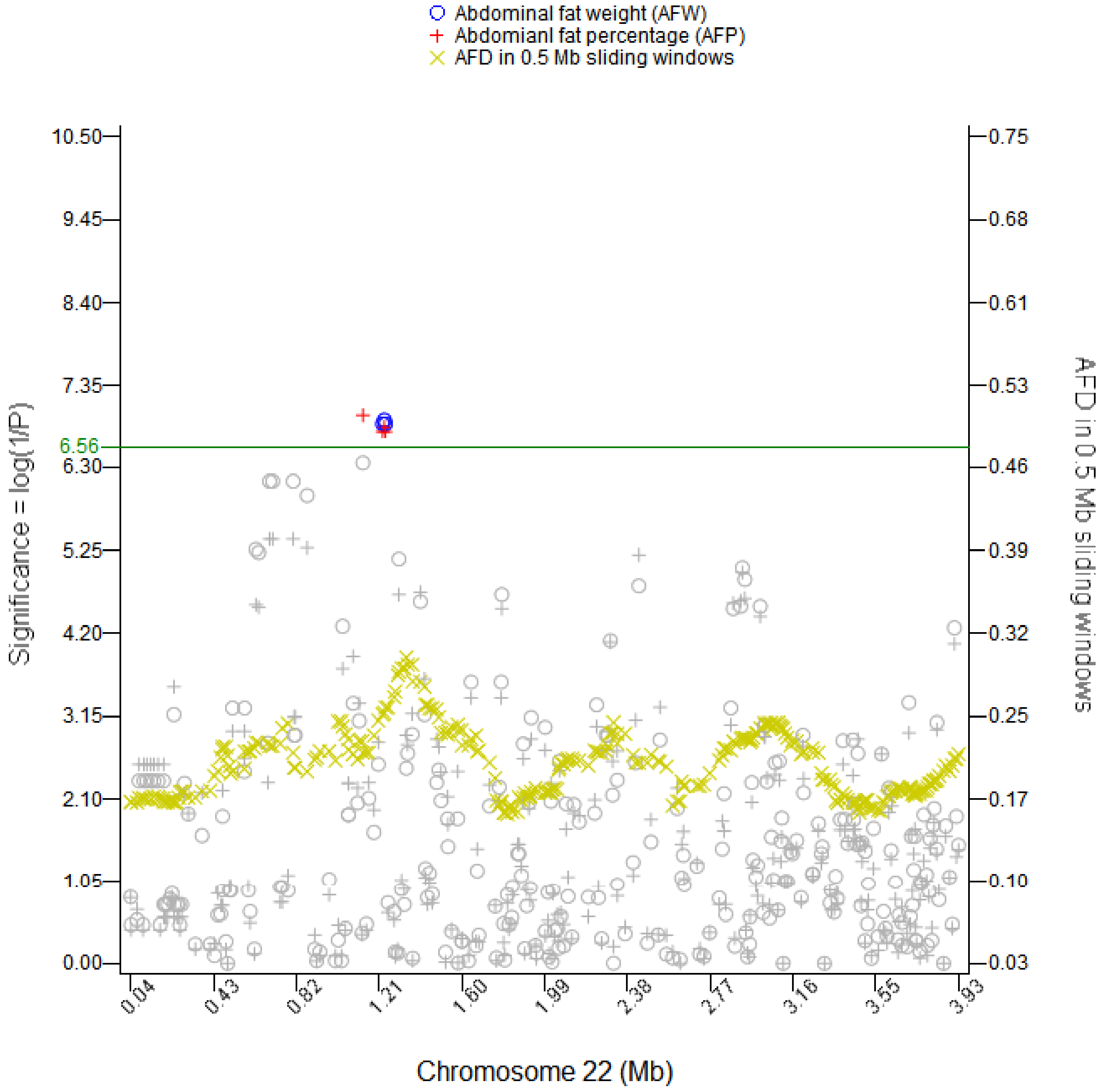
Abdominal fat weight (AFW)
Abdomianl fat percentage (AFP)
AFD in 0.5 Mb sliding windows
Significance = log(1/P)
AFD in 0.5 Mb sliding windows
Chromosome 22 (Mb)
10.50
9.45
8.40
7.35
6.56
6.30
5.25
4.20
3.15
2.10
1.05
0.00
0.75
0.68
0.61
0.53
0.46
0.39
0.32
0.25
0.17
0.10
0.03
0.04
0.43
0.82
1.21
1.60
1.99
2.38
2.77
3.16
3.55
3.93

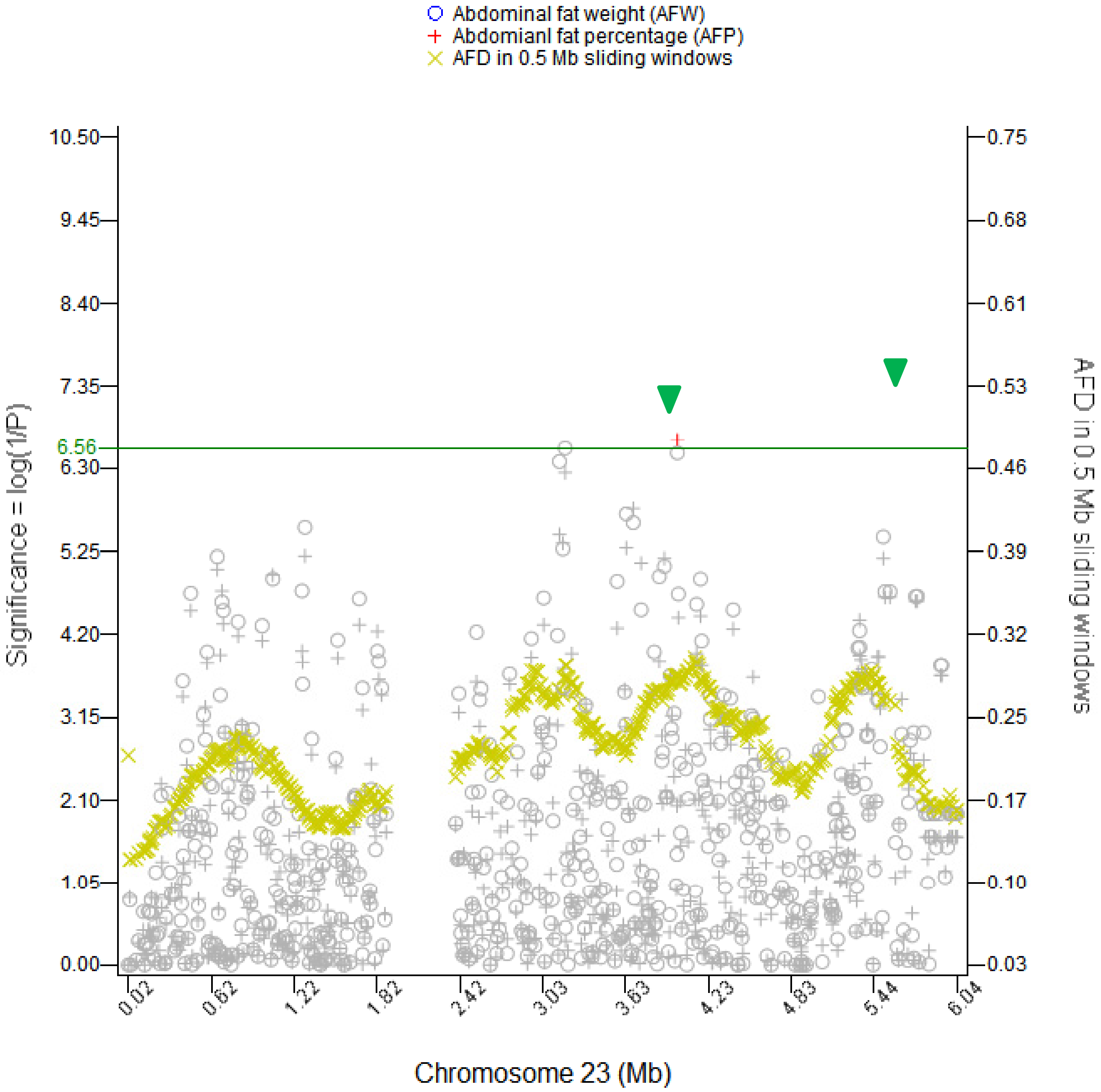
Abdominal fat weight (AFW)
Abdomianl fat percentage (AFP)
AFD in 0.5 Mb sliding windows
Significance = log(1/P)
10.50
9.45
8.40
7.35
6.56
6.30
5.25
4.20
3.15
2.10
1.05
0.00
0.75
0.68
0.61
0.53
0.46
0.39
0.32
0.25
0.17
0.10
0.03
AFD in 0.5 Mb sliding windows
0.02
0.62
1.22
1.82
2.42
3.03
3.63
4.23
4.83
5.44
6.04
Chromosome 23 (Mb)

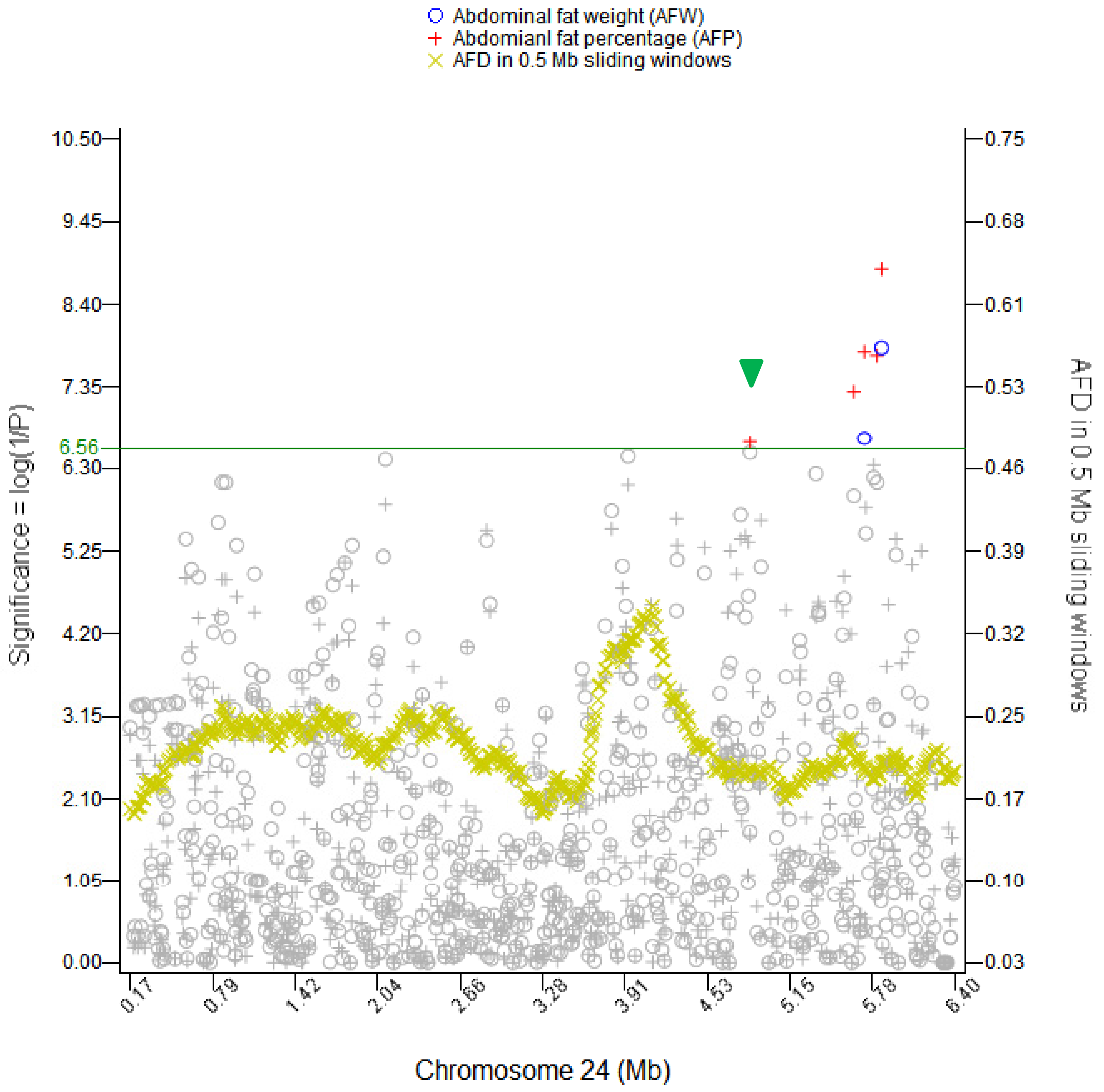

Abdominal fat weight (AFW)
Abdomianl fat percentage (AFP)
AFD in 0.5 Mb sliding windows
Significance = log(1/P)
AFD in 0.5 Mb sliding windows
Chromosome 24 (Mb)
10.50
9.45
8.40
7.35
6.56
6.30
5.25
4.20
3.15
2.10
1.05
0.00
0.75
0.68
0.61
0.53
0.46
0.39
0.32
0.25
0.17
0.10
0.03
0.17
0.79
1.42
2.04
2.66
3.28
3.91
4.53
5.15
5.78
6.40

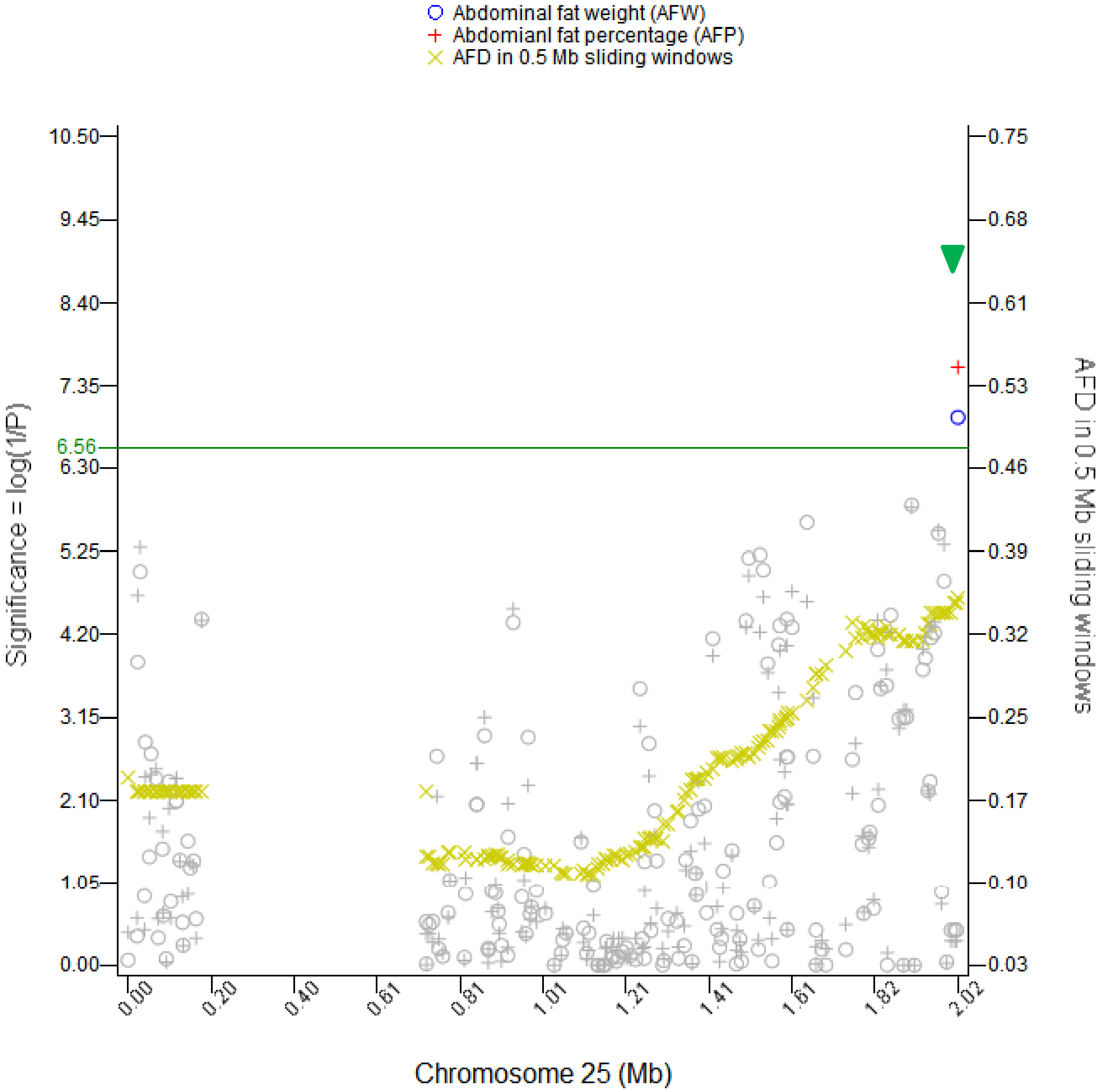

Abdominal fat weight (AFW)
Abdomianl fat percentage (AFP)
AFD in 0.5 Mb sliding windows
Significance = log(1/P)
AFD in 0.5 Mb sliding windows
Chromosome 25 (Mb)
10.50
9.45
8.40
7.35
6.56
6.30
5.25
4.20
3.15
2.10
1.05
0.00
0.75
0.68
0.61
0.53
0.46
0.39
0.32
0.25
0.17
0.10
0.03
0.00
0.20
0.40
0.61
0.81
1.01
1.21
1.41
1.61
1.82
2.02

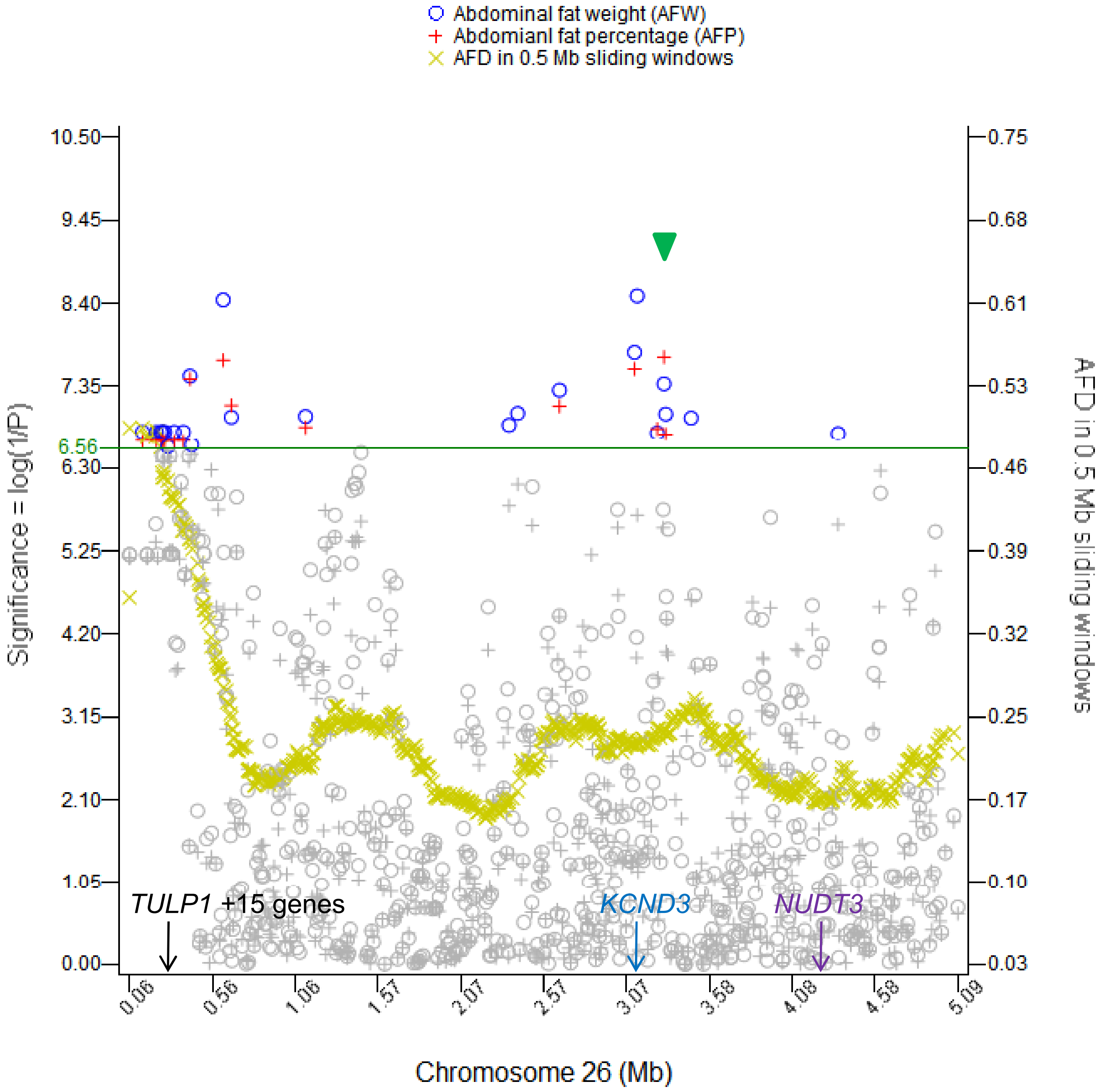

Abdominal fat weight (AFW)
Abdomianl fat percentage (AFP)
AFD in 0.5 Mb sliding windows
Significance = log(1/P)
AFD in 0.5 Mb sliding windows
10.50
9.45
8.40
7.35
6.56
6.30
5.25
4.20
3.15
2.10
1.05
0.00
0.75
0.68
0.61
0.53
0.46
0.39
0.32
0.25
0.17
0.10
0.03
TULP1 +15 genes
KCND3
NUDT3
0.06
0.56
1.06
1.57
2.07
2.57
3.07
3.58
4.08
4.58
5.09
Chromosome 26 (Mb)

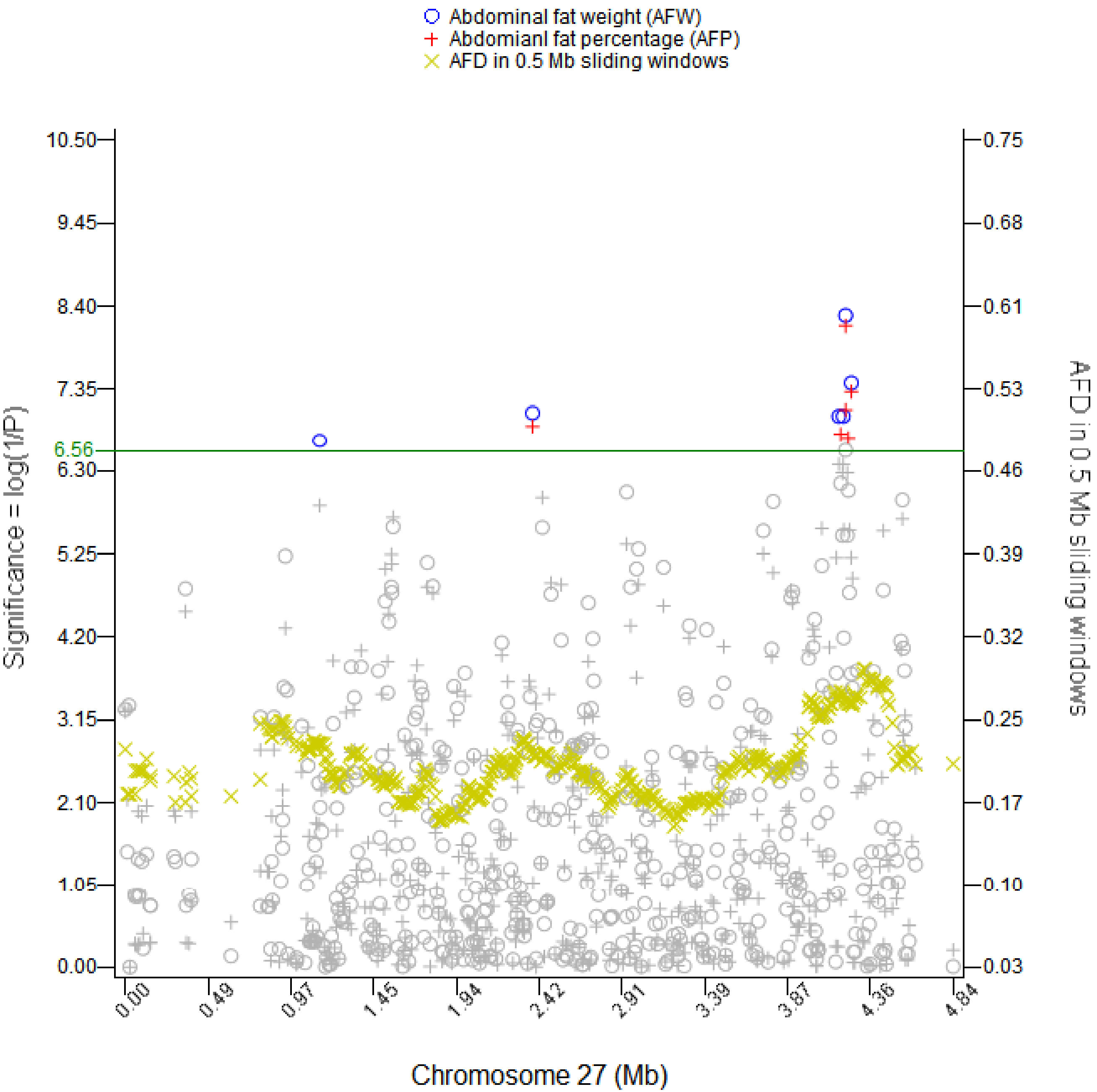

Abdominal fat weight (AFW)
Abdomianl fat percentage (AFP)
AFD in 0.5 Mb sliding windows
Significance = log(1/P)
10.50
9.45
8.40
7.35
6.56
6.30
5.25
4.20
3.15
2.10
1.05
0.00
AFD in 0.5 Mb sliding windows
0.75
0.68
0.61
0.53
0.46
0.39
0.32
0.25
0.17
0.10
0.03
0.00
0.49
0.97
1.45
1.94
2.42
2.91
3.39
3.87
4.36
4.84
Chromosome 27 (Mb)

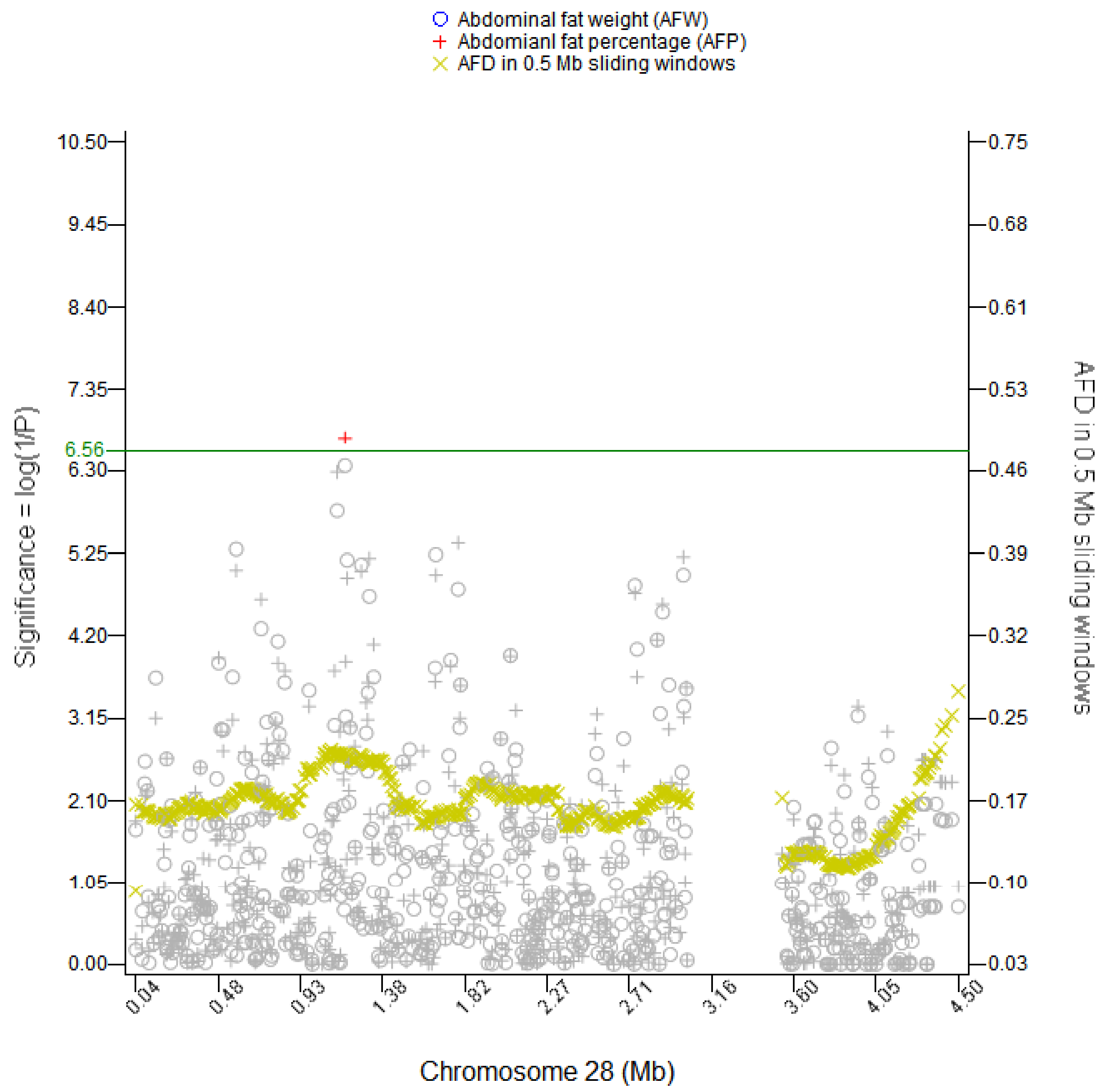

Abdominal fat weight (AFW)
Abdomianl fat percentage (AFP)
AFD in 0.5 Mb sliding windows
Significance = log(1/P)
10.50
9.45
8.40
7.35
6.56
6.30
5.25
4.20
3.15
2.10
1.05
0.00
AFD in 0.5 Mb sliding windows
0.75
0.68
0.61
0.53
0.46
0.39
0.32
0.25
0.17
0.10
0.03
0.04
0.48
0.93
1.38
1.82
2.27
2.71
3.16
3.60
4.05
4.50
Chromosome 28 (Mb)

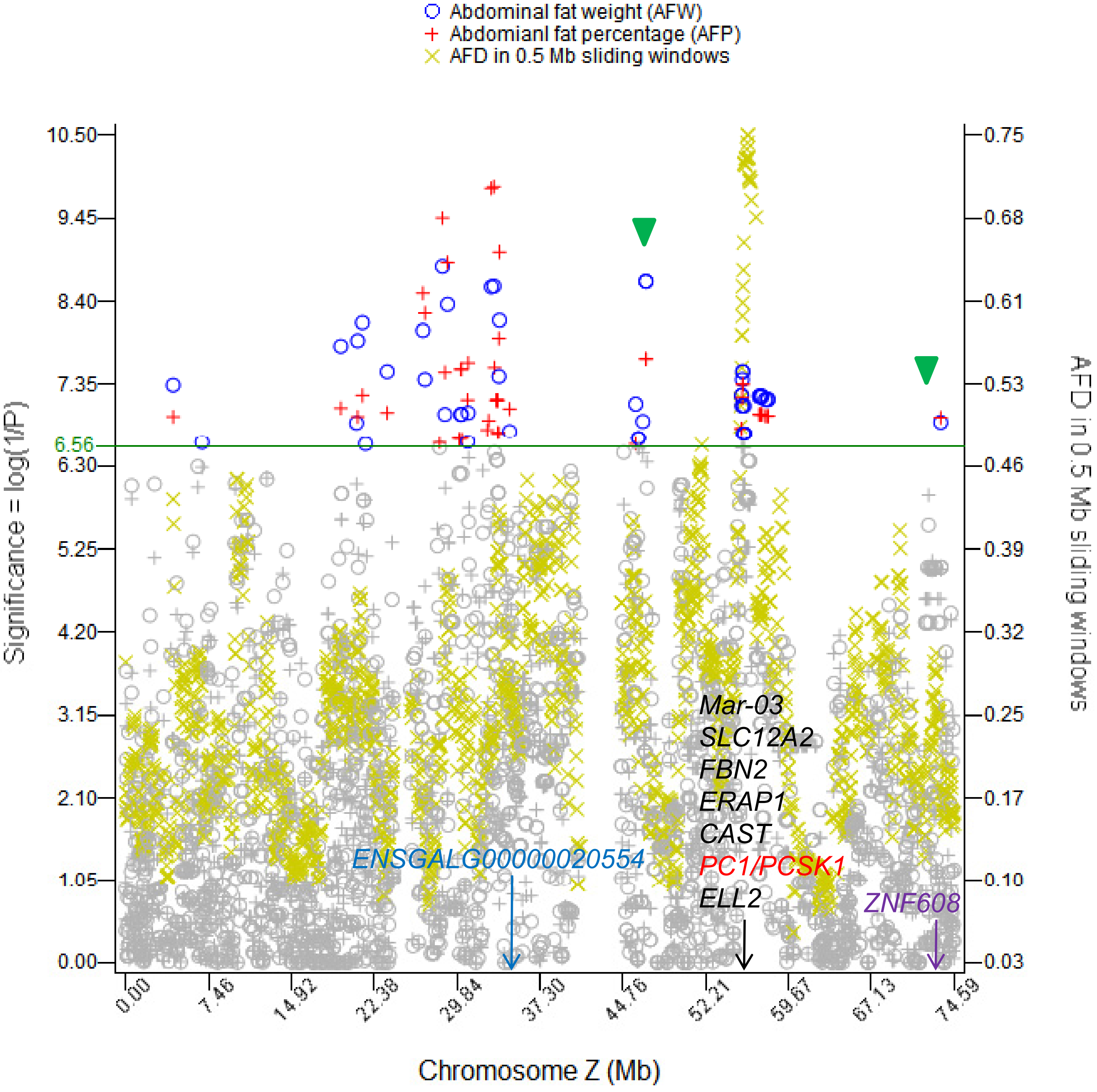

Abdominal fat weight (AFW)
Abdomianl fat percentage (AFP)
AFD in 0.5 Mb sliding windows
Significance = log(1/P)
AFD in 0.5 Mb sliding windows
Chromosome Z (Mb)
10.50
9.45
8.40
7.35
6.56
6.30
5.25
4.20
3.15
2.10
1.05
0.00
0.75
0.68
0.61
0.53
0.46
0.39
0.32
0.25
0.17
0.10
0.03
0.00
7.46
14.92
22.38
29.84
37.30
44.76
52.21
59.67
67.13
74.59
ENSGALG00000020554
Mar-03
SLC12A2
FBN2
ERAP1
CAST
PC1/PCSK1
ELL2
ZNF608
