# Supplementary material for: Selection Signature Analysis Implicates the PC1/PCSK1 Region for Chicken Abdominal Fat Content
Source: PLoS One. 2012 Jul 11;7(7):e40736. doi: 10.1371/journal.pone.0040736 (PMC3394724; doi:10.1371/journal.pone.0040736)
Supplement: Figure S2 — AFD and Z values in 0.5 Mb sliding windows of SNP markers. (PDF) [file pone.0040736.s002.pdf]

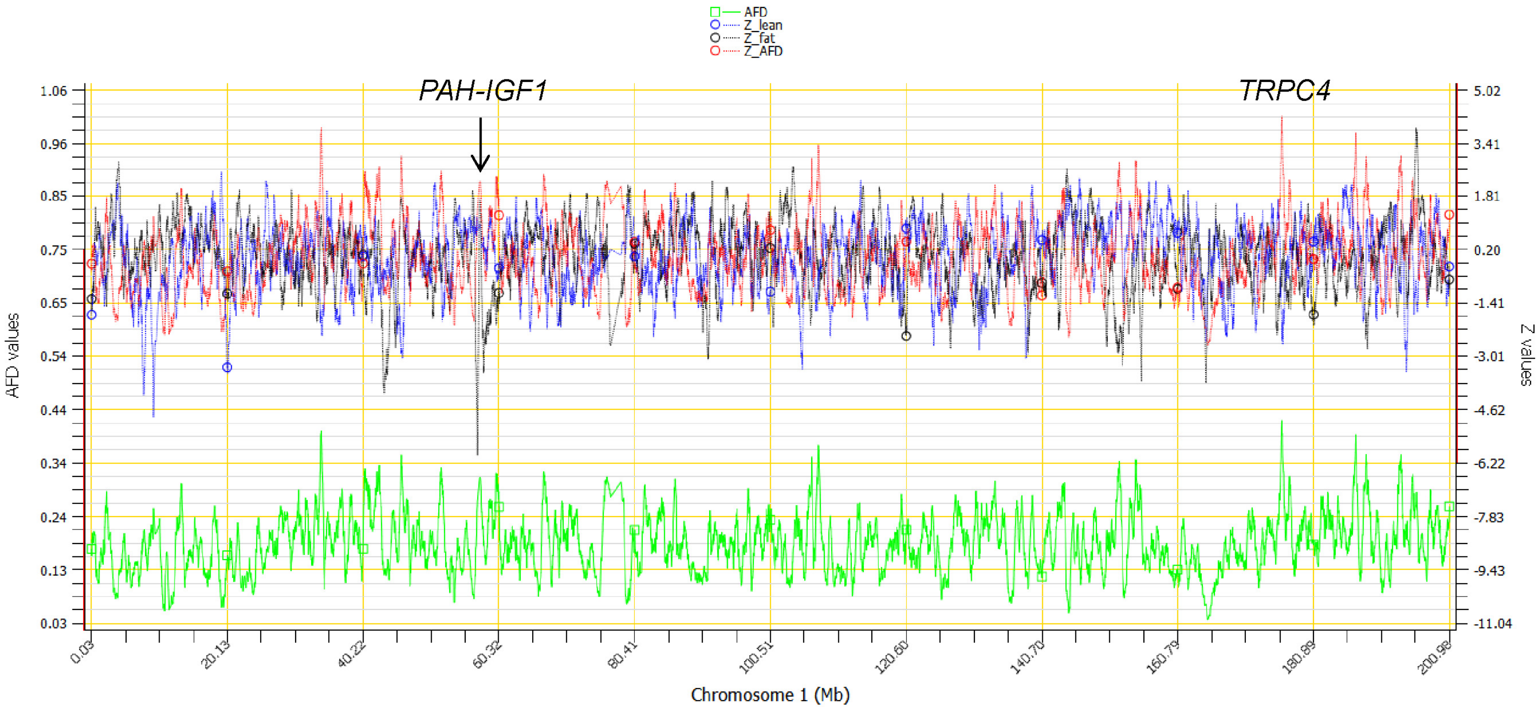
AFD
Z_lean
Z_fat
Z_AFD
PAH-IGF1
TRPC4
AFD values
Z values
1.06
0.96
0.85
0.75
0.65
0.54
0.44
0.34
0.24
0.13
0.03
5.02
3.41
1.81
0.20
-1.41
-3.01
-4.62
-6.22
-7.83
-9.43
-11.04
0.03
20.13
40.22
60.32
80.41
100.51
120.60
140.70
160.79
180.89
200.98
Chromosome 1 (Mb)

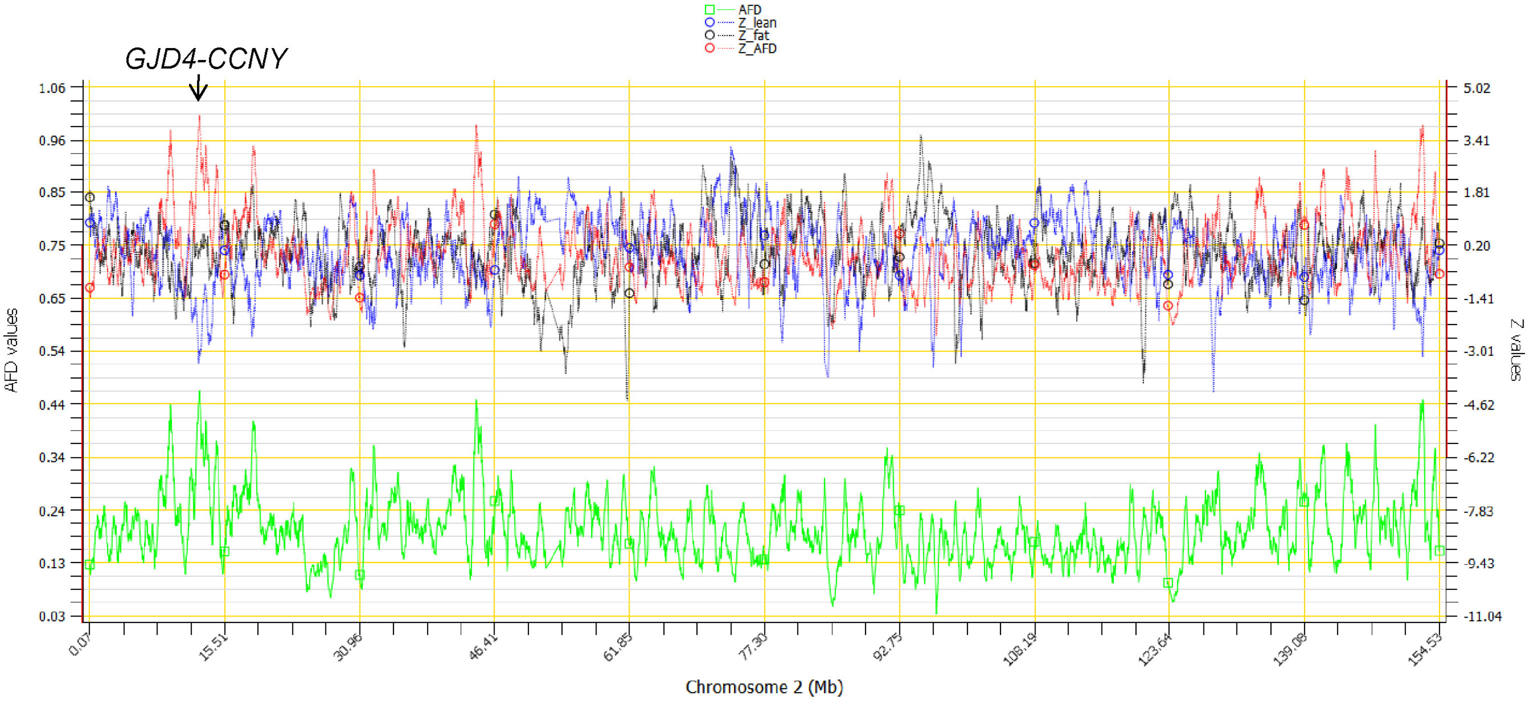
AFD
Z_lean
Z_fat
Z_AFD
GJD4-CCNY
AFD values
Z values
1.06
0.96
0.85
0.75
0.65
0.54
0.44
0.34
0.24
0.13
0.03
5.02
3.41
1.81
0.20
-1.41
-3.01
-4.62
-6.22
-7.83
-9.43
-11.04
0.07
15.51
30.96
46.41
61.85
77.30
92.75
108.19
123.64
139.08
154.53
Chromosome 2 (Mb)

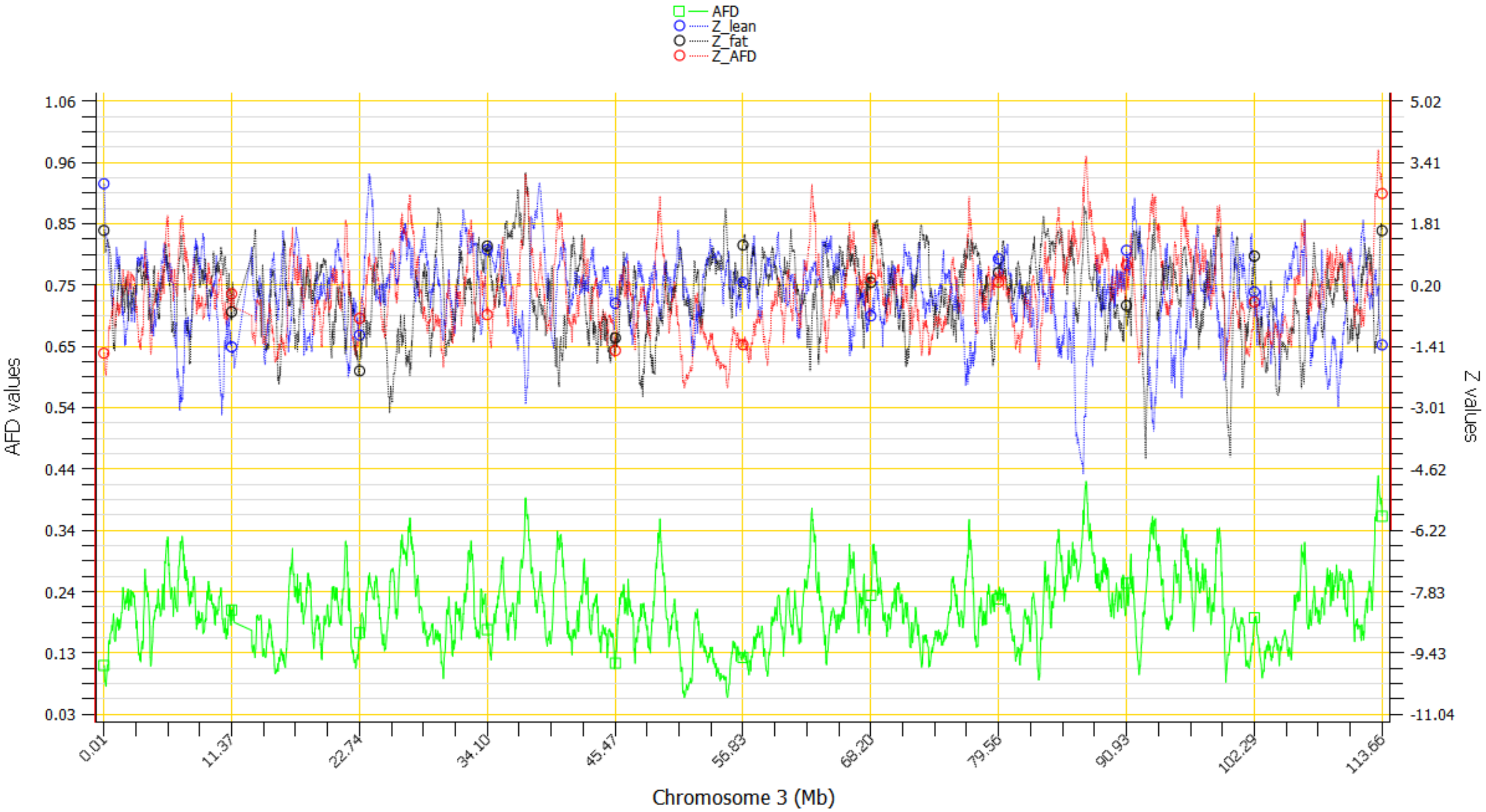
AFD
Z_lean
Z_fat
Z_AFD
AFD values
Z values
Chromosome 3 (Mb)
0.01
11.37
22.74
34.10
45.47
56.83
68.20
79.56
90.93
102.29
113.66
1.06
0.96
0.85
0.75
0.65
0.54
0.44
0.34
0.24
0.13
0.03
5.02
3.41
1.81
0.20
-1.41
-3.01
-4.62
-6.22
-7.83
-9.43
-11.04

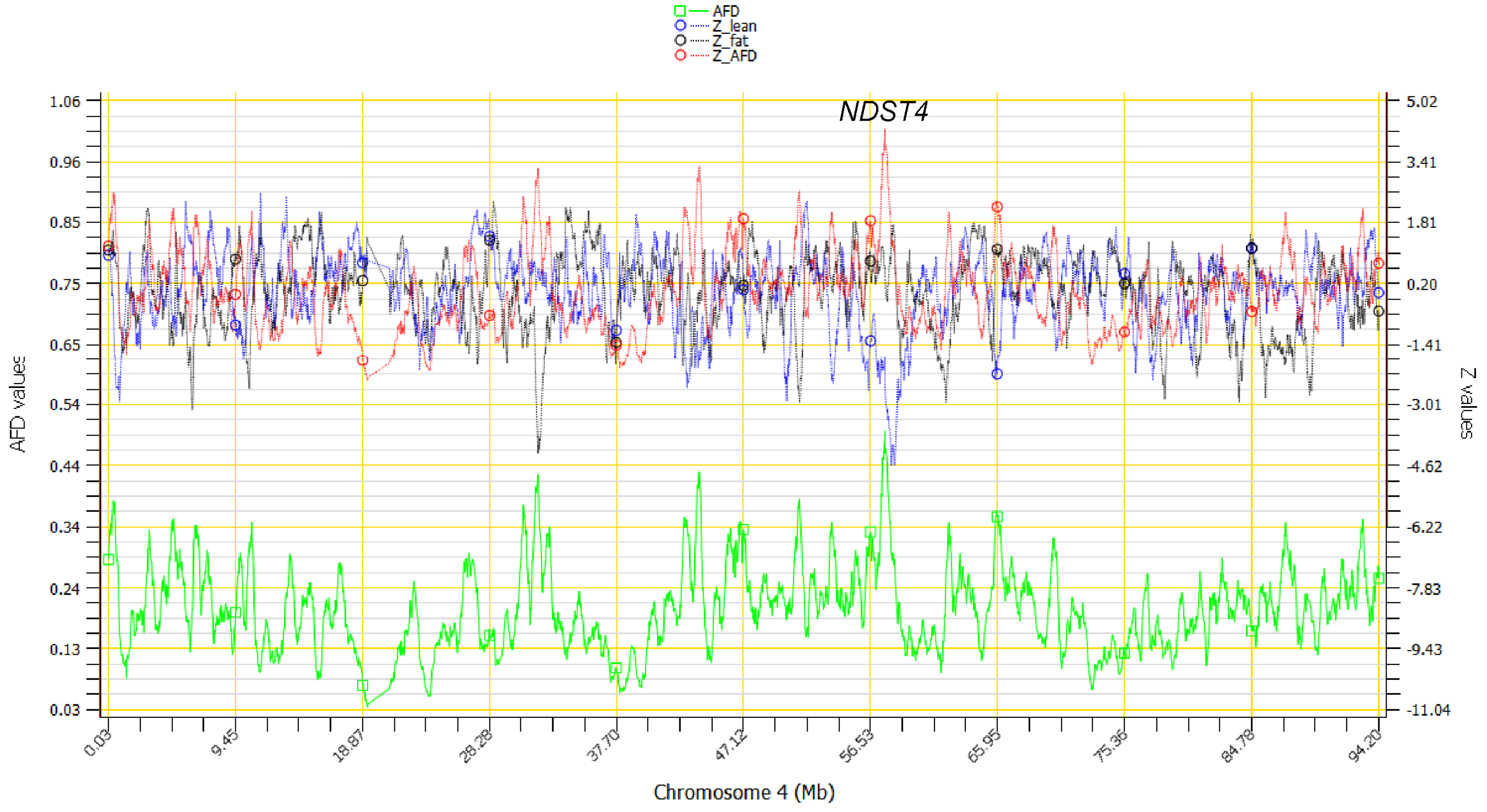
AFD
Z_lean
Z_fat
Z_AFD
NDST4
AFD values
Z values
Chromosome 4 (Mb)
0.03
9.45
18.87
28.28
37.70
47.12
56.53
65.95
75.36
84.78
94.20
1.06
0.96
0.85
0.75
0.65
0.54
0.44
0.34
0.24
0.13
0.03
5.02
3.41
1.81
0.20
-1.41
-3.01
-4.62
-6.22
-7.83
-9.43
-11.04

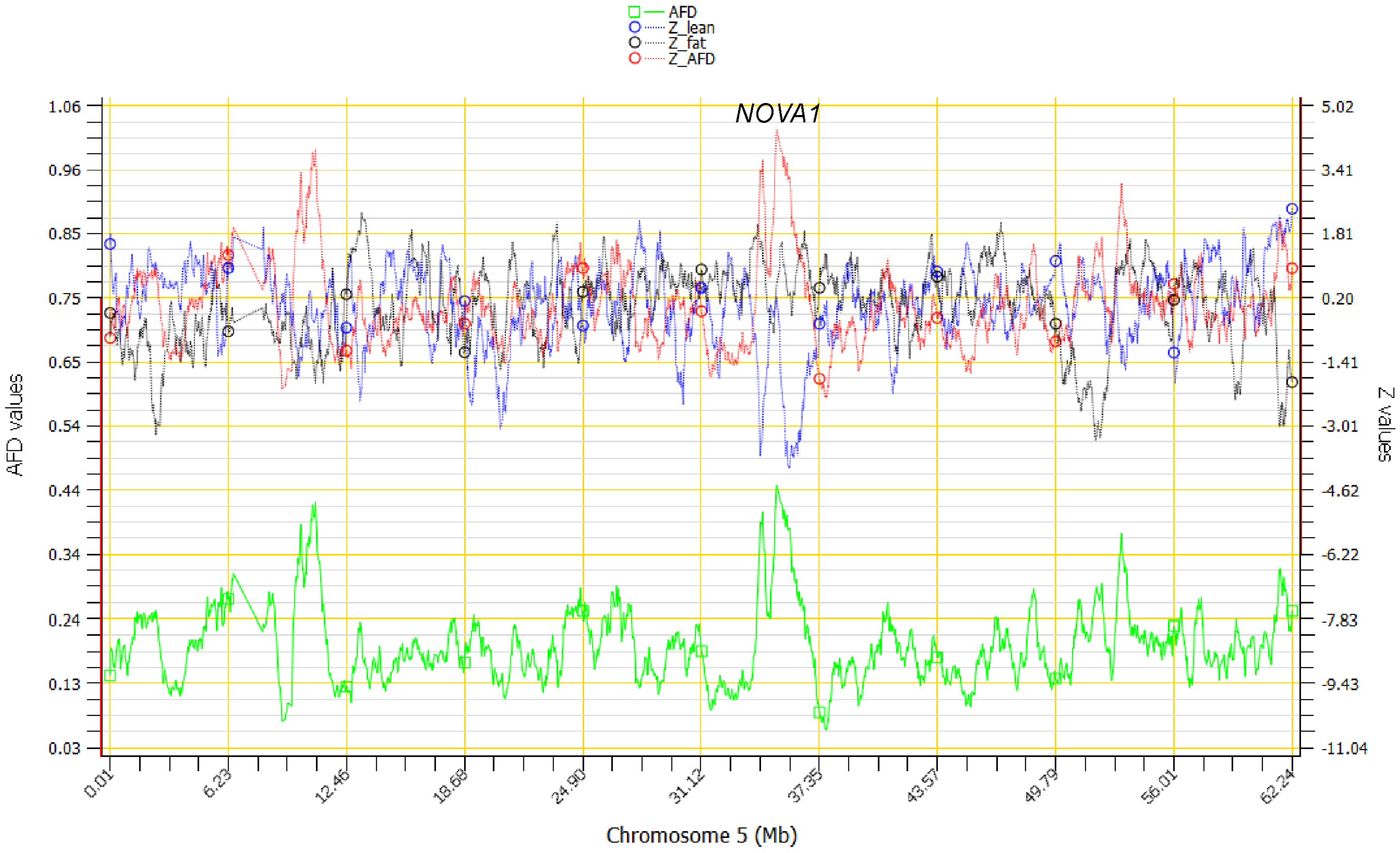
AFD
Z_lean
Z_fat
Z_AFD
NOVA1
AFD values
Z values
1.06
0.96
0.85
0.75
0.65
0.54
0.44
0.34
0.24
0.13
0.03
5.02
3.41
1.81
0.20
-1.41
-3.01
-4.62
-6.22
-7.83
-9.43
-11.04
0.01
6.23
12.46
18.68
24.90
31.12
37.35
43.57
49.79
56.01
62.24
Chromosome 5 (Mb)

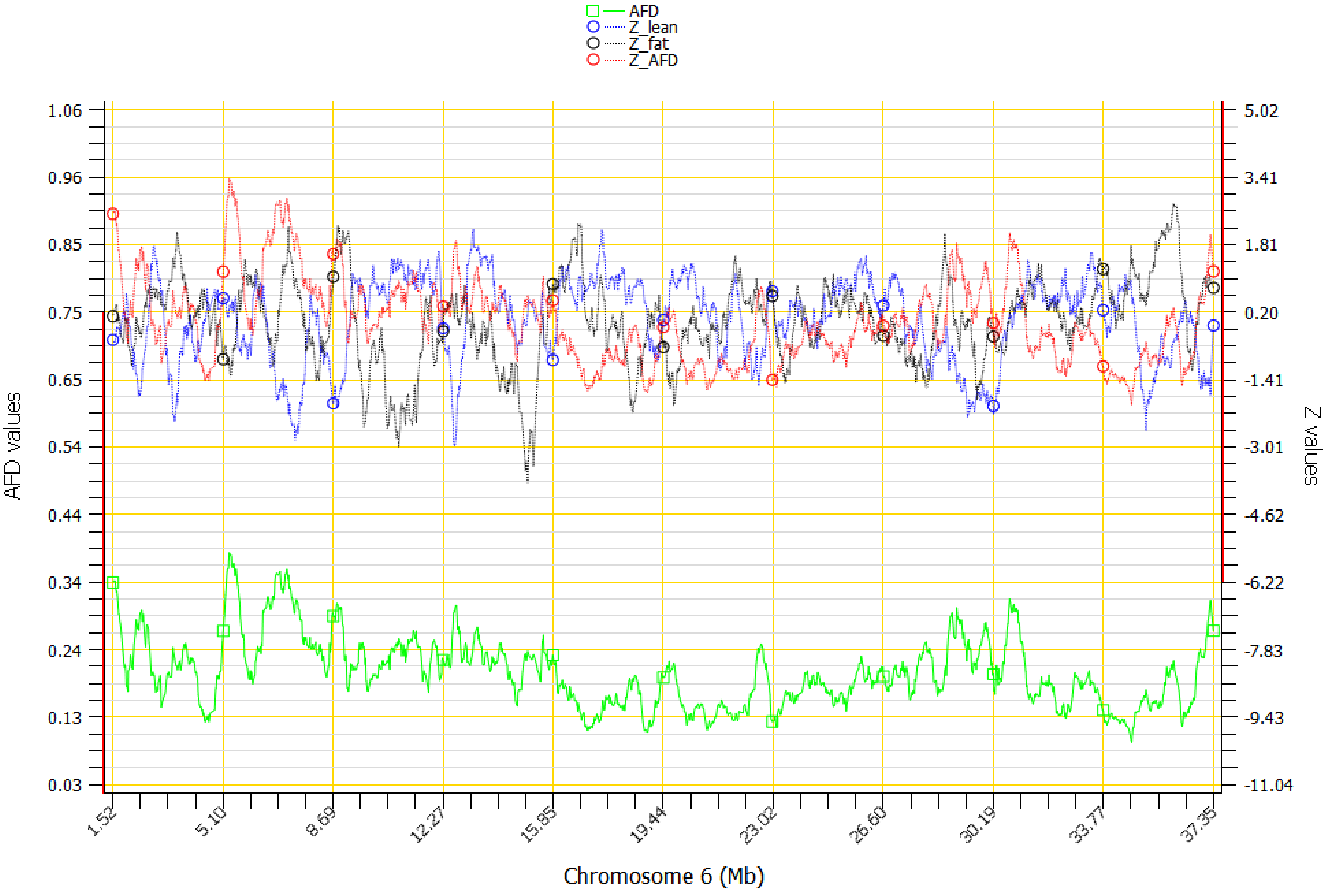
AFD
Z_lean
Z_fat
Z_AFD
AFD values
Z values
1.06
0.96
0.85
0.75
0.65
0.54
0.44
0.34
0.24
0.13
0.03
5.02
3.41
1.81
0.20
-1.41
-3.01
-4.62
-6.22
-7.83
-9.43
-11.04
1.52
5.10
8.69
12.27
15.85
19.44
23.02
26.60
30.19
33.77
37.35
Chromosome 6 (Mb)

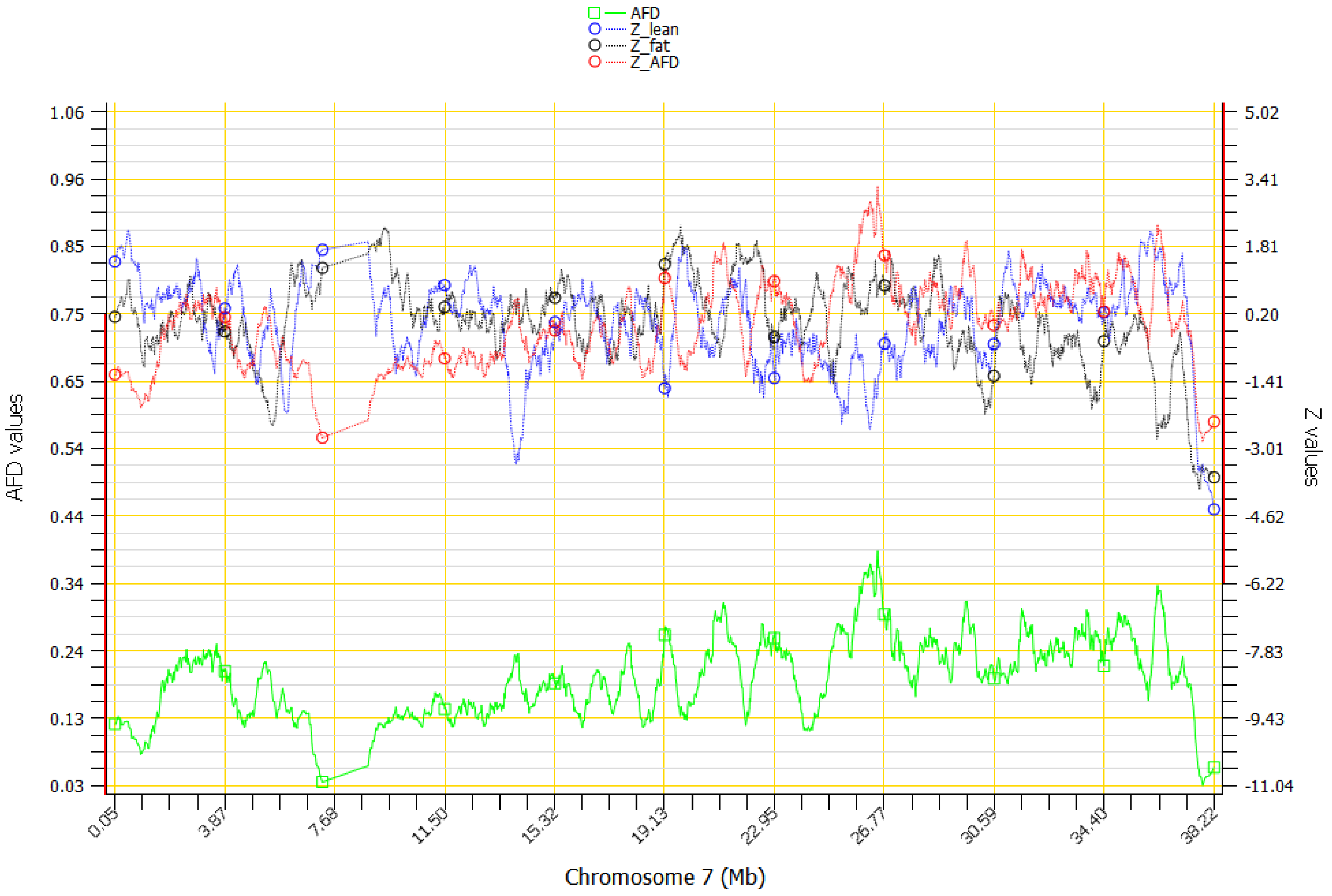

AFD
Z_lean
Z_fat
Z_AFD
AFD values
Z values
1.06
0.96
0.85
0.75
0.65
0.54
0.44
0.34
0.24
0.13
0.03
5.02
3.41
1.81
0.20
-1.41
-3.01
-4.62
-6.22
-7.83
-9.43
-11.04
0.05
3.87
7.68
11.50
15.32
19.13
22.95
26.77
30.59
34.40
38.22
Chromosome 7 (Mb)

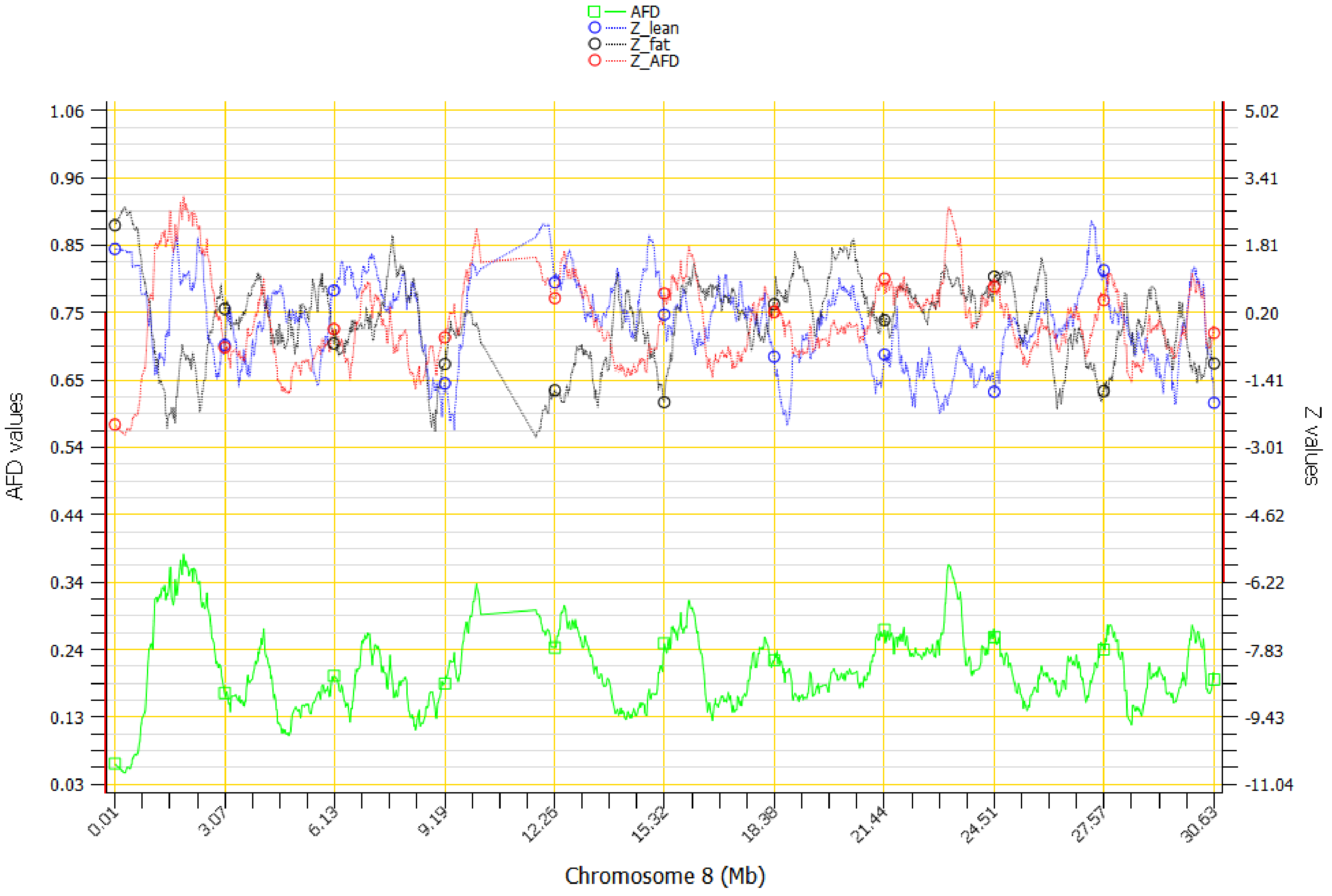

AFD
Z_lean
Z_fat
Z_AFD
AFD values
Z values
1.06
0.96
0.85
0.75
0.65
0.54
0.44
0.34
0.24
0.13
0.03
5.02
3.41
1.81
0.20
-1.41
-3.01
-4.62
-6.22
-7.83
-9.43
-11.04
0.01
3.07
6.13
9.19
12.26
15.32
18.38
21.44
24.51
27.57
30.63
Chromosome 8 (Mb)

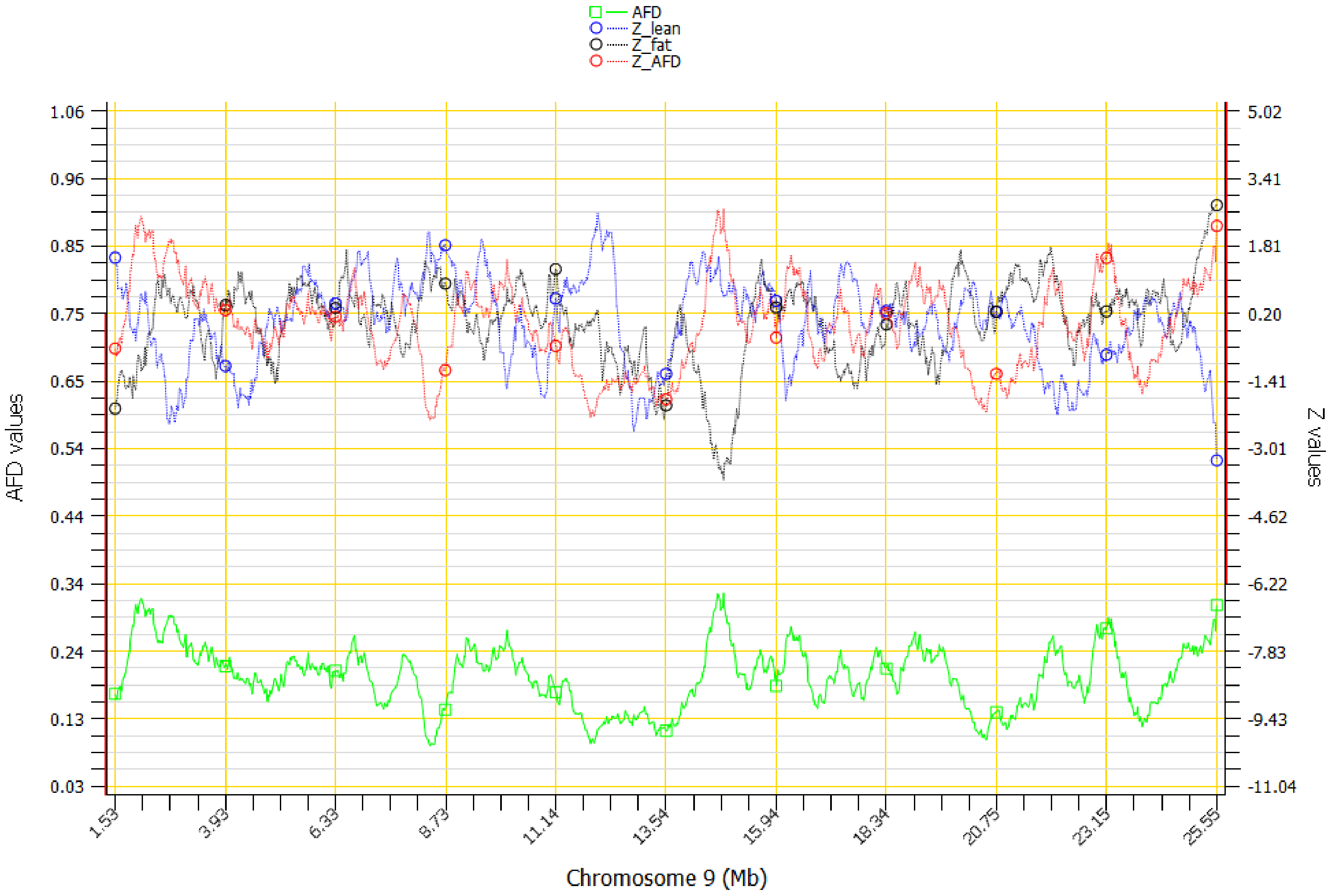

AFD
Z_lean
Z_fat
Z_AFD
AFD values
Z values
1.06
0.96
0.85
0.75
0.65
0.54
0.44
0.34
0.24
0.13
0.03
5.02
3.41
1.81
0.20
-1.41
-3.01
-4.62
-6.22
-7.83
-9.43
-11.04
1.53
3.93
6.33
8.73
11.14
13.54
15.94
18.34
20.75
23.15
25.55
Chromosome 9 (Mb)

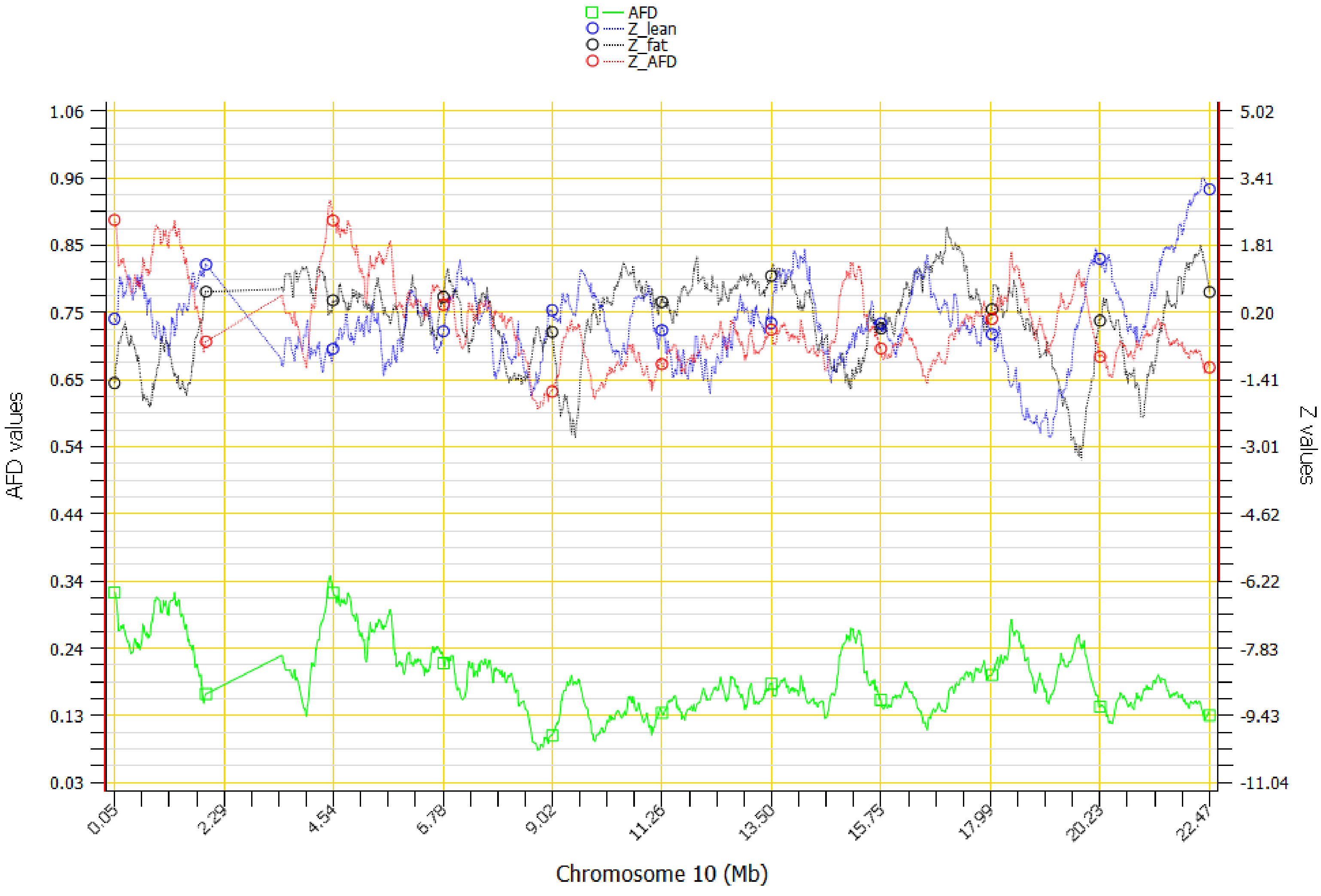

AFD
Z_lean
Z_fat
Z_AFD
AFD values
Z values
1.06
0.96
0.85
0.75
0.65
0.54
0.44
0.34
0.24
0.13
0.03
5.02
3.41
1.81
0.20
-1.41
-3.01
-4.62
-6.22
-7.83
-9.43
-11.04
0.05
2.29
4.54
6.78
9.02
11.26
13.50
15.75
17.99
20.23
22.47
Chromosome 10 (Mb)

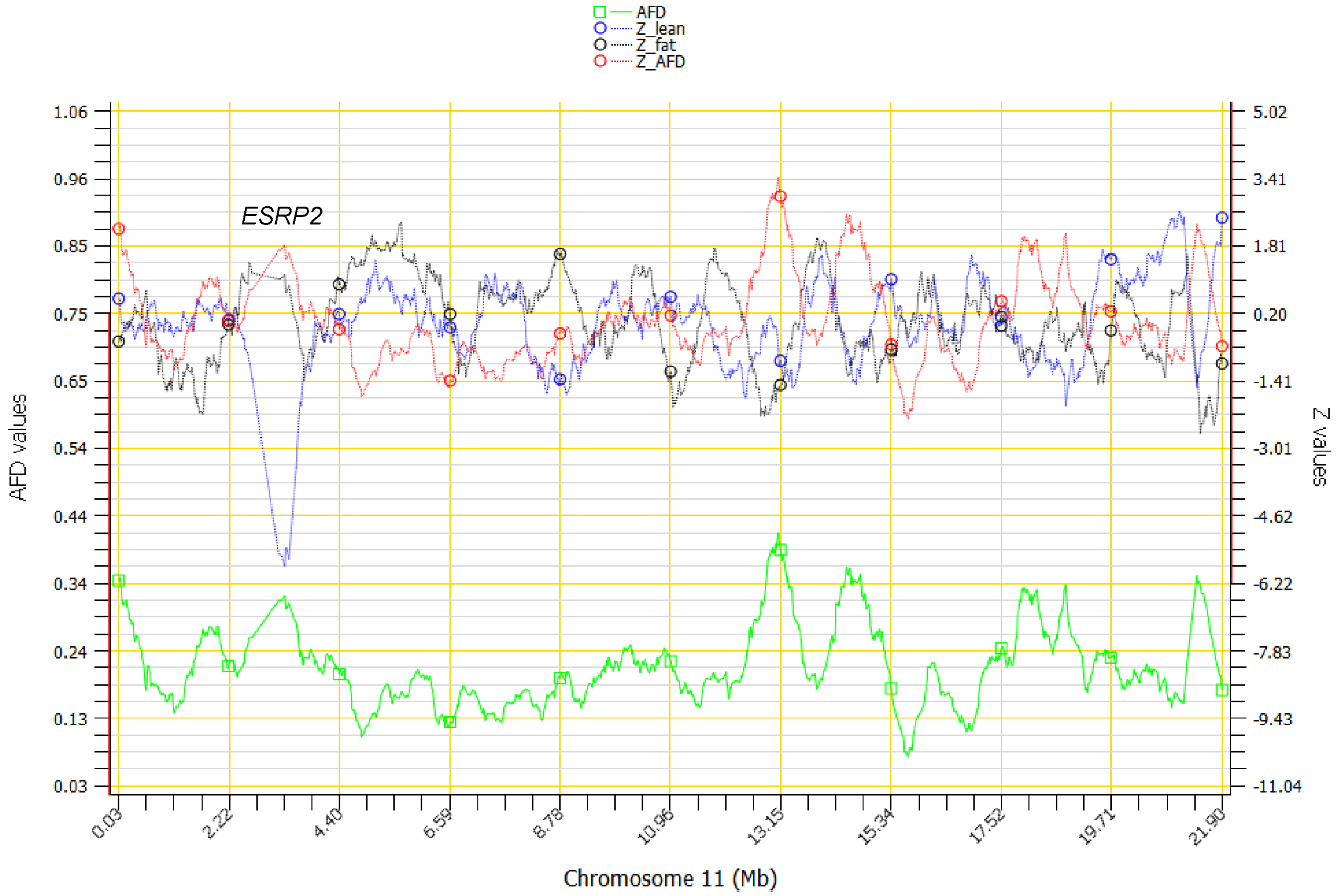
AFD
Z_lean
Z_fat
Z_AFD
ESRP2
AFD values
Z values
1.06
0.96
0.85
0.75
0.65
0.54
0.44
0.34
0.24
0.13
0.03
5.02
3.41
1.81
0.20
-1.41
-3.01
-4.62
-6.22
-7.83
-9.43
-11.04
0.03
2.22
4.40
6.59
8.78
10.96
13.15
15.34
17.52
19.71
21.90
Chromosome 11 (Mb)

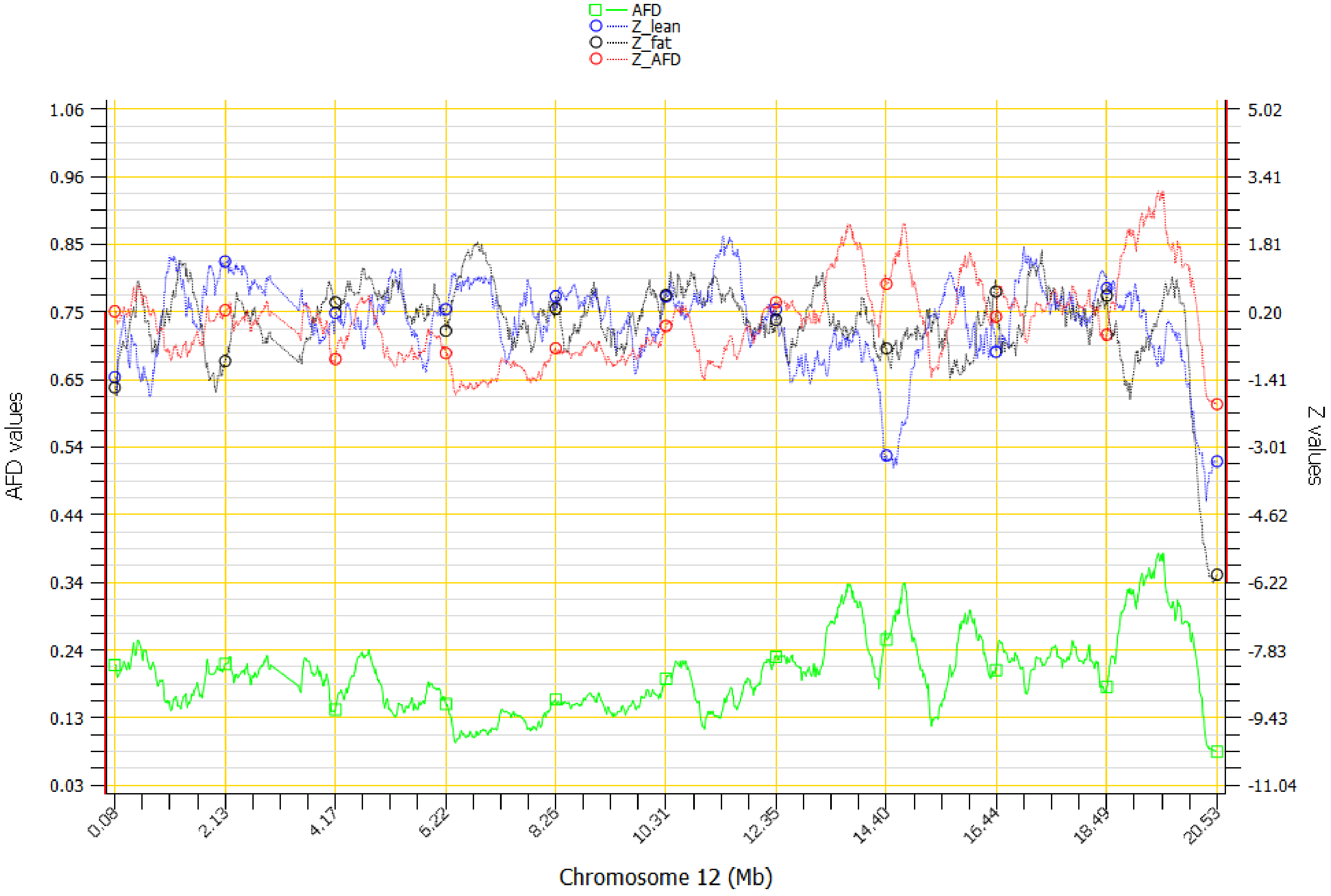
AFD
Z_lean
Z_fat
Z_AFD
AFD values
Z values
1.06
0.96
0.85
0.75
0.65
0.54
0.44
0.34
0.24
0.13
0.03
5.02
3.41
1.81
0.20
-1.41
-3.01
-4.62
-6.22
-7.83
-9.43
-11.04
0.08
2.13
4.17
6.22
8.26
10.31
12.35
14.40
16.44
18.49
20.53
Chromosome 12 (Mb)

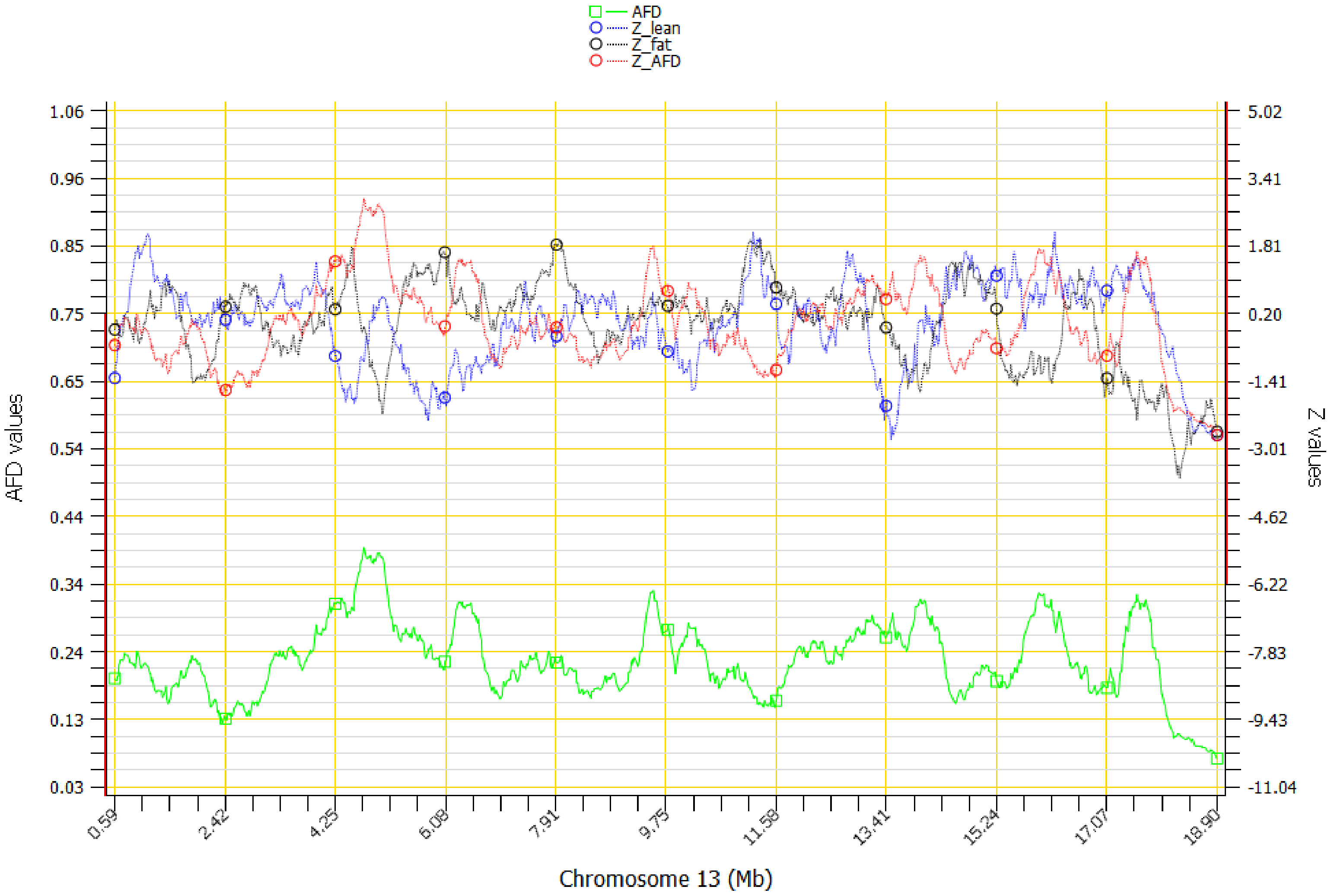

AFD
Z_lean
Z_fat
Z_AFD
AFD values
Z values
1.06
0.96
0.85
0.75
0.65
0.54
0.44
0.34
0.24
0.13
0.03
5.02
3.41
1.81
0.20
-1.41
-3.01
-4.62
-6.22
-7.83
-9.43
-11.04
0.59
2.42
4.25
6.08
7.91
9.75
11.58
13.41
15.24
17.07
18.90
Chromosome 13 (Mb)

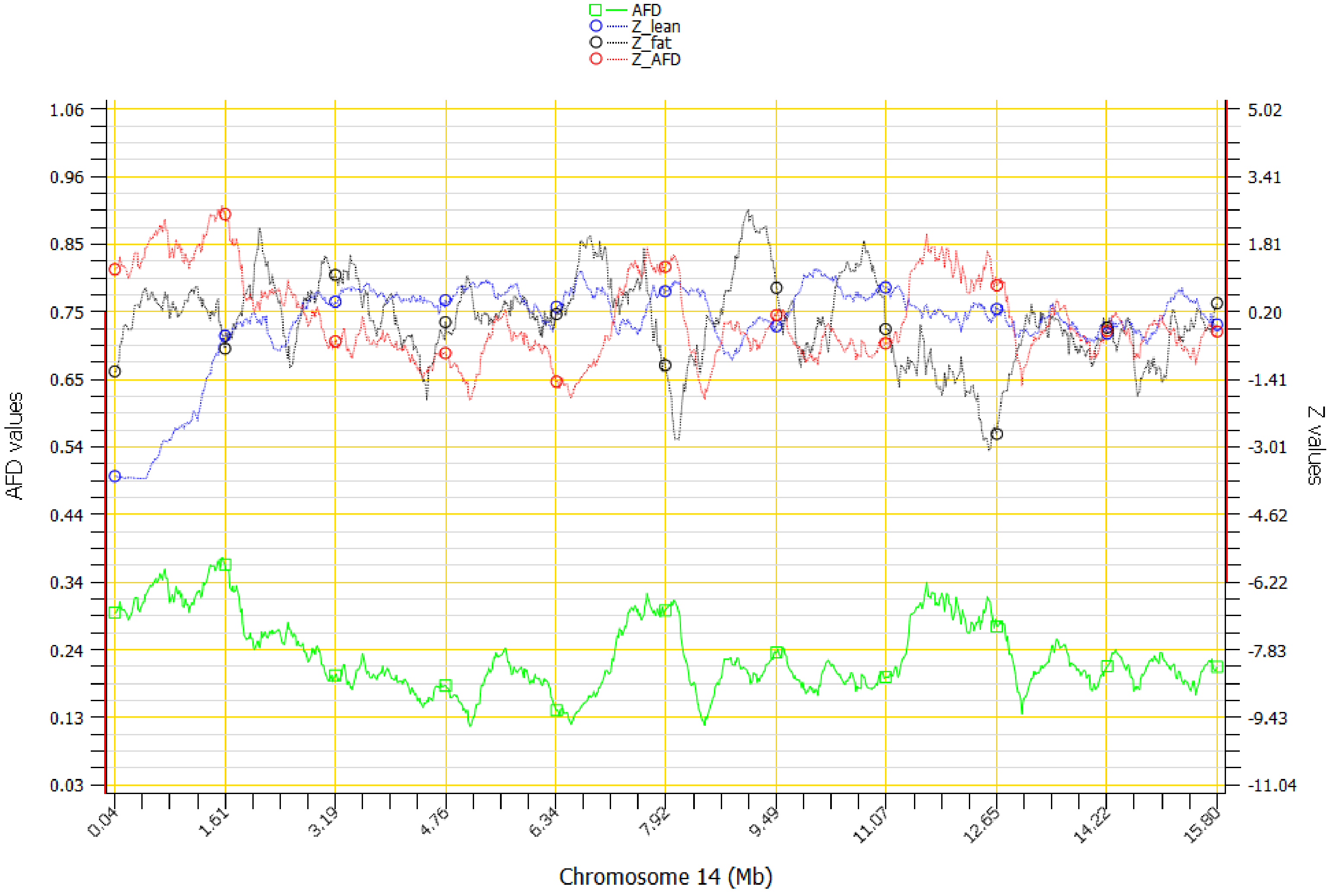

AFD
Z_lean
Z_fat
Z_AFD
AFD values
Z values
1.06
0.96
0.85
0.75
0.65
0.54
0.44
0.34
0.24
0.13
0.03
5.02
3.41
1.81
0.20
-1.41
-3.01
-4.62
-6.22
-7.83
-9.43
-11.04
0.04
1.61
3.19
4.76
6.34
7.92
9.49
11.07
12.65
14.22
15.80
Chromosome 14 (Mb)

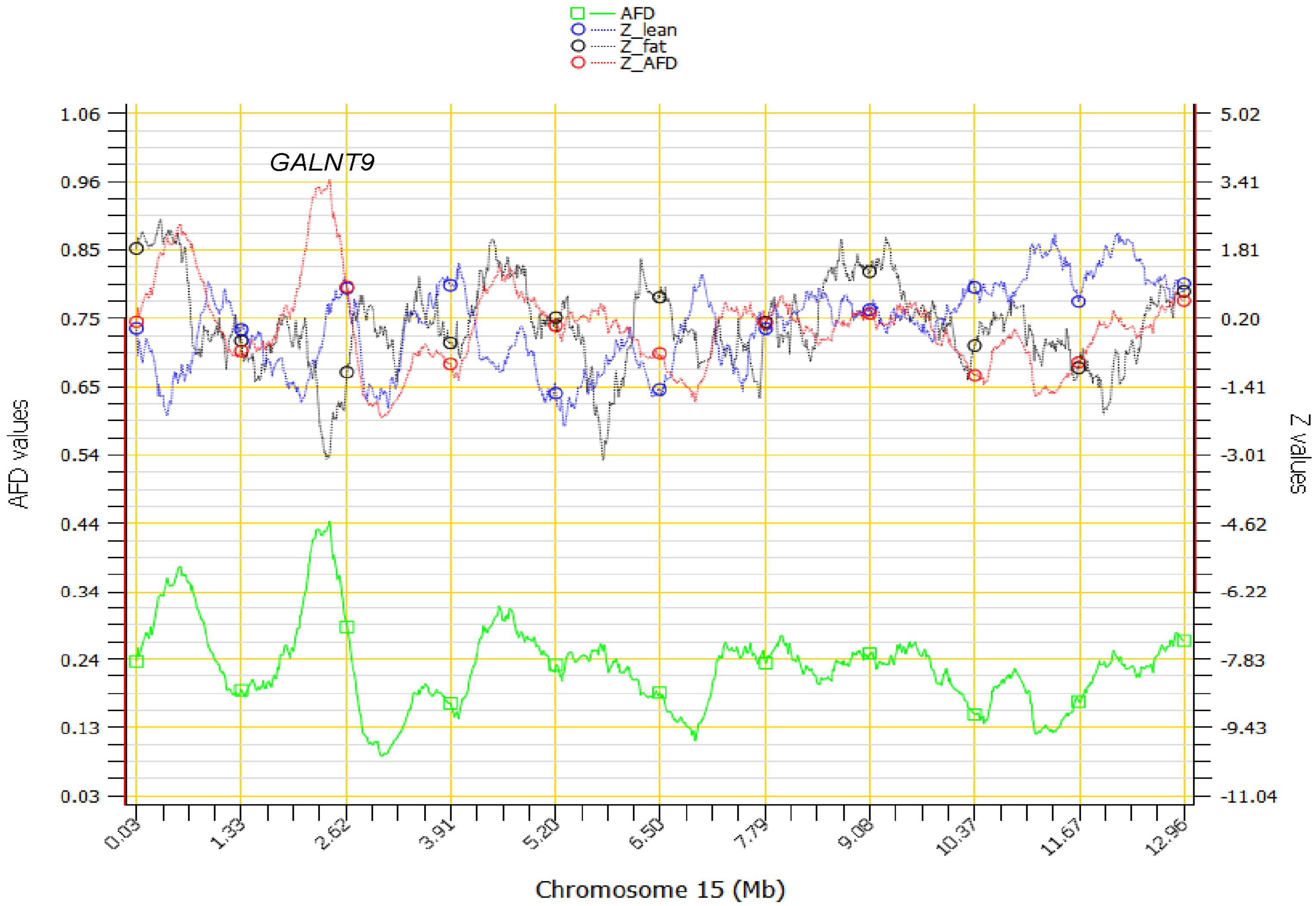

AFD
Z_lean
Z_fat
Z_AFD
GALNT9
AFD values
Z values
1.06
0.96
0.85
0.75
0.65
0.54
0.44
0.34
0.24
0.13
0.03
5.02
3.41
1.81
0.20
-1.41
-3.01
-4.62
-6.22
-7.83
-9.43
-11.04
0.03
1.33
2.62
3.91
5.20
6.50
7.79
9.08
10.37
11.67
12.96
Chromosome 15 (Mb)

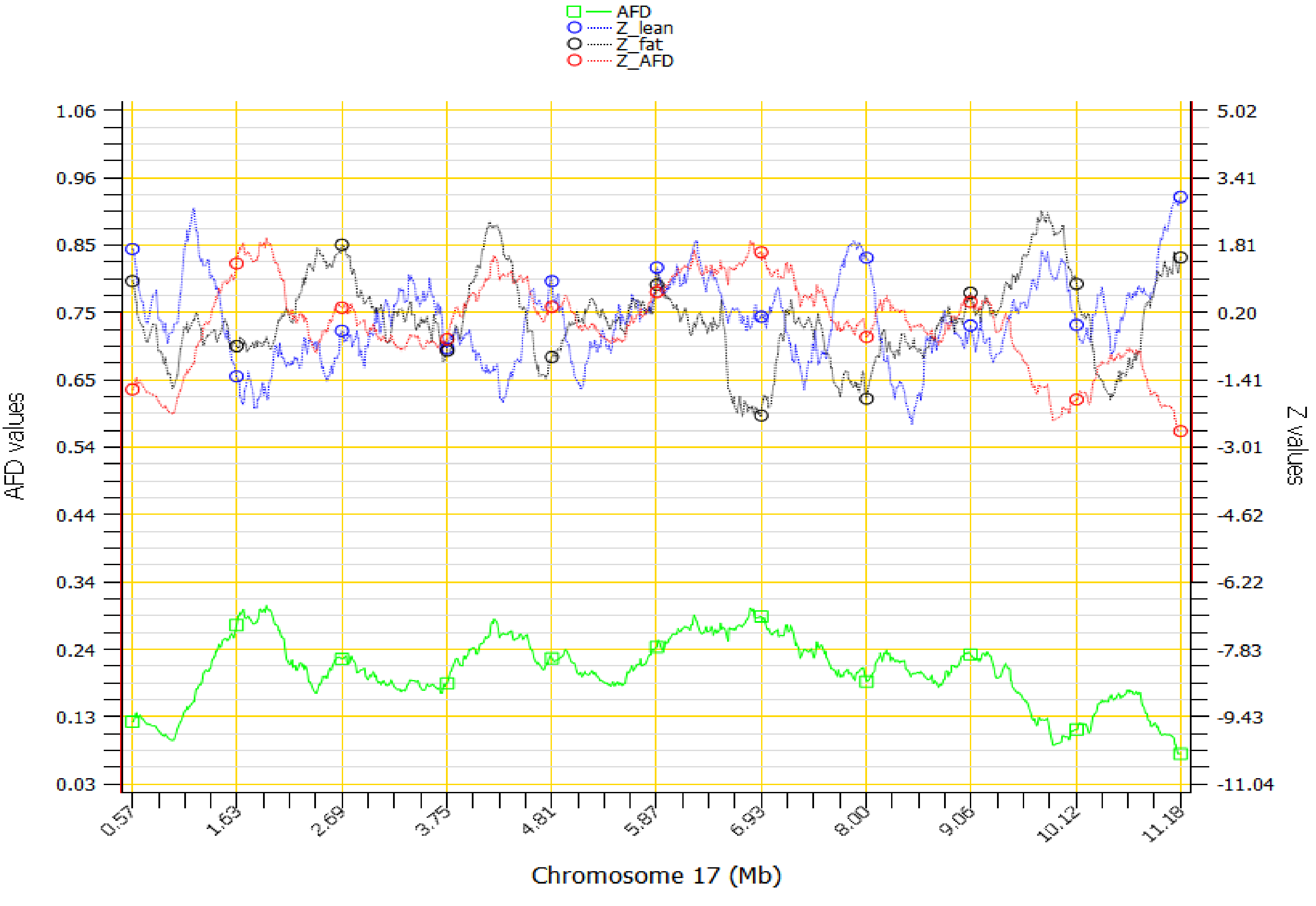

AFD
Z_lean
Z_fat
Z_AFD
AFD values
Z values
1.06
0.96
0.85
0.75
0.65
0.54
0.44
0.34
0.24
0.13
0.03
5.02
3.41
1.81
0.20
-1.41
-3.01
-4.62
-6.22
-7.83
-9.43
-11.04
0.57
1.63
2.69
3.75
4.81
5.87
6.93
8.00
9.06
10.12
11.18
Chromosome 17 (Mb)

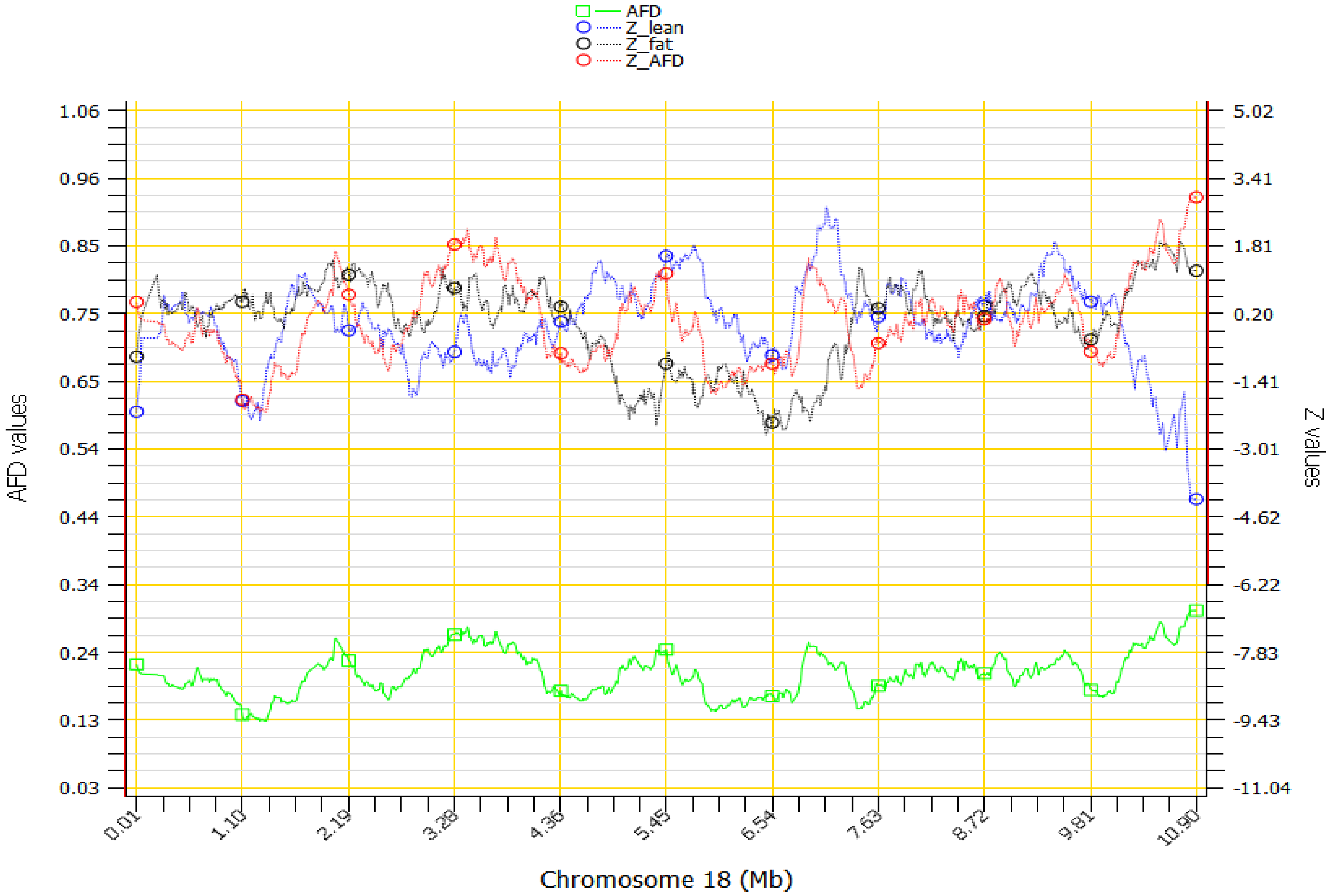
AFD
Z_lean
Z_fat
Z_AFD
AFD values
Z values
1.06
0.96
0.85
0.75
0.65
0.54
0.44
0.34
0.24
0.13
0.03
5.02
3.41
1.81
0.20
-1.41
-3.01
-4.62
-6.22
-7.83
-9.43
-11.04
0.01
1.10
2.19
3.28
4.36
5.45
6.54
7.63
8.72
9.81
10.90
Chromosome 18 (Mb)

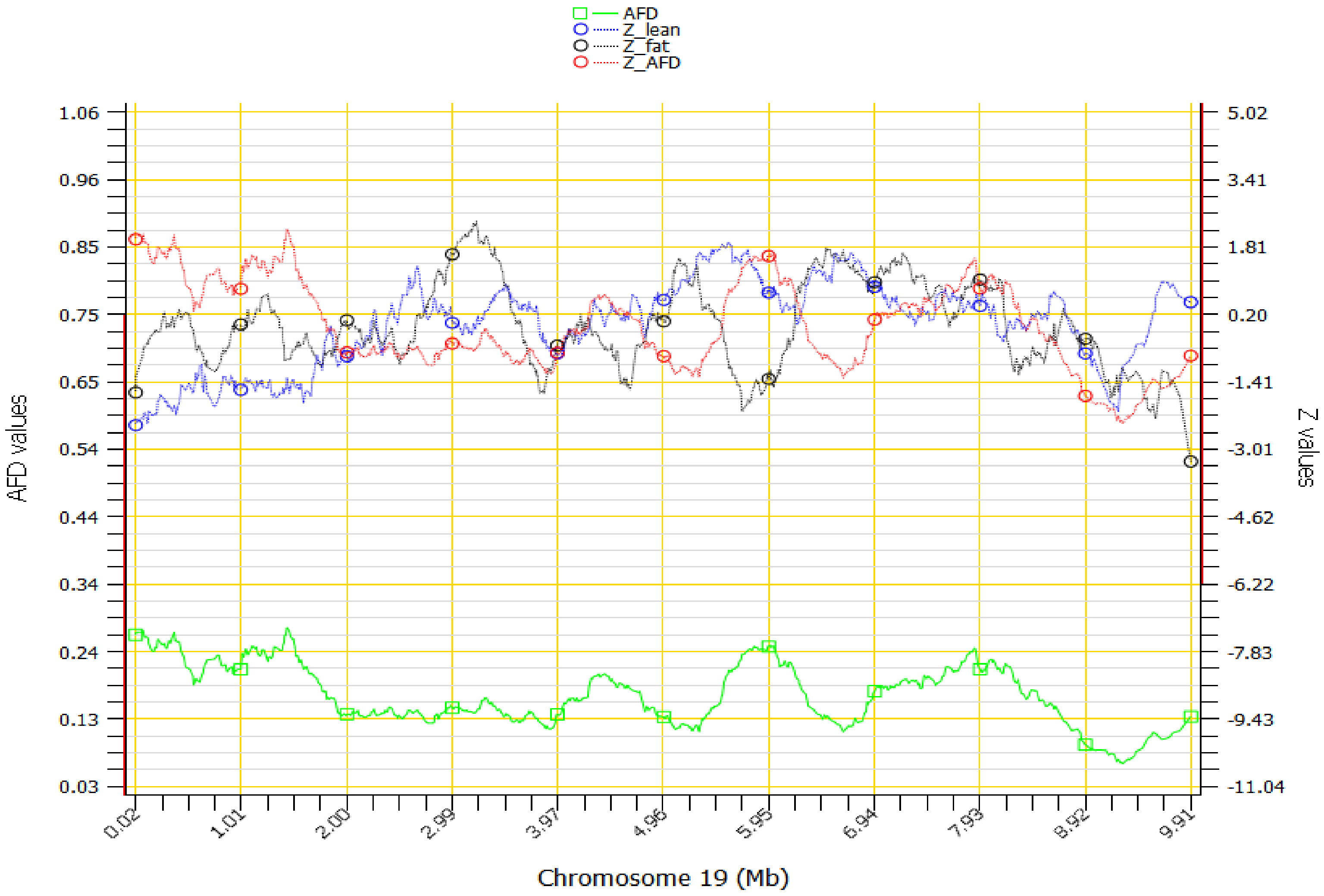
AFD
Z_lean
Z_fat
Z_AFD
AFD values
Z values
1.06
0.96
0.85
0.75
0.65
0.54
0.44
0.34
0.24
0.13
0.03
5.02
3.41
1.81
0.20
-1.41
-3.01
-4.62
-6.22
-7.83
-9.43
-11.04
0.02
1.01
2.00
2.99
3.97
4.96
5.95
6.94
7.93
8.92
9.91
Chromosome 19 (Mb)

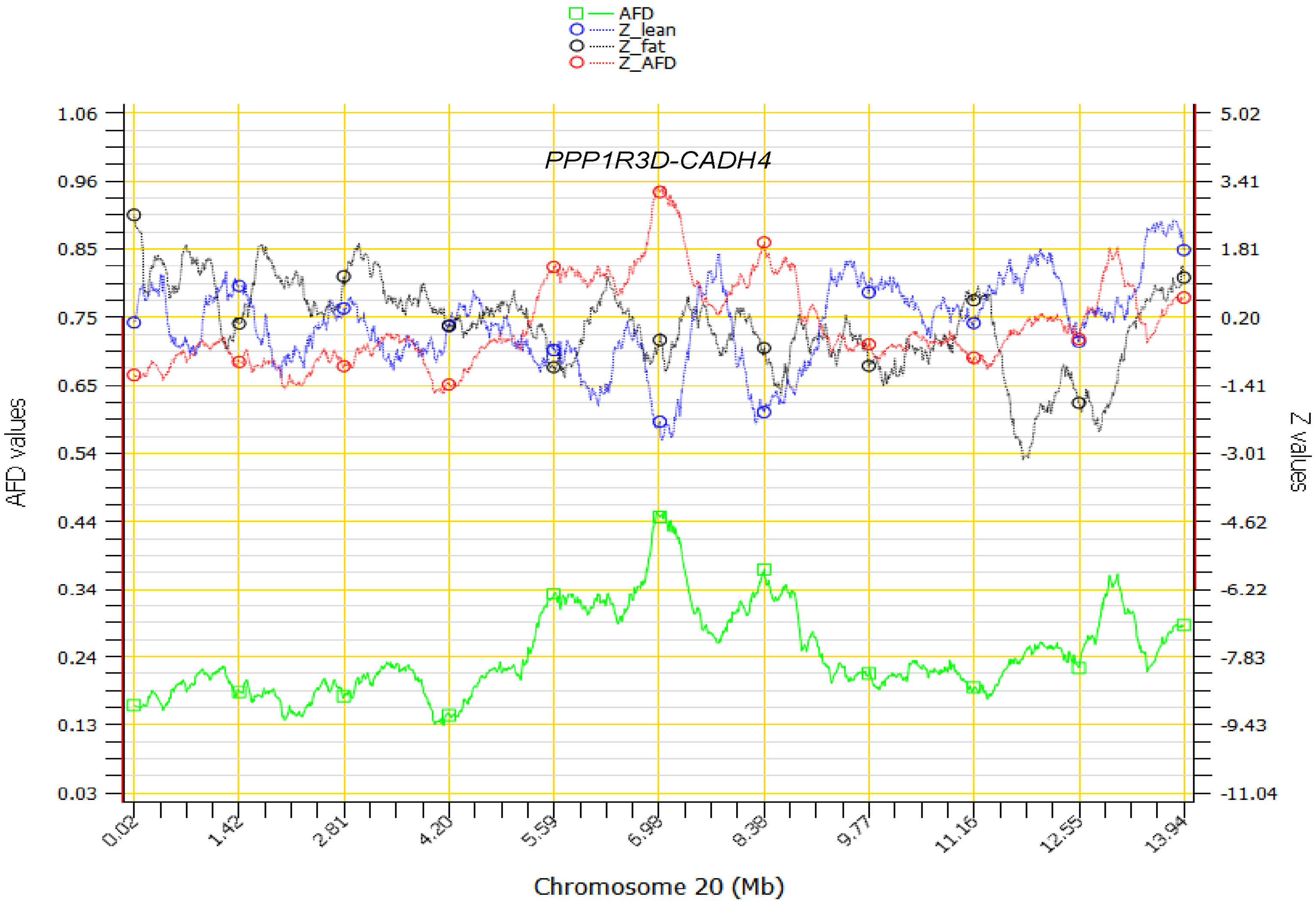
AFD
Z_lean
Z_fat
Z_AFD
PPP1R3D-CADH4
AFD values
Z values
Chromosome 20 (Mb)
1.06
0.96
0.85
0.75
0.65
0.54
0.44
0.34
0.24
0.13
0.03
5.02
3.41
1.81
0.20
-1.41
-3.01
-4.62
-6.22
-7.83
-9.43
-11.04
0.02
1.42
2.81
4.20
5.59
6.98
8.38
9.77
11.16
12.55
13.94

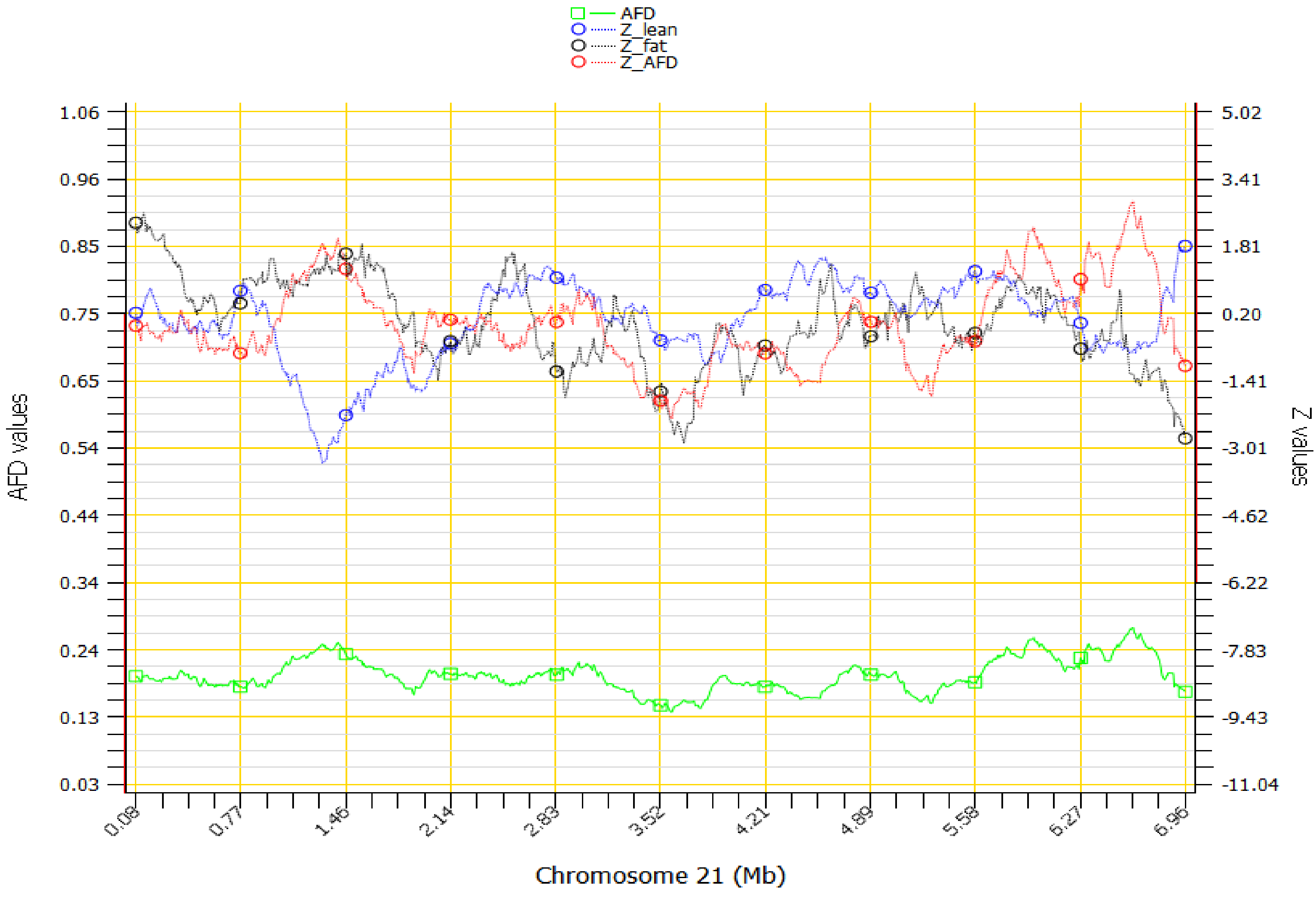
AFD
Z_lean
Z_fat
Z_AFD
AFD values
Z values
Chromosome 21 (Mb)
1.06
0.96
0.85
0.75
0.65
0.54
0.44
0.34
0.24
0.13
0.03
5.02
3.41
1.81
0.20
-1.41
-3.01
-4.62
-6.22
-7.83
-9.43
-11.04
0.08
0.77
1.46
2.14
2.83
3.52
4.21
4.89
5.58
6.27
6.96

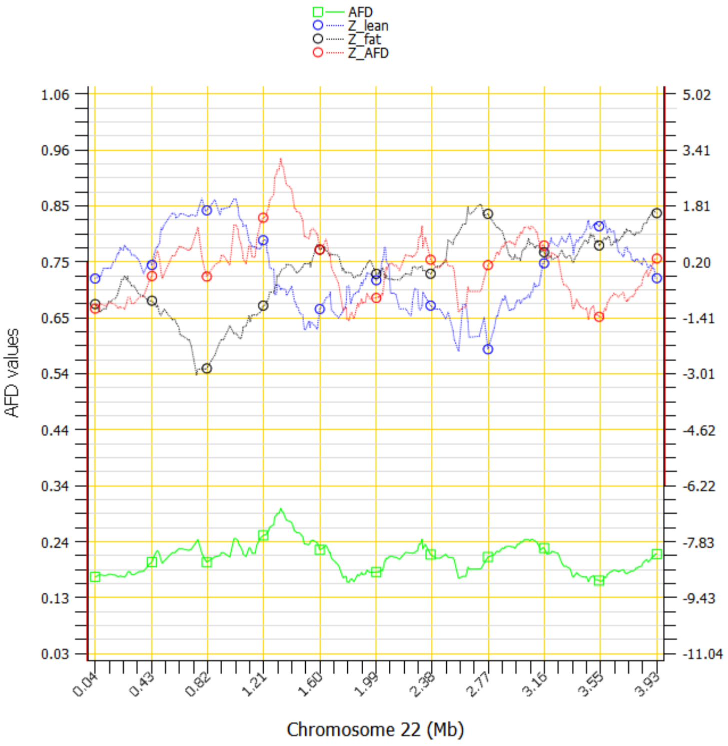
AFD
Z_lean
Z_fat
Z_AFD
AFD values
Z values
1.06
0.96
0.85
0.75
0.65
0.54
0.44
0.34
0.24
0.13
0.03
5.02
3.41
1.81
0.20
-1.41
-3.01
-4.62
-6.22
-7.83
-9.43
-11.04
0.04
0.43
0.82
1.21
1.60
1.99
2.38
2.77
3.16
3.55
3.93
Chromosome 22 (Mb)

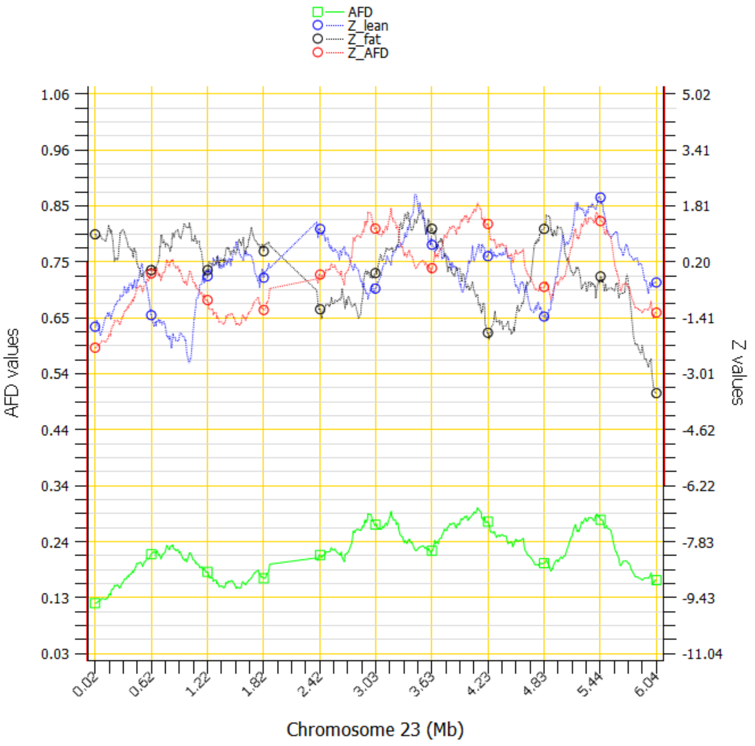
AFD
Z_lean
Z_fat
Z_AFD
AFD values
Z values
1.06
0.96
0.85
0.75
0.65
0.54
0.44
0.34
0.24
0.13
0.03
5.02
3.41
1.81
0.20
-1.41
-3.01
-4.62
-6.22
-7.83
-9.43
-11.04
0.02
0.62
1.22
1.82
2.42
3.03
3.63
4.23
4.83
5.44
6.04
Chromosome 23 (Mb)

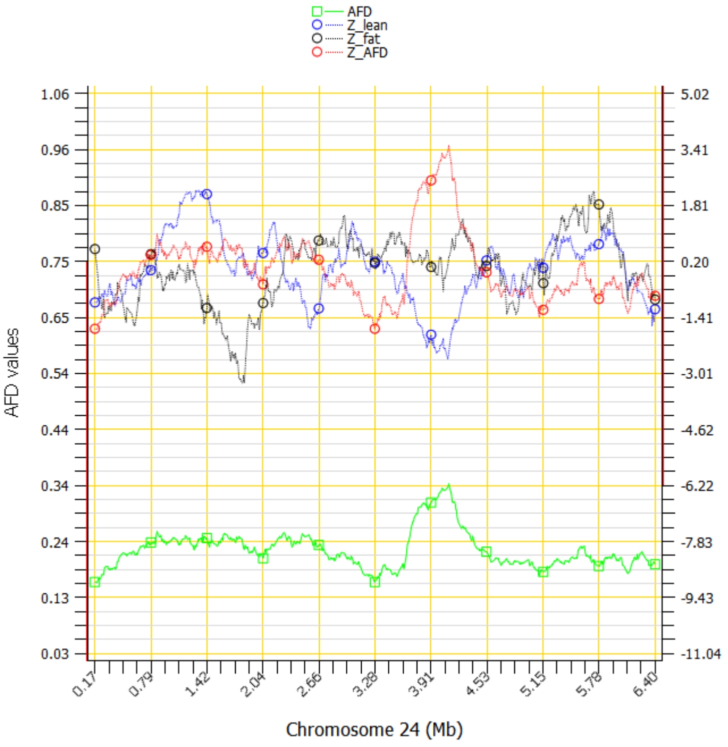
AFD
Z_lean
Z_fat
Z_AFD
AFD values
Z values
1.06
0.96
0.85
0.75
0.65
0.54
0.44
0.34
0.24
0.13
0.03
5.02
3.41
1.81
0.20
-1.41
-3.01
-4.62
-6.22
-7.83
-9.43
-11.04
0.17
0.79
1.42
2.04
2.66
3.28
3.91
4.53
5.15
5.78
6.40
Chromosome 24 (Mb)

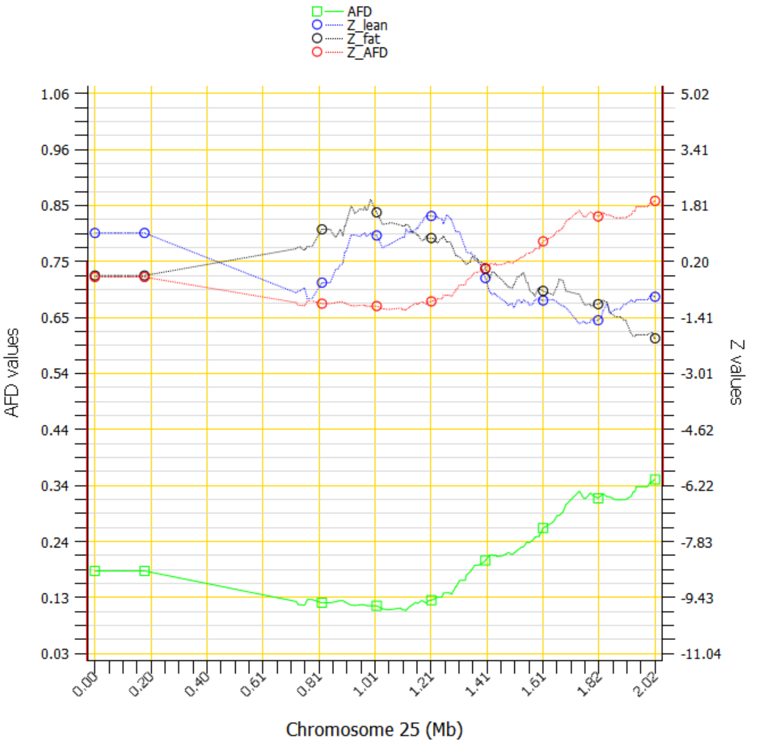
AFD
Z_lean
Z_fat
Z_AFD
AFD values
Z values
1.06
0.96
0.85
0.75
0.65
0.54
0.44
0.34
0.24
0.13
0.03
5.02
3.41
1.81
0.20
-1.41
-3.01
-4.62
-6.22
-7.83
-9.43
-11.04
0.00
0.20
0.40
0.61
0.81
1.01
1.21
1.41
1.61
1.82
2.02
Chromosome 25 (Mb)

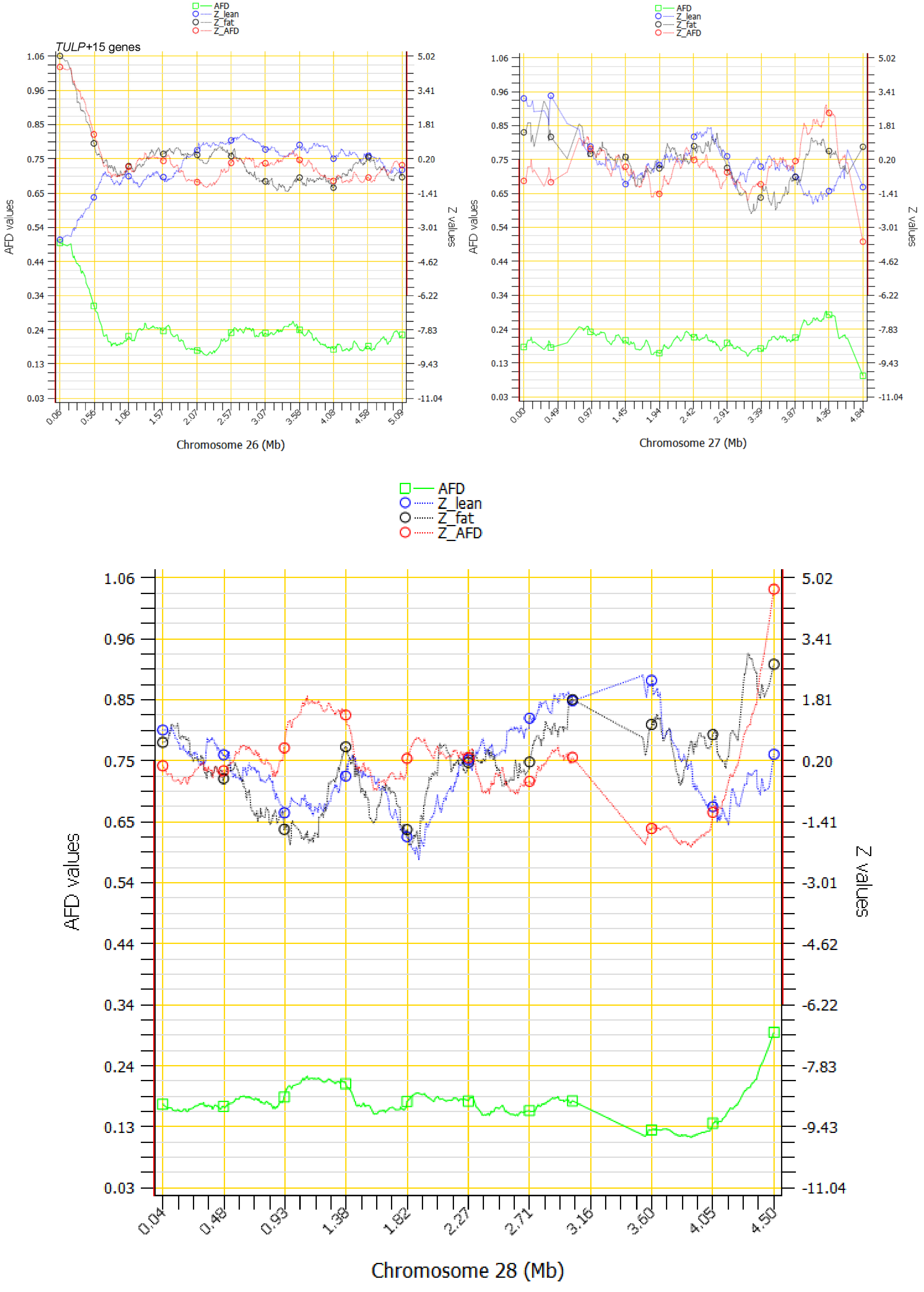
AFD
Z_lean
Z_fat
Z_AFD
TULP+15 genes
AFD values
Z values
1.06
0.96
0.85
0.75
0.65
0.54
0.44
0.34
0.24
0.13
0.03
5.02
3.41
1.81
0.20
-1.41
-3.01
-4.62
-6.22
-7.83
-9.43
-11.04
0.06
0.56
1.06
1.57
2.07
2.57
3.07
3.58
4.08
4.58
5.09
Chromosome 26 (Mb)
0.00
0.49
0.97
1.45
1.94
2.42
2.91
3.39
3.87
4.36
4.84
Chromosome 27 (Mb)
0.04
0.48
0.93
1.38
1.82
2.27
2.71
3.16
3.60
4.05
4.50
Chromosome 28 (Mb)
