# Supplementary material for: Selection Signature Analysis Implicates the PC1/PCSK1 Region for Chicken Abdominal Fat Content
Source: PLoS One. 2012 Jul 11;7(7):e40736. doi: 10.1371/journal.pone.0040736 (PMC3394724; doi:10.1371/journal.pone.0040736)
Supplement: Figure S3 — Linkage disequilibrium patterns of the selection signatures. (PDF) [file pone.0040736.s003.pdf]

A

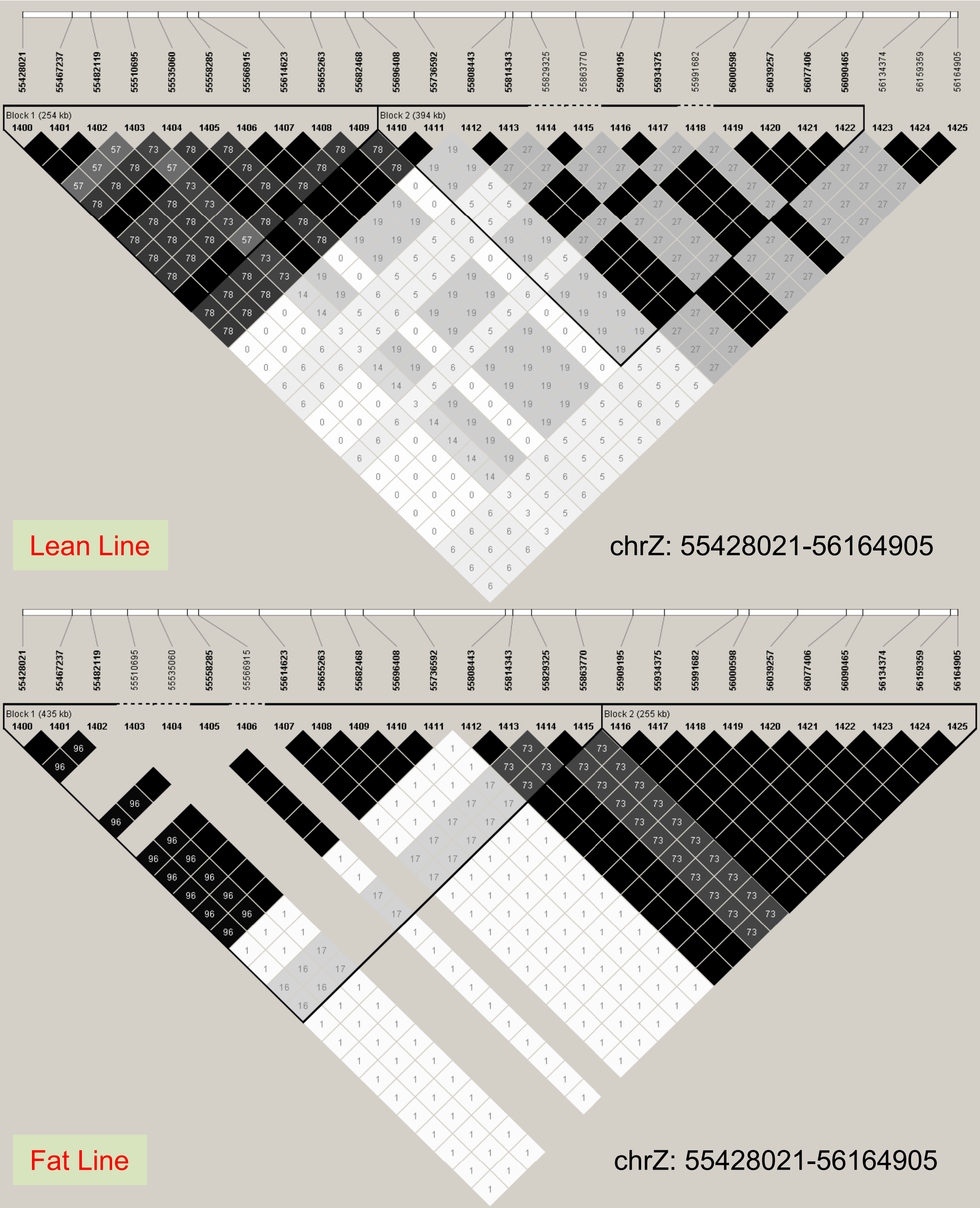
55428021 55467237 55482119 55510695 55535060 55558285 55566915 55614623 55655263 55682468 55696408 55736592 55808443 55814343 55829325 55863770 55909195 55934375 55991682 56000598 56039257 56077406 56090465 56134374 56159359 56164905
Block 1 (254 kb)
Block 2 (394 kb)
1400 1401 1402 1403 1404 1405 1406 1407 1408 1409 1410 1411 1412 1413 1414 1415 1416 1417 1418 1419 1420 1421 1422 1423 1424 1425
Lean Line
chrZ: 55428021-56164905
55428021 55467237 55482119 55510695 55535060 55558285 55566915 55614623 55655263 55682468 55696408 55736592 55808443 55814343 55829325 55863770 55909195 55934375 55991682 56000598 56039257 56077406 56090465 56134374 56159359 56164905
Block 1 (435 kb)
Block 2 (255 kb)
1400 1401 1402 1403 1404 1405 1406 1407 1408 1409 1410 1411 1412 1413 1414 1415 1416 1417 1418 1419 1420 1421 1422 1423 1424 1425
Fat Line
chrZ: 55428021-56164905

B

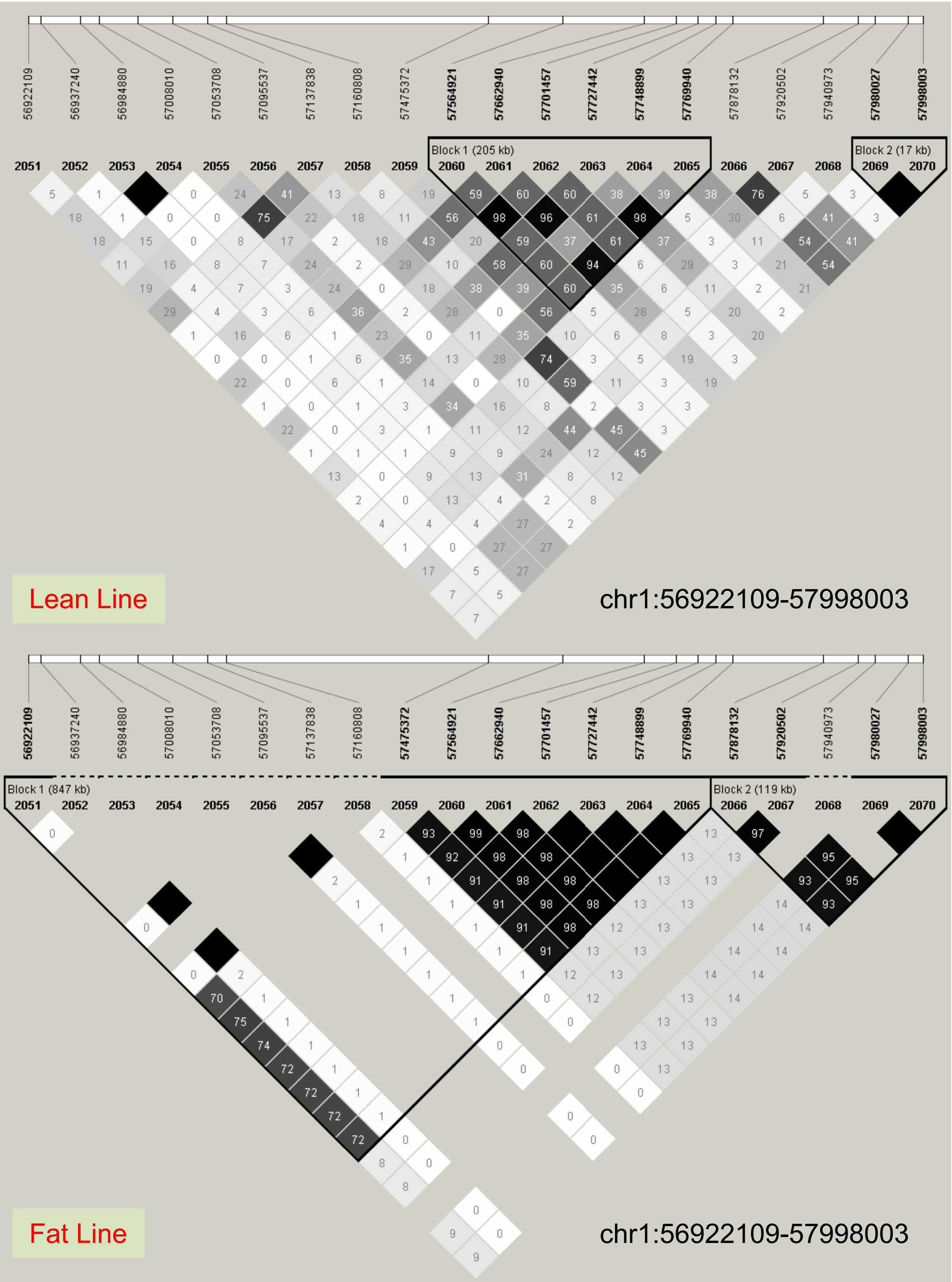
56922109
56937240
56984880
57008010
57053708
57095537
57137838
57160808
57475372
57564921
57662940
57701457
57727442
57748899
57769940
57878132
57920502
57940973
57980027
57998003
Block 1 (205 kb)
Block 2 (17 kb)
2051 2052 2053 2054 2055 2056 2057 2058 2059 2060 2061 2062 2063 2064 2065 2066 2067 2068 2069 2070
Lean Line
chr1:56922109-57998003
Block 1 (847 kb)
Block 2 (119 kb)
Fat Line
chr1:56922109-57998003

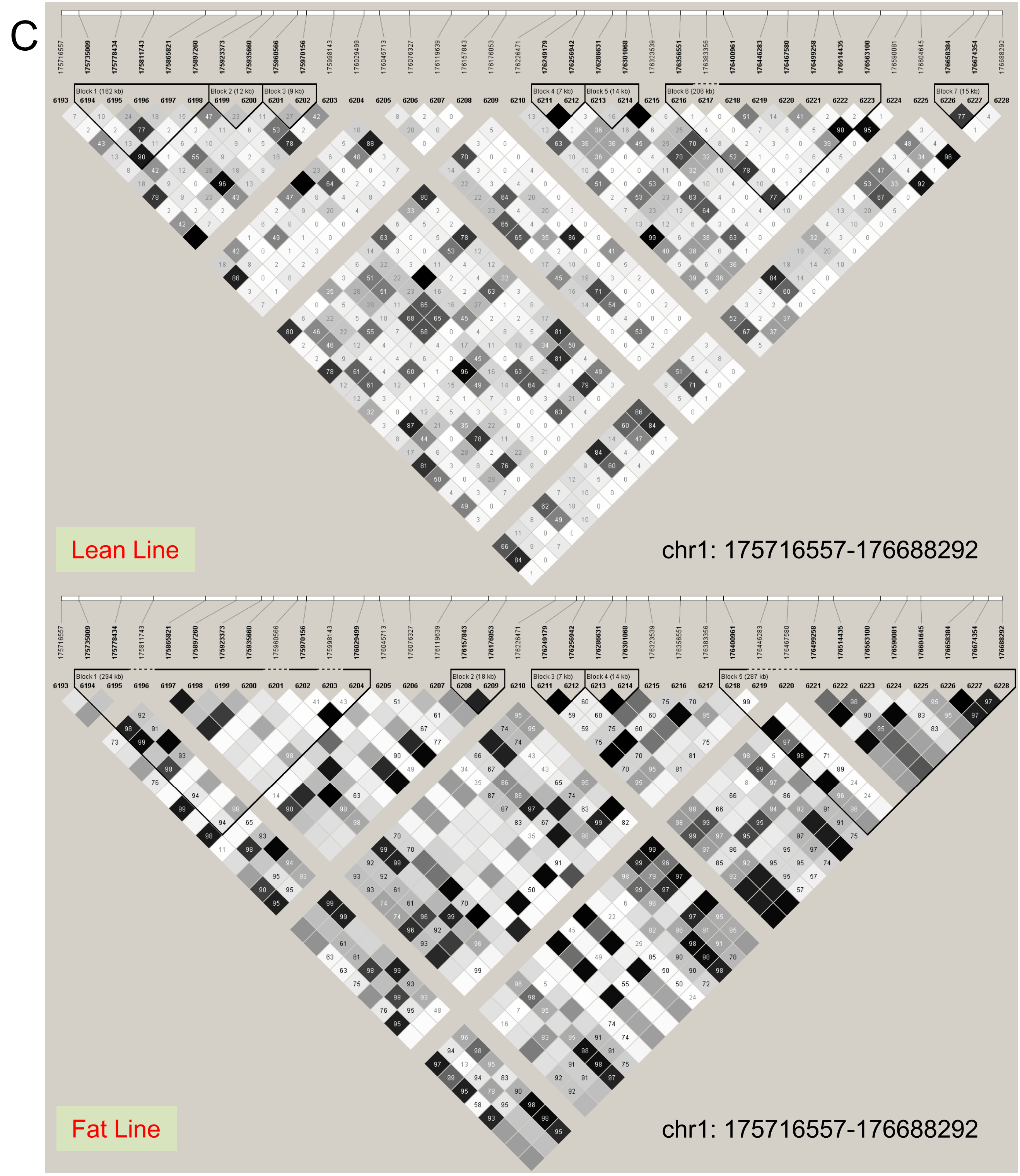
C
Block 1 (162 kb)
Block 2 (12 kb)
Block 3 (9 kb)
Block 4 (7 kb)
Block 5 (14 kb)
Block 6 (206 kb)
Block 7 (15 kb)
Lean Line
chr1: 175716557-176688292
Block 1 (294 kb)
Block 2 (18 kb)
Block 3 (7 kb)
Block 4 (14 kb)
Block 5 (287 kb)
Fat Line
chr1: 175716557-176688292

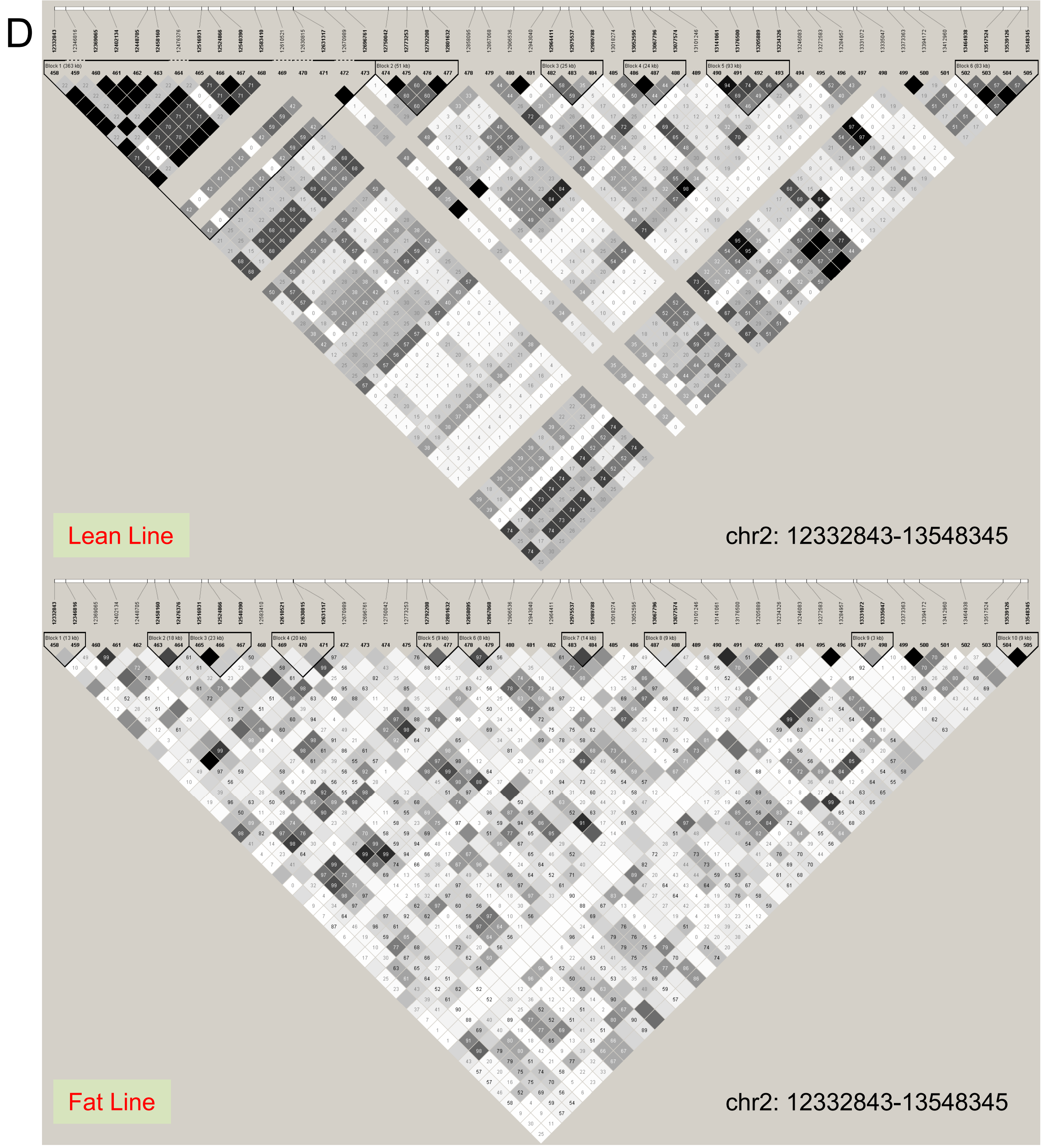
D
Lean Line
chr2: 12332843-13548345
Fat Line
chr2: 12332843-13548345

E

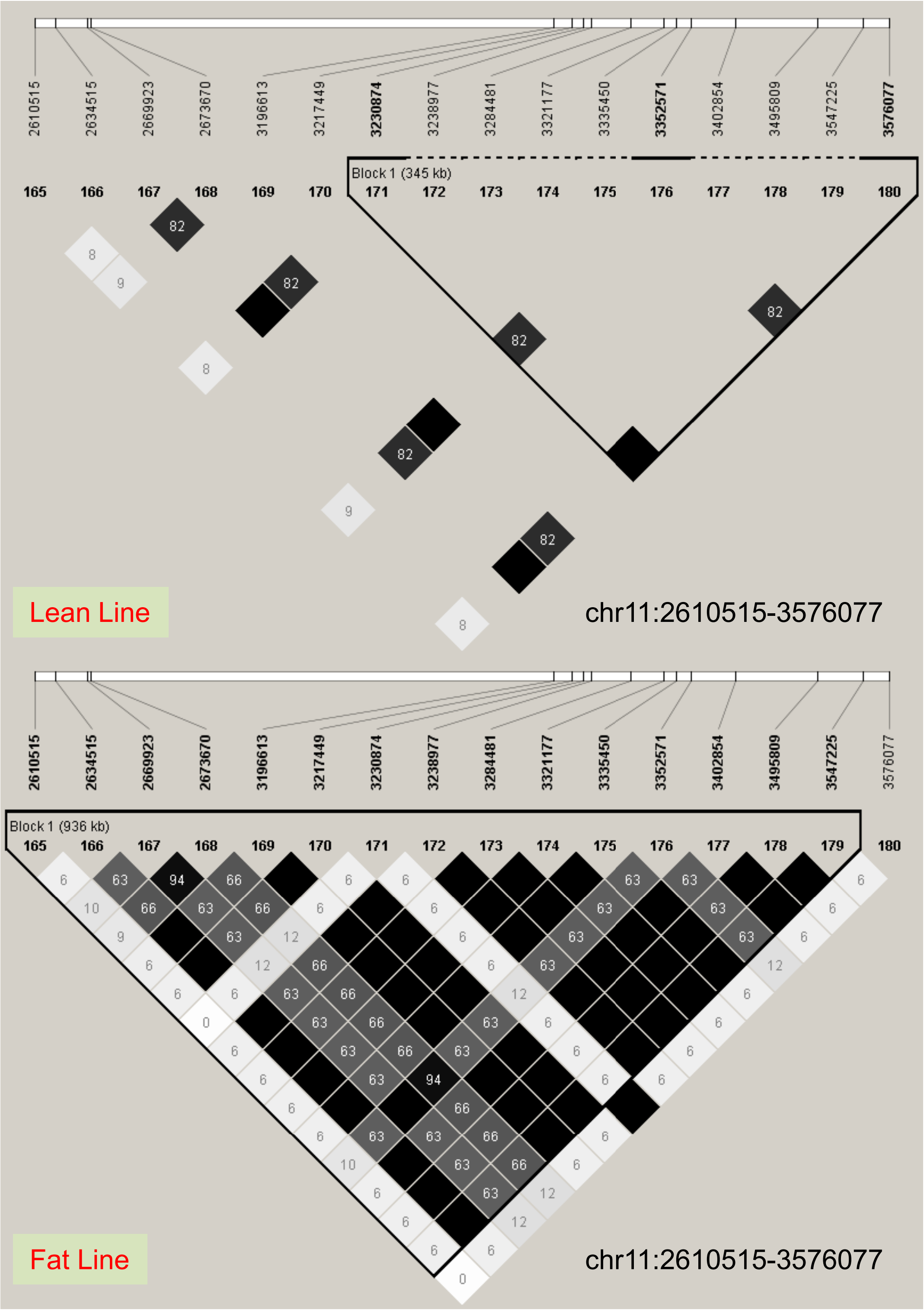

2610515
2634515
2669923
2673670
3196613
3217449
3230874
3238977
3284481
3321177
3335450
3352571
3402854
3495809
3547225
3576077
Block 1 (345 kb)
165 166 167 168 169 170 171 172 173 174 175 176 177 178 179 180
Lean Line
chr11:2610515-3576077
Block 1 (936 kb)
Fat Line
chr11:2610515-3576077

F

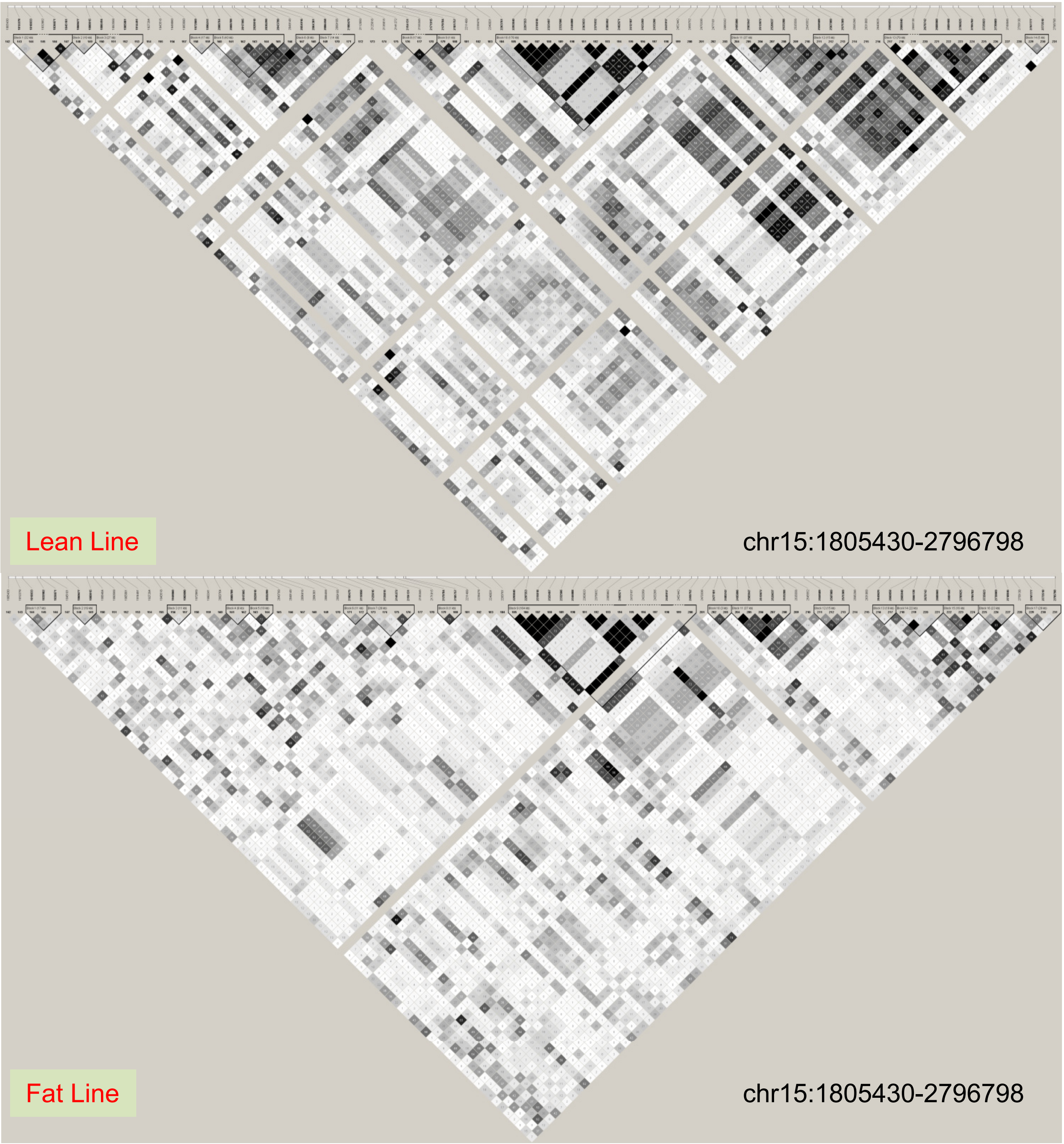

Lean Line
chr15:1805430-2796798
Fat Line
chr15:1805430-2796798

G

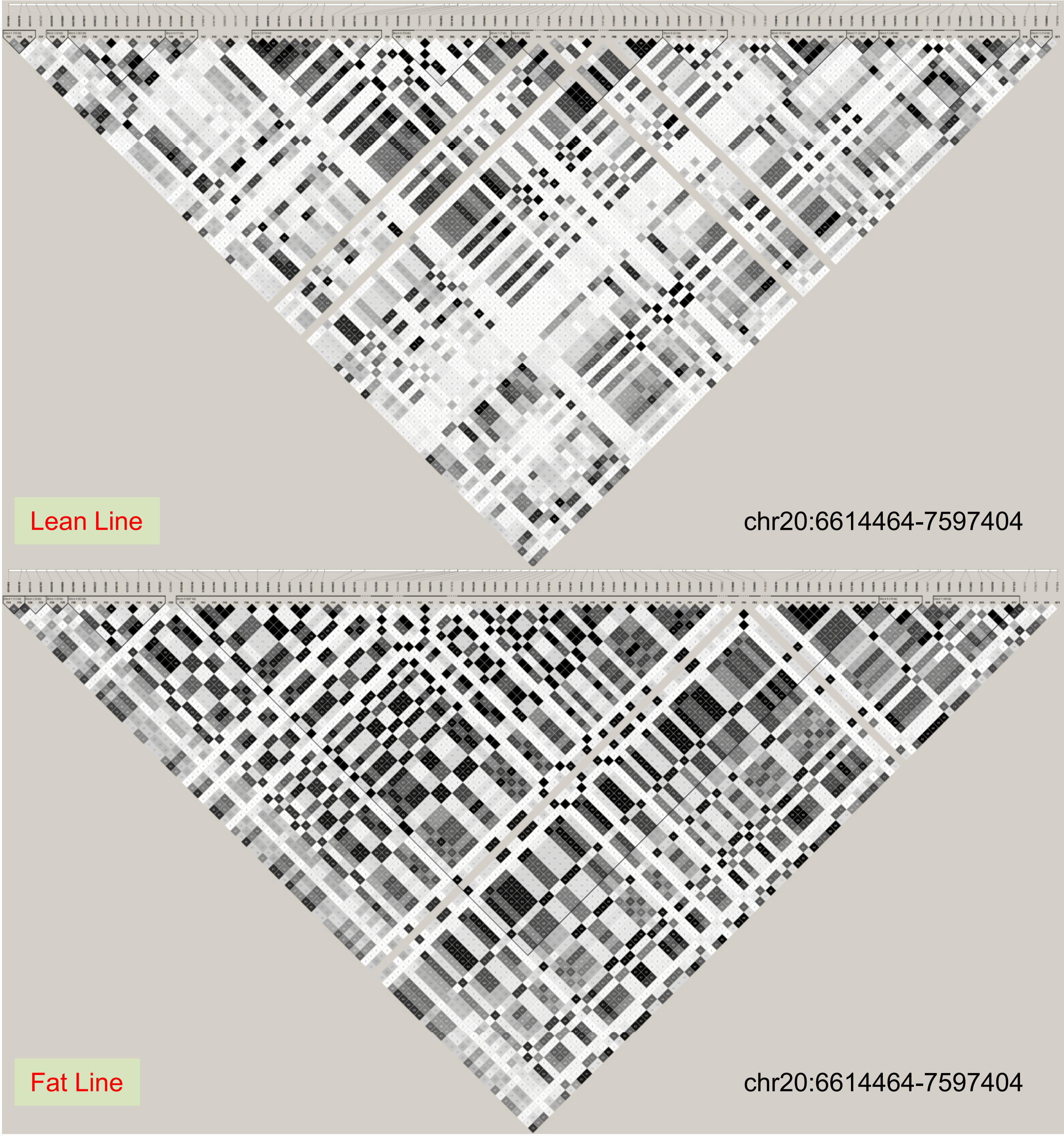
Lean Line
chr20:6614464-7597404
Fat Line
chr20:6614464-7597404

H

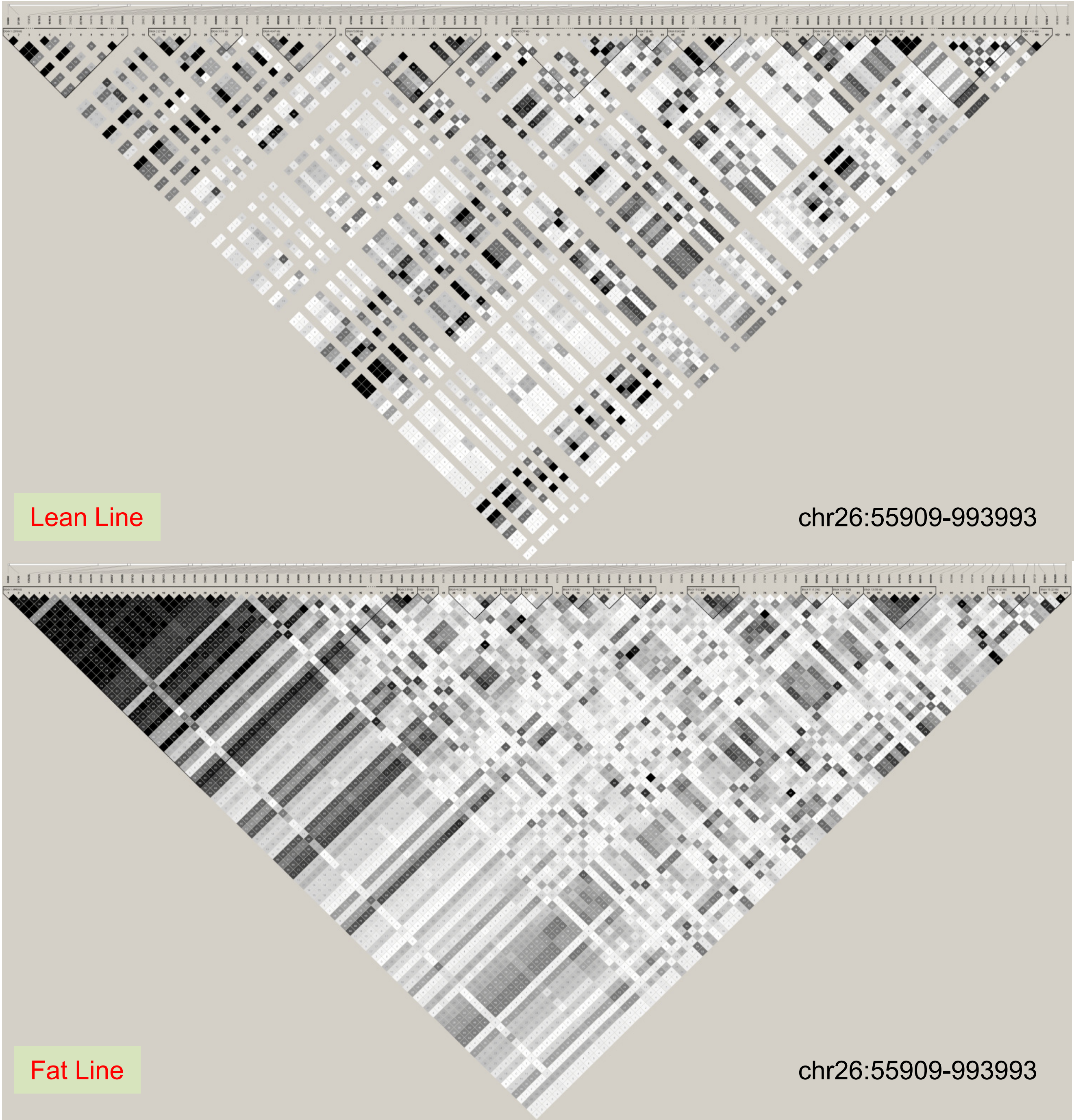

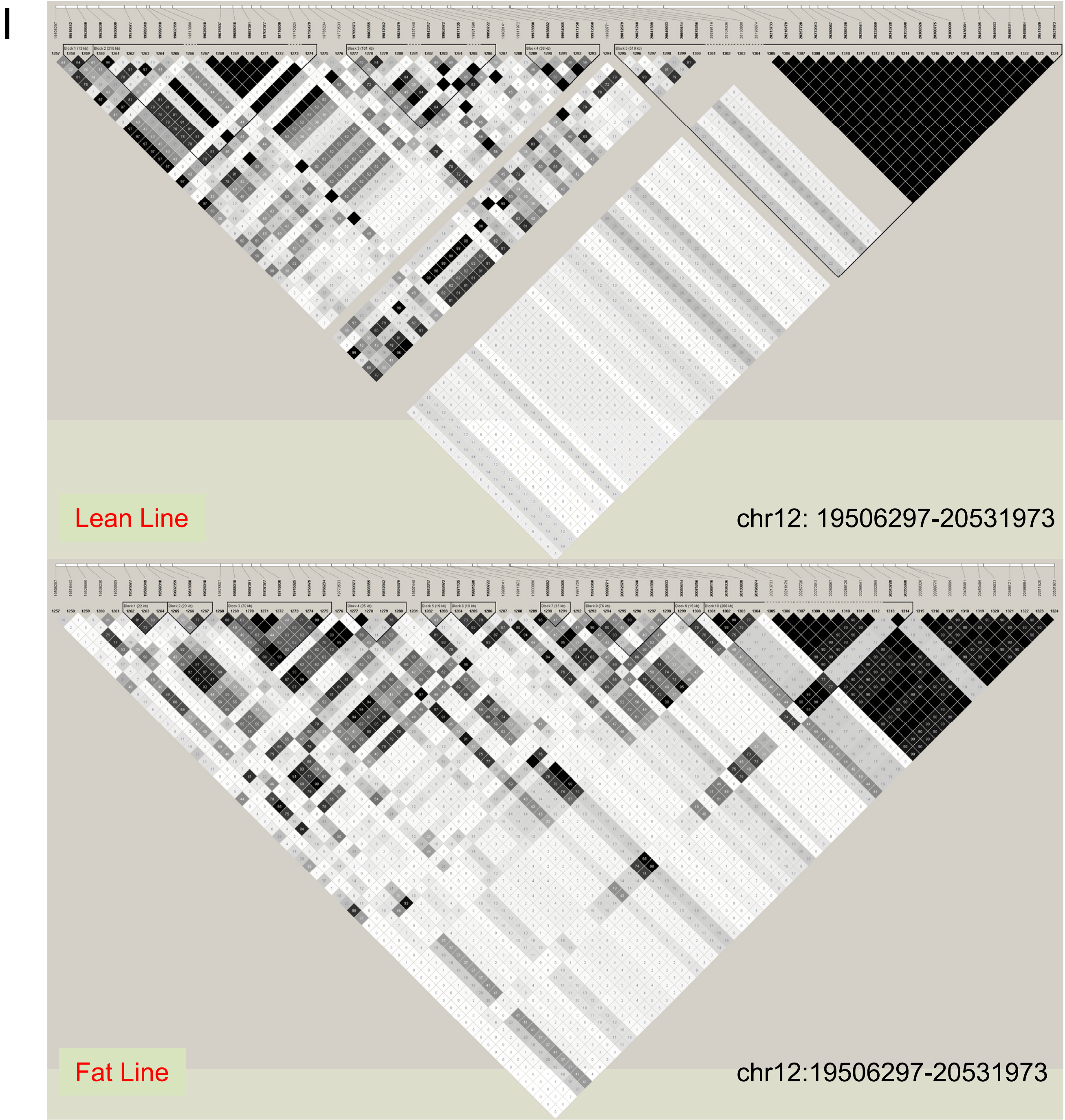
I
Lean Line
chr12: 19506297-20531973
Fat Line
chr12:19506297-20531973

J

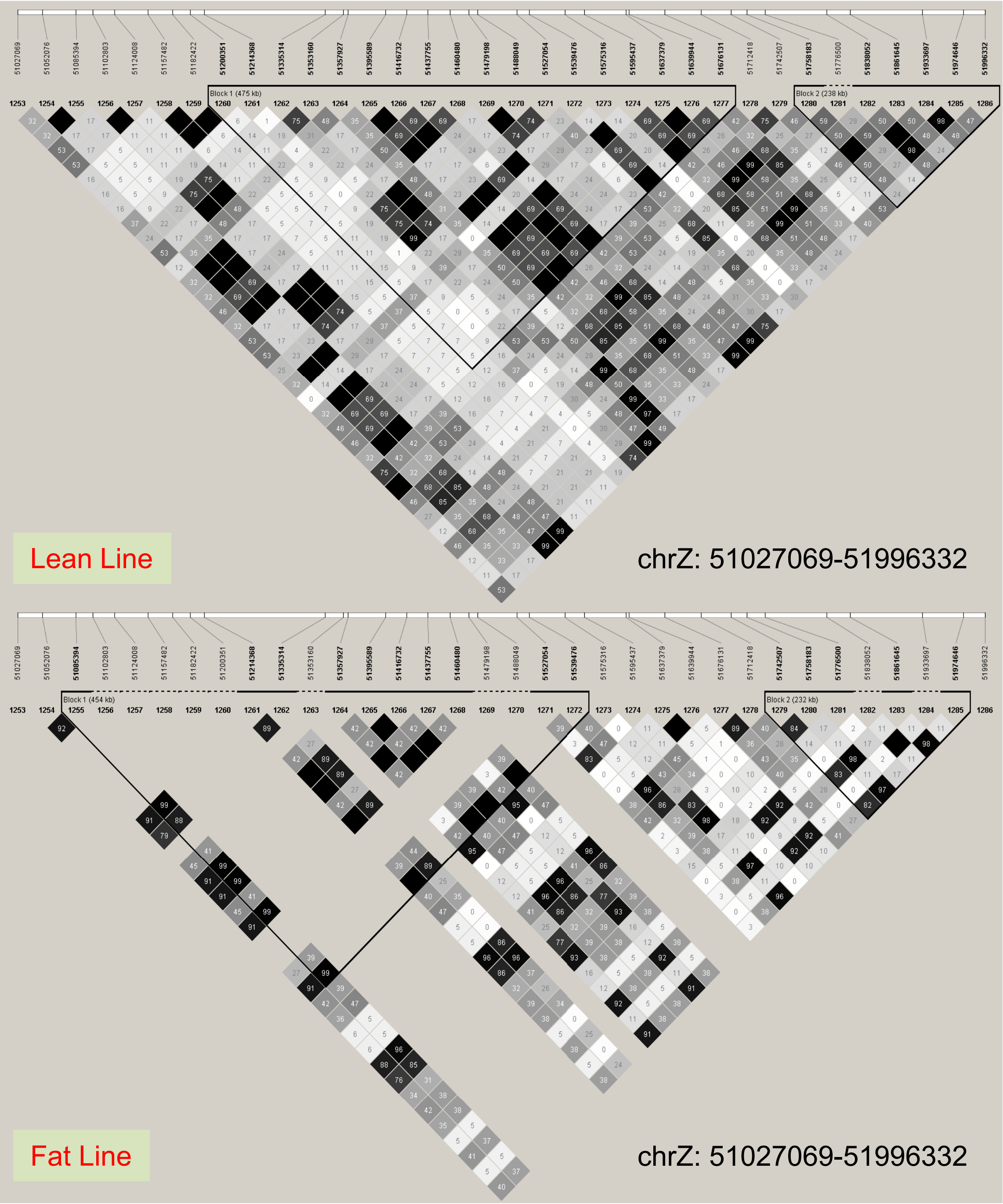
Block 1 (475 kb)
Block 2 (238 kb)
Lean Line
chrZ: 51027069-51996332
Block 1 (454 kb)
Block 2 (232 kb)
Fat Line
chrZ: 51027069-51996332

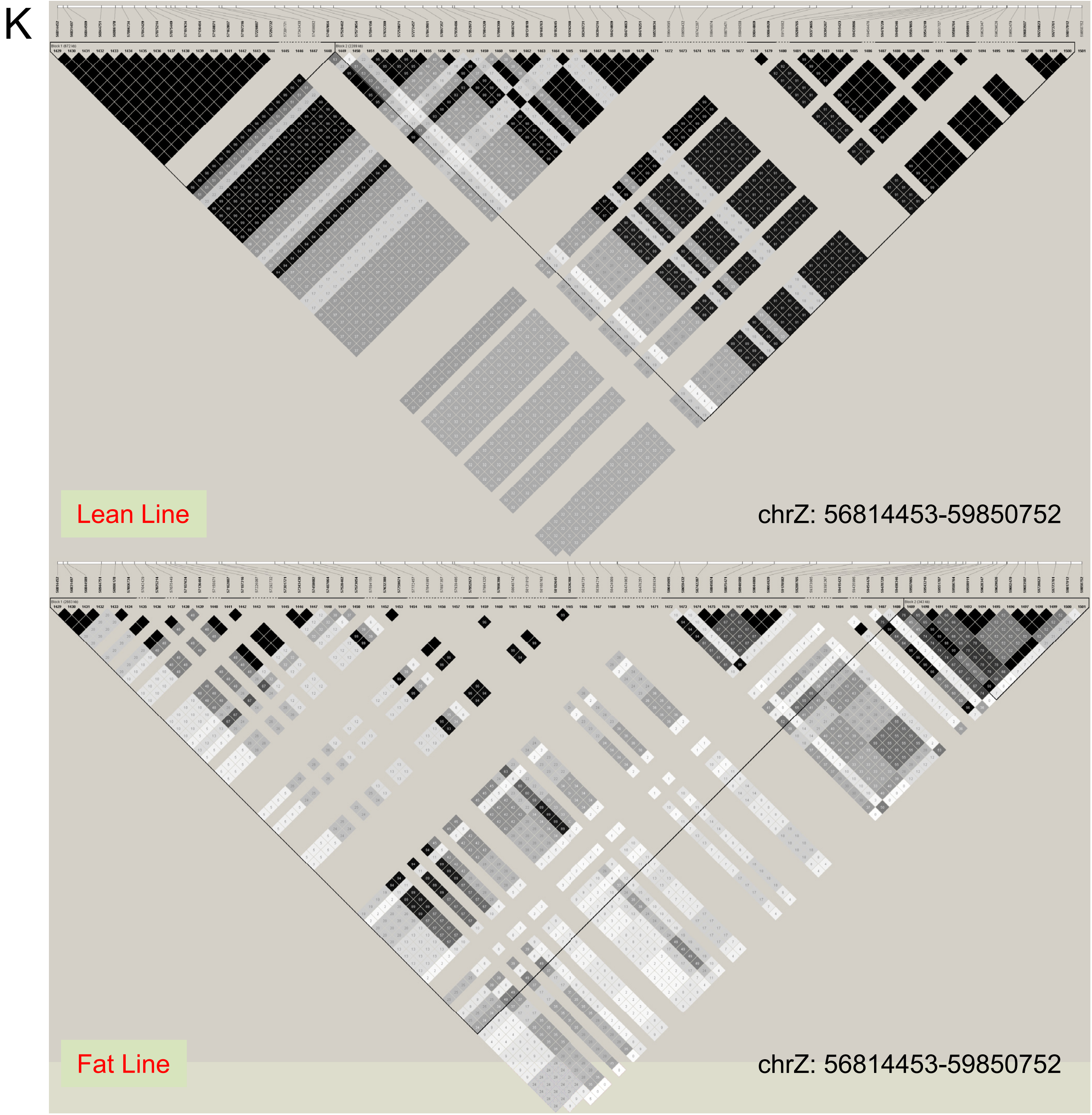

K
Lean Line
chrZ: 56814453-59850752
Fat Line
chrZ: 56814453-59850752
