# Supplementary material for: Selection Signature Analysis Implicates the PC1/PCSK1 Region for Chicken Abdominal Fat Content
Source: PLoS One. 2012 Jul 11;7(7):e40736. doi: 10.1371/journal.pone.0040736 (PMC3394724; doi:10.1371/journal.pone.0040736)
Supplement: Figure S4 — Extended haplotype homozygosity in selection signatures. (PDF) [file pone.0040736.s004.pdf]

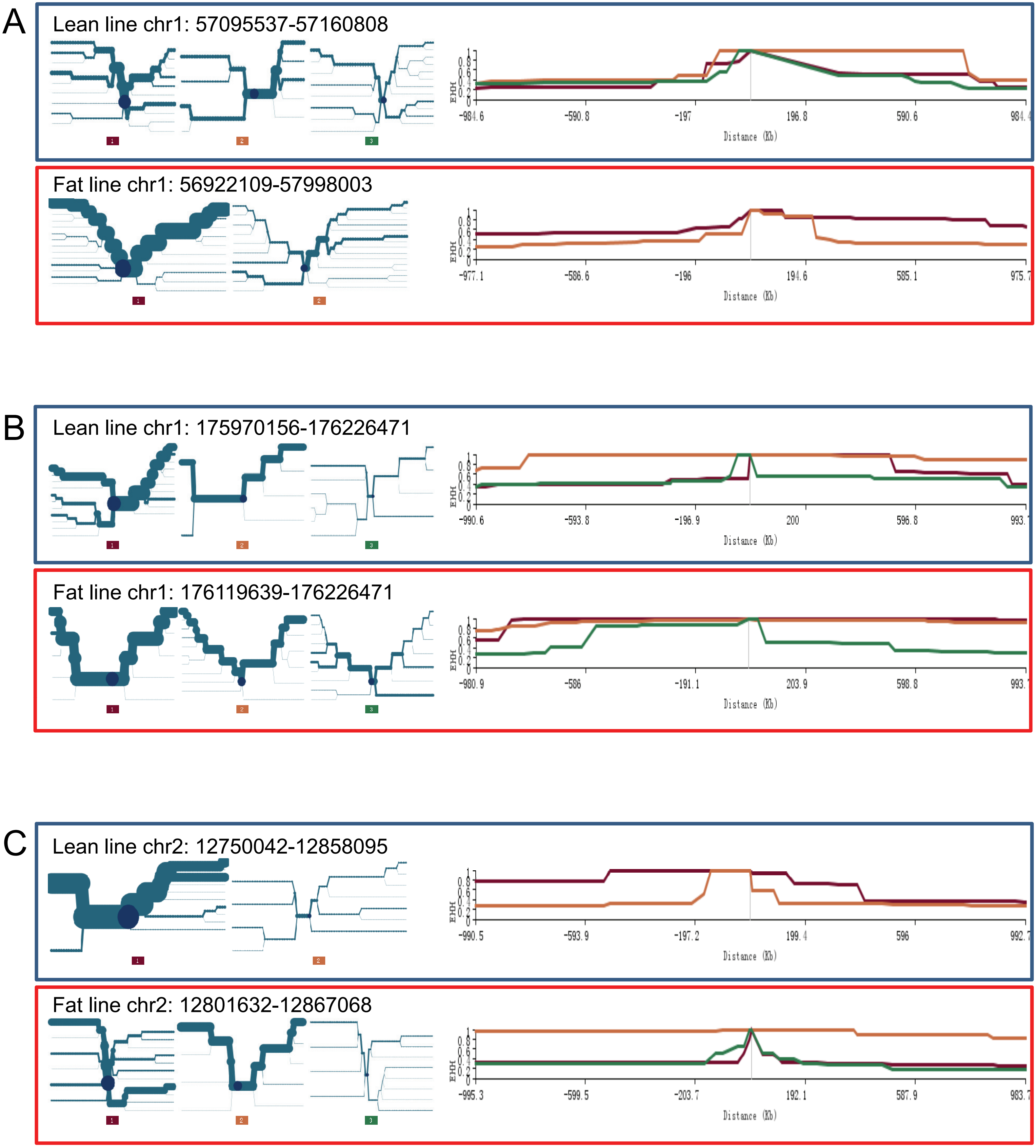
A
Lean line chr1: 57095537-57160808
1
2
3
EHH
-984.6
-590.8
-197
196.8
590.6
Distance (Kb)
Fat line chr1: 56922109-57998003
1
2
EHH
-977.1
-586.6
-196
194.6
585.1
975.7
Distance (Kb)
B
Lean line chr1: 175970156-176226471
1
2
3
EHH
-990.6
-593.8
-196.9
200
596.8
Distance (Kb)
Fat line chr1: 176119639-176226471
1
2
3
EHH
-980.9
-586
-191.1
203.9
598.8
Distance (Kb)
C
Lean line chr2: 12750042-12858095
1
2
EHH
-990.5
-593.9
-197.2
199.4
596
Distance (Kb)
Fat line chr2: 12801632-12867068
1
2
3
EHH
-995.3
-599.5
-203.7
192.1
587.9
Distance (Kb)

D

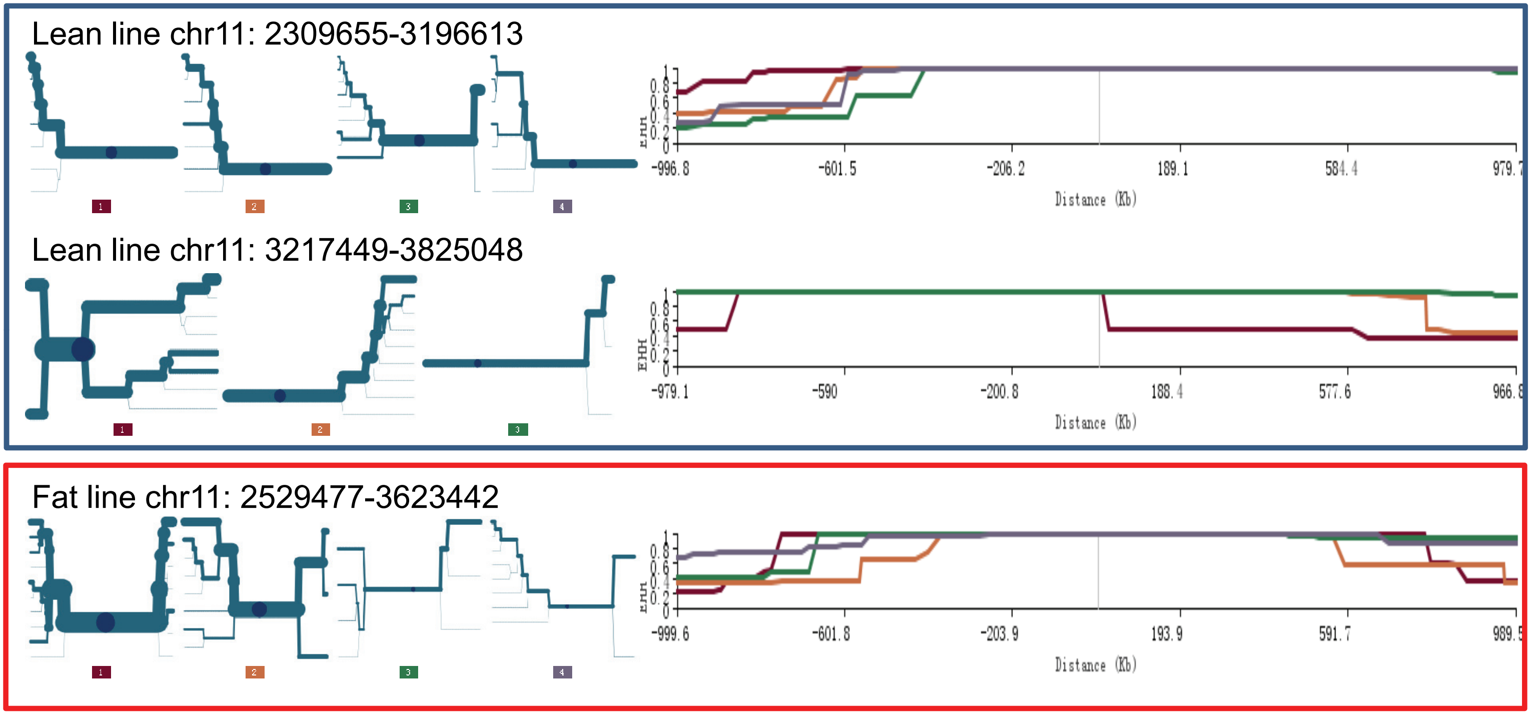
Lean line chr11: 2309655-3196613
1
2
3
4
-996.8
-601.5
-206.2
189.1
584.4
979.7
Distance (Kb)
Lean line chr11: 3217449-3825048
1
2
3
EHH
-979.1
-590
-200.8
188.4
577.6
966.8
Distance (Kb)
Fat line chr11: 2529477-3623442
1
2
3
4
-999.6
-601.8
-203.9
193.9
591.7
989.5
Distance (Kb)

E

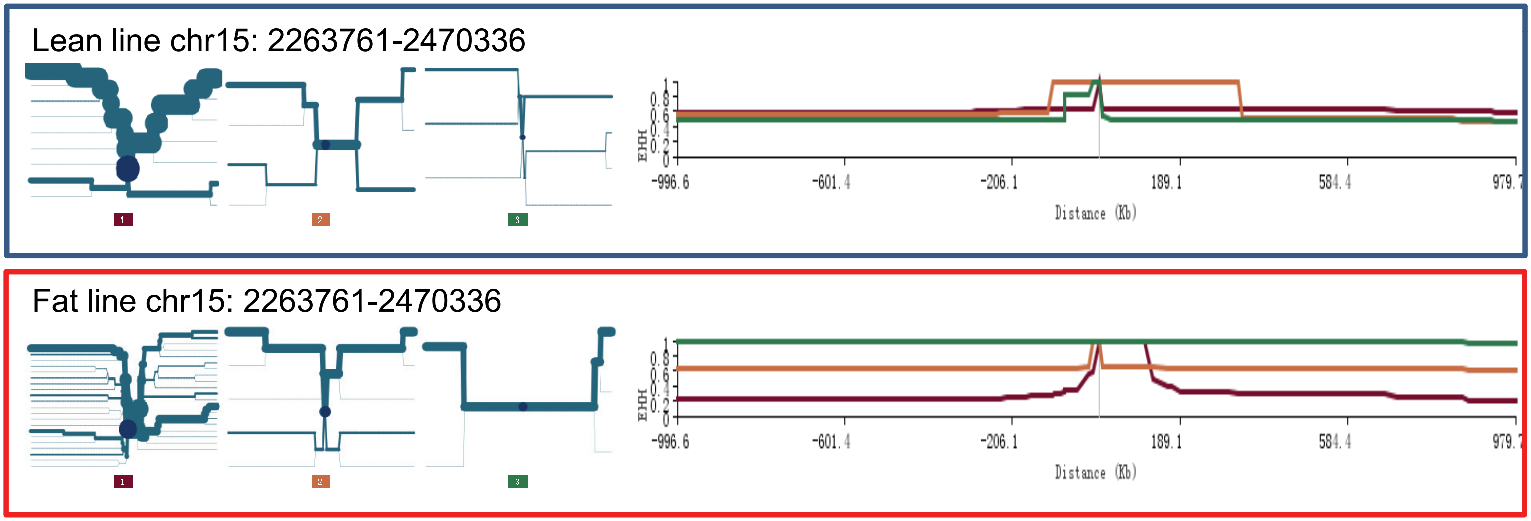
Lean line chr15: 2263761-2470336
1
2
3
EHH
-996.6
-601.4
-206.1
189.1
584.4
979.7
Distance (Kb)
Fat line chr15: 2263761-2470336
1
2
3
EHH
-996.6
-601.4
-206.1
189.1
584.4
979.7
Distance (Kb)

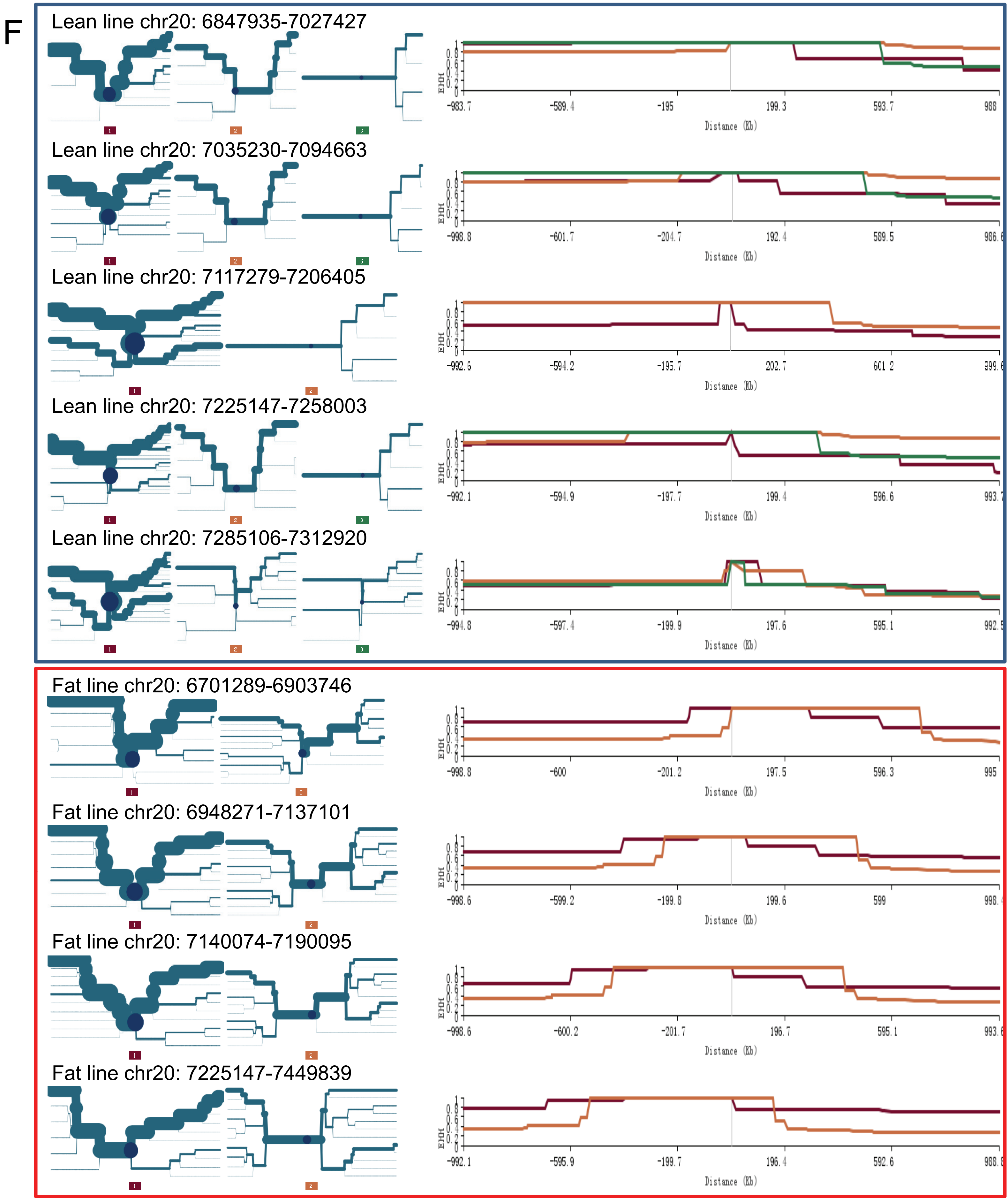

F
Lean line chr20: 6847935-7027427
Lean line chr20: 7035230-7094663
Lean line chr20: 7117279-7206405
Lean line chr20: 7225147-7258003
Lean line chr20: 7285106-7312920
Fat line chr20: 6701289-6903746
Fat line chr20: 6948271-7137101
Fat line chr20: 7140074-7190095
Fat line chr20: 7225147-7449839
EHH
Distance (Kb)

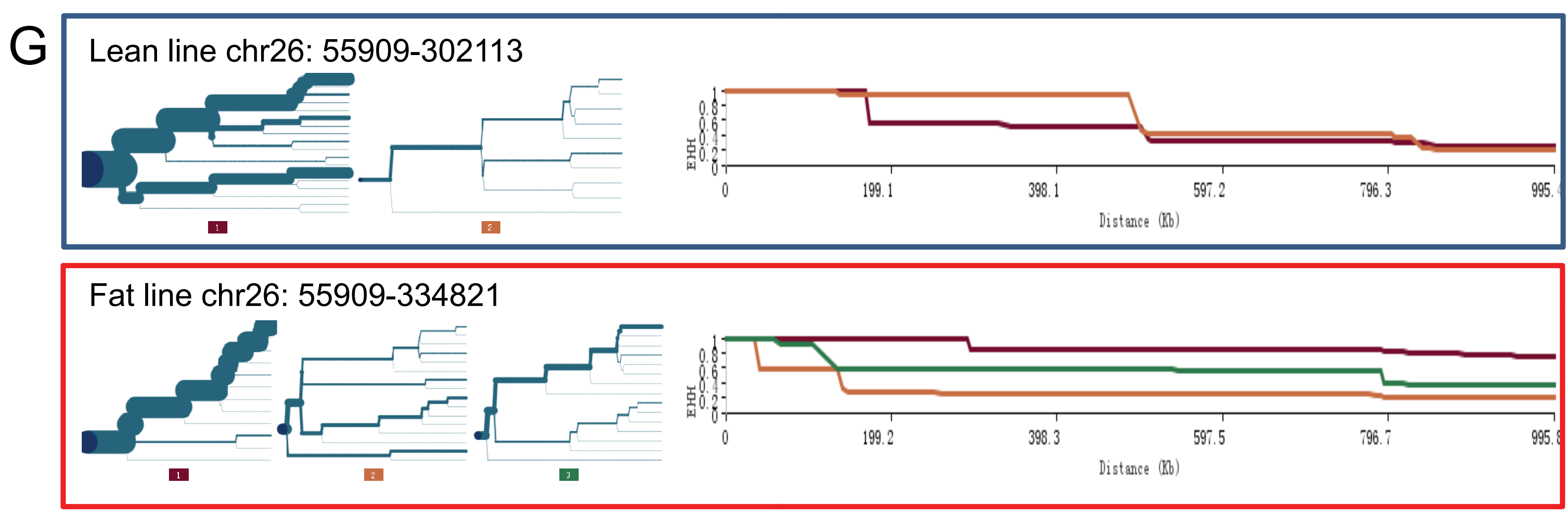
G
Lean line chr26: 55909-302113
1
2
EHH
0
0.2
0.4
0.6
0.8
1
0
199.1
398.1
597.2
796.3
Distance (Kb)
Fat line chr26: 55909-334821
1
2
3
EHH
0
0.2
0.4
0.6
0.8
1
0
199.2
398.3
597.5
796.7
Distance (Kb)
