# Supplementary material for: Selection Signature Analysis Implicates the PC1/PCSK1 Region for Chicken Abdominal Fat Content
Source: PLoS One. 2012 Jul 11;7(7):e40736. doi: 10.1371/journal.pone.0040736 (PMC3394724; doi:10.1371/journal.pone.0040736)
Supplement: Table S1 — Full gene names of candidate genes for chicken abdominal fat content. The genes in Table 3 and genes in black and blue colors in Figure S1 are included. Human obesity genes are not included, except PC1/PCSK1. (DOC) [file pone.0040736.s005.doc]

| Gene symbol | Gene name | Chromosome | Position |
| --- | --- | --- | --- |
| *PAH* | phenylalanine hydroxylase | 1 | 57119389-57156075 |
| *IGF1* | Insulin-like growth factor I | 1 | 57327750-57376178 |
| *GBE1* | glucan (1,4-alpha-), branching enzyme 1 | 1 | 98487482-98670065 |
| *TRPC4* | transient receptor potential cation channel, subfamily C, member 4 | 1 | 176097670-176235464 |
| *CAD10* | Cadherin-10 | 2 | 73523999-73621549 |
| *GJD4* | gap junction protein, delta 4 | 2 | 12756816-12760762 |
| *CCNY* | cyclin Y | 2 | 12774998-12818966 |
| *CSMD3* | CUB and Sushi multiple domains 3 | 2 | 138982510-139375858 |
| *MYT1L* | myelin transcription factor 1-like | 3 | 95860590-96152889 |
| *DDX26B* | DEAD/H (Asp-Glu-Ala-Asp/His) box polypeptide 26B | 4 | 4140961-4175706 |
| *NTCP7* | Sodium/bile acid cotransporter 7 | 4 | 32523115-32675326 |
| *IGFBP7* | insulin-like growth factor binding protein 7 | 4 | 50637829-50654573 |
| *LEPROTL1* | leptin receptor overlapping transcript-like 1 | 4 | 50714780-50717850 |
| *NDST4* | N-deacetylase/N-sulfotransferase (heparan glucosaminyl) 4 | 4 | 57673334-57758645 |
| *SGCZ* | sarcoglycan zeta | 4 | 65925913-66074306 |
| *NOVA1* | neuro-oncological ventral antigen 1 | 5 | 34863690-35327411 |
| *KIF26A* | kinesin family member 26A | 5 | 53294356-53322261 |
| *NR4A2* | nuclear receptor subfamily 4, group A, member 2 | 7 | 37664279-37668026 |
| *BAZ2B* | Bromodomain adjacent to zinc finger domain protein 2B | 7 | 38189391-38269262 |
| *ESRP2* | Epithelial splicing regulatory protein 2 | 11 | 3224004-3267556 |
| *GALR1* | Galanin receptor type 1 | 11 | 3398224-3415106 |
| *TMCC1* | transmembrane and coiled-coil domain family 1 | 12 | 20260820-20318451 |
| *FGD5* | FYVE, RhoGEF and PH domain containing 5 | 12 | 20438694-20514892 |
| *GRM7* | glutamate receptor, metabotropic 7 | 12 | 19495863-19695965 |
| *GHR* | ghrelin preproprotein | 12 | 20094804-20097505 |
| *GALNT9* | UDP-N-acetyl-alpha-D-galactosamine:polypeptide N-acetylgalactosaminyltransferase 9 | 15 | 2393778-2485829 |
| *DAB2IP* | DAB2 interacting protein | 17 | 9196449-9315057 |
| *MYBB* | Myb-related protein B | 20 | 3597679-3613736 |
| *SYCP2* | synaptonemal complex protein 2 | 20 | 6806296-6831913 |
| *CADH4* | Cadherin-4 | 20 | 7213069-7627166 |
| *TULP1* | Tubby-like 1 | 26 | 57724-64161 |
| *KIF21B* | kinesin family member 21B | 26 | 292689-311684 |
| *KCND3* | potassium voltage-gated channel, Shal-related subfamily, member 3 | 26 | 3124786-3214381 |
| *ENSGALG00000020554* | / | Z | 33135375-33247200 |
| *Mar-03* | membrane-associated ring finger (C3HC4) 3 | Z | 55111989-55463803 |
| *SLC12A2* | solute carrier family 12 (sodium/potassium/chloride transporters), member 2 | Z | 55570989-55625946 |
| *FBN2* | Fibrillin-2 | Z | 55647616-55839268 |
| *ERAP1* | Putative uncharacterized protein | Z | 55885795-55898916 |
| *CAST* | Calpastatin | Z | 55902192-55956391 |
| *PC1;PCSK1* | prohormone convertase 1; proprotein convertase subtilisin/kexin type 1 | Z | 56014540-56043792 |
| *ELL2* | elongation factor, RNA polymerase II, 2 | Z | 56157463-56212769 |
