# Supplementary material for: Selection Signature Analysis Implicates the PC1/PCSK1 Region for Chicken Abdominal Fat Content
Source: PLoS One. 2012 Jul 11;7(7):e40736. doi: 10.1371/journal.pone.0040736 (PMC3394724; doi:10.1371/journal.pone.0040736)
Supplement: Table S2 — Human obesity genes on the chicken genome. (DOC) [file pone.0040736.s006.doc]

| Obesity gene | Human genome position | Chicken genome position | References |
| --- | --- | --- | --- |
| *CADM2* | chr3:85008132-86117944 | chr1:96673462-96939780 | 2, 3 |
| *MTIF3* | chr13:28009776-28024739 | chr1:180554942-180557911 | 2, 3 |
| *MC4R* | chr18:58038564-58040001 | chr2:70267038-70268033 | 2-5 |
| *FANCL* | chr2:58386378-58468507 | chr3:891596-920584 | 2, 3 |
| *TMEM18* | chr2:667335-677439 | chr3:94980647-94985338 | 2-5 |
| *RBJ* | chr2:25166505-25194 | chr3:108219608-108226229 | 2, 3 |
| *TFAP2B* | chr6:50786436-50815326 | chr3:111026380-111052692 | 2, 3 |
| *SLC39A8* | chr4:103172198-103352415 | chr4:62536232-62563686 | 2, 3 |
| *GNPDA2* | chr4:44703885-44728612 | chr4:69487594-69494669 | 2, 3, 5 |
| *LIN7C* | chr11:27516123-27528320 | chr5:3731017-3743535 | 4 |
| *BDNF* | chr11:27676440-27743605 | chr5:3774758-3775498 | 2-4 |
| *RPL27A* | chr11:8703958-8736306 | chr5:10702394-10705049 | 2, 3 |
| *PRKD1* | chr14:30045687-30661104 | chr5:36186817-36300669 | 2, 3 |
| *NRXN3* | chr14:78709437-80330758 | chr5:41990460-42890561 | 2, 3 |
| *LRP1B* | chr2:140988992-142889270 | chr7:33466517-33901152 | 2, 3 |
| *RASAL2* | chr1:178062864-178448644 | chr[8:6757285-6837504](http://www.ensembl.org/Gallus_gallus/Location/View?r=8:6757285-6837504:-1;g=ENSGALG00000004323) | 4 |
| *SEC16B* | chr1:177897923-178007142 | chr8:6926277-6937587 | 2-4 |
| *PTBP2* | chr1:97187221-97280349 | chr8:13643547-13674010 | 2, 3 |
| *NEGR1* | chr1:71868625-72748417 | chr8:29962352-30046009 | 2-5 |
| *TNNI3K* | chr1:74663922-74699770 | chr8:30360409-30413231 | 2, 3 |
| *SFRS10* | chr3:185633694-185655924 | chr9:5425413-5443739 | 4 |
| *ETV5* | chr3:185764097-185828107 | chr9:6172639-6181977 | 2-4 |
| *DGKG* | chr3:185823457-186080026 | chr9:6186383-6198550 | 4 |
| *MAP2K5* | chr15:67835021-68116181 | chr10:21102233-21225083 | 2, 3 |
| *MMP2* | chr16:55512883-55540603 | chr11:3680958-3716263 | 6,7 |
| *FTO* | chr16:53737875-54155853 | chr11:4846977-4892992 | 2-5 |
| *RPGRIP1L* | chr16:53633824-53737771 | chr11:4945427-5016934 | 4 |
| *CHST8* | chr19:34112861-34264413 | chr11:11428680-11430242 | 4 |
| *KCTD15* | chr19:34287751-34306668 | chr11:11465973-11506379 | 2-5 |
| *GPRC5B* | chr16:19868616-19896832 | chr14:15445797-15452775 | 2, 3 |
| *NUDT3* | chr6:34255997-34360451 | chr26:4218024-4238093 | 2, 3 |
| *PC1/* *PCSK1* | chr5:95726119-95769847 | chrZ:56014540-56043792 | 8-12 |
| *ZNF608* | chr5:123972608-124084500 | chrZ:73032178-73076141 | 2, 3 |
| *MTCH2* | chr11:47638867-47664175 | Un_random:14925240-14926389 | 2-5 |
| *QPCTL* | chr19:46195741-46207240 | Un_random:14931707-14933732 | 2, 3 |
| *FAIM2* | chr12:50260679-50298000 | [E22C19W28_E50C23:774631-790235](http://www.ensembl.org/Gallus_gallus/Location/View?r=E22C19W28_E50C23:774631-790235:-1;g=ENSGALG00000010254) | 2, 3 |
| *BCDIN3D* | chr12:50231573-50236912 | E22C19W28_E50C23:763366-766761 | 4 |
