# Supplementary material for: Selection Signature Analysis Implicates the PC1/PCSK1 Region for Chicken Abdominal Fat Content
Source: PLoS One. 2012 Jul 11;7(7):e40736. doi: 10.1371/journal.pone.0040736 (PMC3394724; doi:10.1371/journal.pone.0040736)
Supplement: Table S3 — Haplotype frequencies in selection signatures. (DOC) [file pone.0040736.s007.doc]

| Position and gene region  of selection signature | Haplotype  Number | Haploptypes | Haplotype frequency in lean line | Haplotype frequency in fat line |
| --- | --- | --- | --- | --- |
| chr1: 57053708-57160808  *PAH-IGF1* | 1 | GGAA | *0.392* | *0.984* |
|  | 2 | AAGA | *0.329* | 0 |
|  | 3 | GAAG | 0.213 | 0.016 |
|  | 4 | AGAA | 0.065 | 0 |
| chr1: 176076327-176286631  *TRPC4* | 1 | GCGACGAG | *0.481* | 0 |
|  | 2 | ACGAAGAA | 0.302 | 0 |
|  | 3 | ACGAAAGA | 0.072 | 0.093 |
|  | 4 | ACAAAGAG | 0.059 | 0.031 |
|  | 5 | GCGAAAGG | 0.055 | 0.016 |
|  | 6 | GAGAAGAA | 0.032 | *0.462* |
|  | 7 | GCAGAAGA | 0 | 0.284 |
|  | 8 | ACGAAAGG | 0 | 0.108 |
| chr2: 12476376-12850042  *GJD4, CCNY* | 1 | CAAGAGAAGAGAAA | *0.785* | 0.049 |
|  | 2 | CGGAGGAGGGGACG | 0.074 | 0.080 |
|  | 3 | CAAGAGAAGAAGCG | 0.057 | 0 |
|  | 4 | CGGAGGAAGAAGCG | 0.044 | 0 |
|  | 5 | AGAGGGAAGAAGCG | 0.040 | 0 |
|  | 6 | CGGGGGAAGGGGCG | 0 | *0.312* |
|  | 7 | AGGAAGGGAGGGCG | 0 | 0.245 |
|  | 8 | AAAGGAAGGAGACG | 0 | 0.109 |
|  | 9 | CGGAGGGGGAAGCG | 0 | 0.082 |
|  | 10 | AAAGAGAGGGGAAG | 0 | 0.062 |
|  | 11 | CGGGGGAGGAGGAA | 0 | 0.023 |
|  | 12 | CGGGGGAGGAGAAA | 0 | 0.020 |
|  | 13 | AGGAAGGGAGGAAA | 0 | 0.013 |
| chr4: 57429243-57788219  *NDST4* | 1 | AGAAAAAGAAGCGA | *0.742* | 0.160 |
|  | 2 | AAGGGGAGGGACAG | 0.153 | *0.299* |
|  | 3 | GGAAAAAGAAGCGA | 0.098 | 0 |
|  | 4 | GAGGGGAAGGAAAG | 0 | 0.155 |
|  | 5 | AGAAGGGGGGACAG | 0 | 0.116 |
|  | 6 | AAGGGGGAGGGAAG | 0 | 0.078 |
|  | 7 | AGAAGGGGGAAAAG | 0 | 0.057 |
|  | 8 | GGGGGGAGGGACAG | 0 | 0.051 |
|  | 9 | GGGAGGAAGGGCGG | 0 | 0.051 |
|  | 10 | GAGGGGGAGGGAAG | 0 | 0.033 |
| chr5: 35024640-35653631  *NOVA1* | 1 | AGGAGACAGGAAG | *0.677* | 0.018 |
|  | 2 | GAGGAGCAGGAAG | 0.148 | 0.096 |
|  | 3 | GAAAAGAGAAAAA | 0.141 | 0.235 |
|  | 4 | GAGGAGAGAGAGG | 0.034 | 0 |
|  | 5 | GAAGAGAGAGGGA | 0 | *0.270* |
|  | 6 | AGGAGACAGGGGA | 0 | 0.173 |
|  | 7 | GAAGAGAGAGAGG | 0 | 0.082 |
|  | 8 | GAAGGACAGGAAG | 0 | 0.080 |
|  | 9 | AAAGAGAGAAAGG | 0 | 0.044 |
| chr11: 3196613-3402854  *ESRP2-GALR1* (5 genes) | 1 | GAACAAAAG | *0.795* | *0.529* |
|  | 2 | GAGCAAAGG | 0.175 | 0.098 |
|  | 3 | GAACAAAGG | 0.030 | 0.013 |
|  | 4 | ACAAGGGGA | 0 | *0.359* |
| chr15: 2155345-2495806  *GALNT9* | 1 | AGAAAGGCAGAAGAGCACAAACGCAAGG | *0.432* | 0.039 |
|  | 2 | AAGGCGGAAGAAGAAAGCGGGCACAGGG | 0.200 | 0.034 |
|  | 3 | AGAACGACAGAAGAGCACAAACGCAGGG | 0.135 | 0 |
|  | 4 | AGGGAGAAGACGAGAAGAGGGAGCAGAA | 0.070 | 0.054 |
|  | 5 | AGAAAGGCAGAAGAAAGCGGGCACAGGG | 0.061 | 0 |
|  | 6 | AGAAAGGAAGAAGGAAGCGGGCGCGAAG | 0.044 | 0.067 |
|  | 7 | AAAGAGAAGACGAGAAGAGGGAGCAAGG | 0.027 | 0 |
|  | 8 | AGAACGACAGAAGAAAGCAAACGCGAAA | 0.015 | 0 |
|  | 9 | AGGGAAAAGACGAGAAGAGGGAGCAAGA | 0.011 | *0.192* |
|  | 10 | AGGGCGAAAGAAGAAAGAGGGCGCGGAA | 0 | *0.192* |
|  | 11 | AGGGAAAAGACGAGAAGAGGGAGCAAGA | 0 | 0.185 |
|  | 12 | GGGGCAGAGACGAGAAGAGGGAGAAGAA | 0 | 0.093 |
|  | 13 | GGGGCAAAGACGAGAAGAGGGAGAAGAA | 0 | 0.049 |
|  | 14 | AAGGCAAAGACGAGAAGAGGGAGAAGAA | 0 | 0.049 |
|  | 15 | GAAGCAGAGACGAGAAGAGGGAGAAGAA | 0 | 0.028 |
| chr20: 6829844-7289095  *SYCP2-CADH4* (6genes) | 1 | AAGGGGGGAAGACAGAAGGGGGCCAAAGAAAAGGGAAACGGGGAGGAC | *0.402* | 0.086 |
|  | 2 | AAGGGGGGAAGGCAAAAGGAGGCCAAAGAAAAGGGAAACAGAGAAGAC | 0.222 | 0 |
|  | 3 | GCAAAAAAACAAAAGGAGAGAACAGGGGAGACAGGCGGAGAGAAGAAA | 0.148 | 0.314 |
|  | 4 | AAGGGGGGAAGACAGAAGGGGGCCAAAGAAAAGGGAAACGGGGAGAGC | 0.090 | 0 |
|  | 5 | AAGGAGGAACAACAAAAGGAGGAAGGGGGGGAGGGCGGAGGGGAGGGC | 0.057 | 0 |
|  | 6 | AAGGGGGGACGACAGAAGGGGGCCAGGGGGACAGACGGAAGAGGGAAA | 0.027 | 0 |
|  | 7 | GAGGGGGGAAGGCAAAAGGAGGCCAAAGAAAAGGGAAACAGAGAAGAC | 0.023 | 0 |
|  | 8 | GCAAAAGGACGACAGAAGGGGGCCAGGGGGACAGACGGAGGGGAGGAA | 0.014 | 0 |
|  | 9 | GCGGAAAGGAAACGGGGAAGGAAAGAGAGGGAGAACGACAGAGGGAAA | 0 | *0.525* |
|  | 10 | GCGGAAAGGAAACGGGGAAGGAAAGAGAGGGAGAACGGAGGGAAGAAA | 0 | 0.064 |
| chr26: 55909- 288827  *TULP-LOC421178* (14 genes) | 1 | AGAAGAGAAGCGAA | *0.808* | 0.253 |
|  | 2 | AGAAGGGAAGCGAA | 0.093 | 0.172 |
|  | 3 | GAAGAGGGAACAAA | 0.059 | 0 |
|  | 4 | GAAGAGGGAGCGAA | 0.040 | 0 |
|  | 5 | GAGGAGAGGAAAGG | 0 | *0.539* |
|  | 6 | AGAAGGGAAGCGAG | 0 | 0.022 |
| chrZ: 55428021-56164905  *Mar-03*, *SLC12A*, *FBN2*, *ERAP1*,  *CAST*, *PC1*, *ELL2* | 1 | AAGGAAGGACGAAGAGGGAAGAAAAG | *0.683* | 0 |
|  | 2 | GGAAGCAACAAGAGAGGGAAGAAAAG | 0.193 | 0.196 |
|  | 3 | AAGAAAGGACGAAGAGGGAAGAAAAG | 0.063 | 0 |
|  | 4 | AAGAGAAACCAGGAAGAAAGAGGAAG | 0.043 | 0 |
|  | 5 | GGAAGCAACAAGGAGAAAGGAGGGGA | 0.017 | *0.750* |
|  | 6 | AAGAGAAGACGAGAAGAAGGAGGGGA | 0 | 0.052 |
